# Global, regional, and national burden of benign prostatic hyperplasia from 1990 to 2021 and projection to 2035

**DOI:** 10.1186/s12894-025-01715-9

**Published:** 2025-02-19

**Authors:** Hui Wei, Cong Zhu, Qiao Huang, Jun Yang, Yi-Tong Li, Yin-Gang Zhang, Bing-Hui Li, Hao Zi

**Affiliations:** 1 Administrative Office of President, The First People’s Hospital of Tianmen in Hubei Province, The Affiliated Hospital of Hubei University of Science and Technology, Tianmen, China; 2 Center for Evidence-Based and Translational Medicine, Zhongnan Hospital of Wuhan University, Wuhan, China; 3 Department of Urology, Zhongnan Hospital of Wuhan University, Wuhan, China; 4 Department of Urology, The First People’s Hospital of Tianmen in Hubei Province, The Affiliated Hospital of Hubei University of Science and Technology, Tianmen, China; 5 School of Clinical Medicine, Hubei University of Arts and Science, Xiangyang, Hubei China; 6 Department of Healthcare Management (Physical Examination Center), Zhongnan Hospital of Wuhan University, Wuhan, 430071 China; 7 Evidence-Based Medicine Center, Xiangyang No. 1 People’s Hospital, Hubei University of Medicine, Xiangyang, 441000 China

**Keywords:** Benign prostatic hyperplasia, Prevalence, Incidence, Disability-adjusted life-years, Burden of disease

## Abstract

**Background:**

Benign prostatic hyperplasia (BPH) is a common male urological disease around the world. This study aimed to evaluate global, regional, and national burden of BPH from 1990 to 2021, and to forecast the incidence and prevalence of BPH to 2035.

**Methods:**

Using the data and methods of the Global Burden of Disease 2021, we presented the incidence, prevalence, and disability-adjusted life-years (DALYs) of BPH from 1990 to 2021. The trends of burden over time were assessed using estimated annual percentage changes. We applied Bayesian age-period-cohort model to forecast the incidence and prevalence of BPH to 2035.

**Results:**

In 2021, the global number of incident cases, prevalent cases, and DALYs of BPH were 137.88, 1125.02, and 22.36 per 100,000 populations, respectively. From 1990 to 2021, the age-standardized incidence rate (ASIR), age-standardized prevalence rate (ASPR), and age-standardized DALYs rate (ASDR) of BPH remained stable. The highest ASIR, ASPR, and ASDR were recorded in Eastern Europe in 2021. Nationally, China had the highest number of incident cases, prevalent cases, and DALYs of BPH. With the increase of socio-demographic index, the trends of ASIR, ASPR, and ASDR all exhibit an initial rise followed by a gradual decline. The global incidence and prevalence are expected to increase from 962.42 to 7878.68 per 100,000 populations in 2022 to 998.55 and 8620.60 per 100,000 populations in 2035, respectively.

**Conclusions:**

The persistent burden of BPH continues to pose a critical public health challenge. The escalating prevalence among middle-aged and elderly populations underscores the imperative to tackle this widespread condition.

**Supplementary Information:**

The online version contains supplementary material available at 10.1186/s12894-025-01715-9.

## Background

Benign prostatic hyperplasia (BPH) is a common male urological disease around the world [1]. BPH is histologically defined by the enlargement of glandular epithelial tissue, smooth muscle, and connective tissue within the prostatic transition zone [2]. Among males aged 50 and above, the prevalence of BPH ranges from 50 to 75%, with a notable increase to 80% in those aged 70 [3]. Among elderly males, BPH is associated with severe health complications, including increased vulnerability to falls, depression, and a decline in health-related quality of life [4–6]. BPH has emerged as a significant public health concern, particularly amidst the accelerated global ageing, as it contributes to substantial hikes in healthcare costs and a notable decline in the quality of life.

A meta-analysis that included data from 25 countries found the global prevalence of BPH is 26.2%, with no significant differences between countries [7]. In addition, the prevalence of BPH has not changed significantly over the three study periods: 26.6% in the period 1990–1999, 27.8% in the period 2000–2009 and 22.8% in the period 2010–2015. Based on Global Burden of Disease (GBD) 2017 estimates, the years lived with disability (YLDs) attributed to BPH have risen from 1.35 million in 1990 to 2.43 million in 2017 [8]. The subset analyses by socio-demographic index (SDI) status show significant differences between the highest and lowest members of sociodemographic strata. Recent studies indicate that the global burden of disease associated with BPH exhibits substantial variation, particularly among nations with varying degrees of development [9–12]. Limited studies have explored the geographical variation in the global burden of disease associated with BPH, yet there is a lack of comprehensive analysis of temporal trends and predictions of future trends for BPH. Therefore, there is a need to update the epidemiological data on BPH globally to inform disease prevention and control policies.

The GBD 2021 offers an updated compilation of data related to the global incidence, prevalence, YLDs, and disability-adjusted life-years (DALYs) for 371 diseases and injuries in 204 countries and territories from 1990 to 2021 [13, 14]. The GBD database is one of the key data sources for global health research, providing timely and comprehensive estimates of the disease burden, which are vital for making health-related policy decisions [15]. In this study, we present the incidence, prevalence, and DALYs of BPH by global, regional, national, SDI, and age from 1990 to 2021. Additionally, we provide predictions for the global number and crude rate of incidence and prevalence for BPH up to 2035. The objectives of this study are to provide policymakers and practitioners with scientific and evidence-grounded insights, thus facilitating informed decision-making, priority setting, and resource allocation across various levels, spanning from the subnational to the global spectrum.

## Methods

### Data sources

In this study, we obtained data on incidence, prevalence, and DALYs of BPH by global, regional, national, SDI, and age from the GBD 2021. The Institute for Health Metrics and Evaluation at the University of Washington provided all the data for GBD 2021, and all the data can be found on the Global Health Data Exchange website (https://vizhub.healthdata.org/gbd-results). The GBD 2021 estimated incidence, prevalence, years lived with disability (YLDs), years of life lost (YLLs), and DALYs for 371 diseases and injuries [13]. GBD 2021 data were extracted from vital registration systems, verbal autopsies, censuses, household surveys, disease-specific registries, health service contact data, and other sources [13, 14, 16]. Counts and age-standardized rates (ASR) were calculated globally, 21 regions, and 204 countries and territories, from 1990 to 2021. All estimates were generated with 95% uncertainty interval (UI), which were determined using the 2.5 and 97.5th values of the ordered 500 draws. The detailed methodologies of the GBD 2021 have been provided elsewhere [13, 14, 16]. BPH in the GBD 2021 was defined based on the International Classification of Diseases of Tenth Revision (ICD-10) codes of N40, N40.0, N40.1, N40.2, N40.3, and N40.9 and ICD-9 codes of 600, 600.0, 600.1, 600.2, 600.3, and 600.9 12. Since GBD 2021 did not estimate mortality for BPH, it consequently did not calculate YLLs for BPH. Therefore, DALYs equalled YLDs.

### Statistical analysis

Trends over time were assessed using the estimated annual percentage changes (EAPC) with 95% confidence interval (CI), which was achieved through a linear regression model based on the equation Y = α + βX + ε, where Y was the natural logarithm of ASR, X was the calendar year, and ε stood for the error term [12, 17]. It is shown that when EAPC and the lower boundary of the 95% CI are positive, then ASR is in an upward trend. Conversely, when EAPC and the upper boundary of the 95% CI are negative, the ASR shows a descending trend. Additionally, the Bayesian age-period-cohort (BAPC) package was utilized to predict the number and crude rate of incidence and prevalence for BPH up to 2035. Previous studies have showed that BAPC model gave well calibrated projection and not too wide uncertainty interval [18, 19]. Statistical analysis and data visualization were conducted using R software (version: V.4.3.2) and Microsoft Excel (Version 2019).

**Table 1:** Incidence and age-standardized incidence rate of benign prostatic hyperplasia between 1990 and 2021

Characteristics	All ages, No. ×10^5^ (95% UI)			Change (%)		Age-standardized rate per 100,000, No. (95% UI)			EAPC (95% CI)
	1990	2021		1990–2021		1990	2021		1990–2021
**Global**	64.06(50.00 to 79.95)	137.88(109.08 to 170.15)		115.23		335.00 (262.28 to 414.71)	326.12(258.88 to 400.32)		0.03(-0.02 to 0.08)
**Socio-demographic index**									
High SDI	11.26(9.02–13.86)	20.84(17.17–25.23)		85.01		230.69(185.05-283.09)	223.24(185.43-269.31)		0.15(0.05 to 0.24)
High-middle SDI	17.78(13.8-22.18)	32.51(25.58–40.12)		82.86		387.86(304.55–477.5)	341.28(270.68-417.58)		-0.32(-0.37 to -0.28)
Middle SDI	19.62(15.12–24.81)	49.25(38.69–61.1)		151.06		383.38(296.85-477.53)	365.42(287.67-451.19)		0.04(-0.03 to 0.12)
Low-middle SDI	11.73(9.1-14.83)	27.05(20.94–34.05)		130.6		364.28(283.11-453.67)	377.12(292.19-470.61)		0.11(0.1 to 0.11)
Low SDI	3.59(2.74–4.67)	8.1(6.23–10.24)		125.49		297.47(227.57-379.23)	316.96(244.62-400.47)		0.16(0.14 to 0.17)
**Region**									
Andean Latin America	0.32(0.25–0.42)	0.96(0.73–1.23)		197.61		326.01(248.02–417.7)	338.46(255.41-429.17)		0.18(0.15 to 0.22)
Australasia	0.27(0.2–0.35)	0.59(0.44–0.77)		122.92		237.48(181.15-310.93)	238.76(181.61-306.07)		0.10(0.01 to 0.20)
Caribbean	0.44(0.33–0.56)	0.93(0.71–1.18)		113.14		343.5(259.87-438.46)	361.16(277.09-456.08)		0.21(0.19 to 0.24)
Central Asia	0.62(0.47–0.8)	1.26(0.96–1.62)		103.78		322.95(246.39-412.09)	329.97(254.99-415.88)		0.02(-0.01 to 0.05)
Central Europe	2.89(2.3–3.52)	3.71(3.02–4.42)		28.73		421.71(338.85-510.06)	376.63(310.49-445.07)		-0.22(-0.28 to -0.16)
Central Latin America	2.06(1.64–2.53)	6.22(4.95–7.65)		201.74		511.9(404.48-626.39)	522.07(416.84-639.17)		0.15(0.12 to 0.18)
Central Sub-Saharan Africa	0.27(0.2–0.35)	0.66(0.49–0.88)		146.47		241.82(182.85-313.99)	246.34(187.34-322.96)		0.03(0.02 to 0.05)
East Asia	15.25(11.33–19.69)	33.96(26.04–42.46)		122.61		365.7(276.5-465.01)	303.48(236.84-380.16)		-0.21(-0.35 to -0.06)
Eastern Europe	7.21(5.64-9)	9.59(7.52–11.75)		33.12		662.35(525.35-796.88)	661.12(527.06-792.25)		-0.02(-0.05 to 0.01)
Eastern Sub-Saharan Africa	1.06(0.8–1.37)	2.29(1.75–2.94)		116.64		272.19(207.49-345.69)	275.75(209.05-352.62)		0.02(0.01 to 0.03)
High-income Asia Pacific	1.42(1.08–1.85)	2.53(1.94–3.31)		77.86		149.78(113.51-194.95)	138.23(106.16-179.65)		-0.08(-0.18 to 0.01)
High-income North America	2.98(2.43–3.61)	6.47(5.44–7.58)		116.96		200.49(164.63-240.78)	216.18(183.87-251.08)		0.47(0.38 to 0.56)
North Africa and Middle East	2.12(1.61–2.76)	6.04(4.63–7.76)		184.62		238.93(182.26-306.39)	250.22(191.45-319.89)		0.13(0.12 to 0.14)
Oceania	0.06(0.05–0.08)	0.17(0.13–0.21)		163.06		423.34(323.96-530.63)	437.75(333.86-548.82)		0.12(0.10 to 0.13)
South Asia	11.77(8.98–14.97)	31.26(24.08–39.42)		165.6		373.12(288.78-469.22)	412.9(319.88-516.15)		0.21(0.16 to 0.25)
Southeast Asia	6.37(4.99–7.9)	15.4(12.11–19.21)		141.65		512.53(402.25-630.31)	467.42(367.61-577.87)		-0.10(-0.18 to -0.03)
Southern Latin America	0.32(0.25–0.42)	0.65(0.49–0.83)		99.04		151.18(115.48-193.62)	164.39(124.74-211.33)		0.38(0.28 to 0.48)
Southern Sub-Saharan Africa	0.4(0.31–0.52)	0.89(0.68–1.16)		121.6		331.08(253.71-418.71)	342.67(265.2-433.79)		0.11(0.09 to 0.13)
Tropical Latin America	1.18(0.96–1.44)	2.69(2.22–3.33)		128.5		266.66(217.61-325.57)	222.87(183.01-273.81)		-0.25(-0.36 to -0.14)
Western Europe	5.88(4.68–7.25)	9.18(7.38–11.21)		56.12		237.48(190.64-291.62)	241.55(197-291.87)		0.38(0.25 to 0.50)
Western Sub-Saharan Africa	1.17(0.89–1.51)	2.42(1.85–3.1)		107.13		248.54(189-319.76)	250.9(191.94-320.18)		0.01(-0.01 to 0.01)

SDI: socio-demographic index; EAPC: estimated annual percentage change; UI: uncertainty interval; CI: confidence interval

**Table 2:** Prevalence and age-standardized prevalence rate of benign prostatic hyperplasia between 1990 and 2021

Characteristics	All ages, No. ×10^5^ (95% UI)			Change (%)		Age-standardized rate per 100,000, No. (95% UI)			EAPC (95% CI)
	1990	2021		1990–2021		1990	2021		1990–2021
**Global**	507.06(387.36 to 656.93)	1125.02(881.32 to 1426.34)		121.87		2899.84(2240.05 to 3682.58)	2782.59(2191.58 to 3508.04)		-0.01(-0.06 to 0.04)
**Socio-demographic index**									
High SDI	96.86(76.99 to 121.9)	187.79(155.4 to 227.86)		93.89		2036.64(1630.29 to 2549.71)	1927.97(1605.03 to 2322.83)		0.03(-0.04 to 0.1)
High-middle SDI	138.45(106.36 to 177.68)	262.59(205.04 to 330.79)		89.67		3335.89(2606.02 to 4198.18)	2881.35(2275.34 to 3621.68)		-0.38(-0.42 to -0.33)
Middle SDI	147.45(111.31 to 192.56)	386.37(298.3 to 493.36)		162.03		3282.19(2531.05 to 4187.81)	3097.5(2433.52 to 3911.51)		0.02(-0.06 to 0.1)
Low-middle SDI	95.18(71.11 to 126)	222.6(166.71 to 289.73)		133.87		3300.7(2462.56 to 4294.15)	3388.59(2569.07 to 4372.07)		0.12(0.11 to 0.13)
Low SDI	28.46(21.16 to 38.58)	64.57(48.5 to 85.1)		126.90		2651.54(1995.11 to 3512.02)	2837.28(2142.2 to 3707.99)		0.18(0.17 to 0.19)
**Region**									
Andean Latin America	2.59(1.92 to 3.49)	7.91(5.82 to 10.37)		205.53		2790.92(2076.26 to 3700.93)	2877.23(2127.17 to 3752.32)		0.15(0.11 to 0.18)
Australasia	2.14(1.59 to 2.87)	5.21(3.85 to 6.85)		143.28		1978.39(1492.03 to 2639.12)	1979.78(1482.19 to 2581.41)		0.03(-0.01 to 0.07)
Caribbean	3.6(2.67 to 4.77)	7.69(5.65 to 10.02)		113.33		2910.45(2157.49 to 3849.88)	3060.81(2253.52 to 3991.28)		0.19(0.18 to 0.21)
Central Asia	4.72(3.47 to 6.27)	9.43(7.09 to 12.44)		100		2753.11(2059.41 to 3600.03)	2826.87(2151.52 to 3627.82)		0.05(0.03 to 0.07)
Central Europe	23.23(18.32 to 29.16)	31.64(26.22 to 38.35)		36.23		3671.78(2900.93 to 4562.4)	3222.04(2698.56 to 3877.95)		-0.15(-0.25 to -0.04)
Central Latin America	17.36(13.63 to 21.77)	53.74(42.42 to 66.93)		209.58		4703.37(3695.95 to 5862.59)	4775.05(3770.46 to 5939.43)		0.12(0.09 to 0.15)
Central Sub-Saharan Africa	2.04(1.49 to 2.8)	4.76(3.5 to 6.53)		133.47		2107.05(1553.6 to 2837.46)	2143.08(1598.98 to 2845.61)		0.03(0.02 to 0.04)
East Asia	103.93(75.91 to 137.52)	243.04(189.22 to 316.11)		133.85		2908.32(2165.65 to 3765.43)	2361.87(1862.45 to 2997.8)		-0.23(-0.39 to -0.08)
Eastern Europe	58.6(44.56 to 75.65)	84.97(64.48 to 107.99)		45.01		6282.71(4855.07 to 7873.65)	6262.23(4821.08 to 7834.28)		0.01(-0.02 to 0.01)
Eastern Sub-Saharan Africa	8.29(6.09 to 11.27)	17.55(12.95 to 23.75)		111.68		2393.94(1784.77 to 3200.25)	2421.32(1784.74 to 3225.77)		0.01(0.01 to 0.03)
High-income Asia Pacific	11.28(8.45 to 15.13)	24.2(18.7 to 31.35)		114.48		1296.89(979.41 to 1724.38)	1167.78(902.09 to 1524.86)		-0.15(-0.22 to -0.08)
High-income North America	27.26(21.94 to 33.42)	55.53(48.53 to 63.47)		103.7		1829.85(1479.59 to 2225.25)	1818.9(1599.15 to 2062.68)		0.24(0.17 to 0.31)
North Africa and Middle East	16.54(12.22 to 22.67)	47.06(34.71 to 63.39)		184.45		2068.76(1547.43 to 2741.6)	2168.64(1598.24 to 2888.35)		0.13(0.13 to 0.14)
Oceania	0.44(0.32 to 0.58)	1.17(0.89 to 1.53)		168.16		3494.28(2632.37 to 4480.75)	3632.8(2754.12 to 4576.18)		0.14(0.13 to 0.15)
South Asia	95.38(70.49 to 126.81)	261.16(192.77 to 339.67)		173.82		3414.35(2525.76 to 4455.64)	3749.01(2817.43 to 4827.64)		0.22(0.19 to 0.25)
Southeast Asia	51.34(38.41 to 66.56)	122.24(93.2 to 158.12)		138.09		4691.86(3557.72 to 5984.51)	4189.58(3228.55 to 5327.26)		-0.13(-0.22 to -0.03)
Southern Latin America	2.65(2 to 3.48)	5.47(4.02 to 7.25)		106.28		1292.22(983.99 to 1703.47)	1404.11(1037.67 to 1852.78)		0.33(0.29 to 0.38)
Southern Sub-Saharan Africa	3.36(2.51 to 4.55)	7.32(5.45 to 9.98)		117.48		3049.42(2294.8 to 4079.41)	3166.5(2375.95 to 4202.84)		0.12(0.10 to 0.14)
Tropical Latin America	9.34(7.81 to 11.38)	21.71(18.13 to 26.56)		132.31		2323.57(1928.58 to 2823.07)	1892.15(1570.65 to 2306.74)		-0.32(-0.43 to -0.20)
Western Europe	53.8(43.01 to 67.26)	94.36(77.31 to 115.9)		75.4		2176.64(1746.64 to 2711.01)	2269.19(1864.75 to 2771.65)		0.20(0.17 to 0.23)
Western Sub-Saharan Africa	9.18(6.74 to 12.53)	18.89(13.94 to 25.54)		105.77		2187.96(1620.36 to 2936.14)	2210.79(1638.32 to 2941.62)		0.01(0.01 to 0.02)

SDI: socio-demographic index; EAPC: estimated annual percentage change; UI: uncertainty interval; CI: confidence interval

## Results

### Global incidence, prevalence, and DALYs

In 2021, the global incident cases of BPH reached 137.88*10^5^ (95% UI 109.08 to 170.15), marking an increase of 115.23% compared to 1990 (Table 1). The age-standardized incidence rate (ASIR) of BPH stood at 326.12 per 100,000 persons, with a 95% UI from 258.88 to 400.32. Over the period from 1990 to 2021, the ASIR exhibited a stable pattern, with an EAPC of 0.03 (95% CI: -0.02 to 0.08). Globally, BPH caused 1125.02*10^5^ (95% UI 881.32 to 1426.34) cases in 2021, accompanied by an age-standardized prevalence rate (ASPR) of 2782.59 (95% UI 2191.58 to 3508.04) per 100,000 persons (Table 2). Over recent decades, the ASPR maintained a steady trend, reflected by an EAPC of -0.01 (95% CI -0.06 to 0.04). Regarding the global impact on DALYs, BPH accounted for 22.36*10^5^ DALYs in 2021, with a 95% UI from 13.46 to 34.03. This represented a substantial 121.13% increase in DALYs compared to 1990 (Table 3). Similarly, the age-standardized DALYs rate (ASDR) remained stable over the same period, with an EAPC of 0.01 (95% CI -0.05 to 0.05).

**Table 3:** DALYs and age-standardized DALYs rate of benign prostatic hyperplasia between 1990 and 2021

Characteristics	All ages, No. ×10^5^ (95% UI)			Change (%)		Age-standardized rate per 100,000, No. (95% UI)			EAPC (95% CI)
	1990	2021		1990–2021		1990	2021		1990–2021
**Global**	10.11(6.06 to 15.56)	22.36(13.46 to 34.03)		121.13		57.48(34.56 to 87.77)	55.12(33.21 to 83.48)		0.01(-0.05 to 0.05)
**Socio-demographic index**									
High SDI	1.93(1.18 to 2.94)	3.72(2.34 to 5.59)		92.69		40.45(24.8 to 61.4)	38.22(24.15 to 57.34)		0.02(-0.04 to 0.09)
High-middle SDI	2.76(1.66 to 4.22)	5.22(3.12 to 7.92)		89.18		66.14(39.87 to 99.8)	57.15(34.03 to 86.02)		-0.37(-0.41 to -0.32)
Middle SDI	2.95(1.75 to 4.58)	7.7(4.6 to 11.79)		160.69		65.14(38.98 to 98.45)	61.38(36.73 to 92.45)		0.02(-0.06 to 0.1)
Low-middle SDI	1.89(1.14 to 2.87)	4.42(2.64 to 6.85)		133.52		65.06(38.76 to 99.59)	66.81(39.95 to 102.81)		0.13(0.12 to 0.14)
Low SDI	0.57(0.33 to 0.87)	1.28(0.77 to 1.99)		127.34		52.14(30.87 to 81.1)	55.97(33.22 to 87.23)		0.2(0.19 to 0.21)
**Region**									
Andean Latin America	0.05(0.03 to 0.08)	0.16(0.09 to 0.25)		203.69		55.78(32.35 to 88.73)	57.26(33.77 to 90.69)		0.14(0.10 to 0.17)
Australasia	0.04(0.03 to 0.07)	0.1(0.06 to 0.16)		142.79		38.99(23.61 to 60.96)	39.12(23.57 to 60.97)		0.03(0.01 to 0.07)
Caribbean	0.07(0.04 to 0.11)	0.15(0.09 to 0.24)		111.52		58.21(34.46 to 91.96)	60.73(36.75 to 95.66)		0.17(0.15 to 0.19)
Central Asia	0.09(0.06 to 0.15)	0.19(0.11 to 0.29)		100.17		55.04(32.09 to 84.88)	56.48(33.31 to 86.52)		0.05(0.03 to 0.07)
Central Europe	0.46(0.28 to 0.69)	0.62(0.4 to 0.95)		36.37		72.07(43.89 to 107.99)	63.6(40.46 to 95.41)		-0.13(-0.23 to -0.02)
Central Latin America	0.34(0.22 to 0.52)	1.07(0.67 to 1.62)		210.20		92.71(58.07 to 140.25)	94.46(58.99 to 143.91)		0.13(0.10 to 0.16)
Central Sub-Saharan Africa	0.04(0.02 to 0.06)	0.1(0.06 to 0.15)		135.19		41.39(24.32 to 64.91)	42.28(25.35 to 66.35)		0.06(0.05 to 0.07)
East Asia	2.09(1.21 to 3.22)	4.84(2.85 to 7.37)		131.53		57.9(33.62 to 88.78)	46.78(27.91 to 70.91)		-0.25(-0.41 to -0.09)
Eastern Europe	1.16(0.71 to 1.76)	1.68(1.01 to 2.54)		44.85		123.66(75.76 to 185.98)	123.56(75.4 to 185.08)		0.02(0.01 to 0.04)
Eastern Sub-Saharan Africa	0.17(0.1 to 0.26)	0.35(0.21 to 0.55)		112.56		47.34(27.96 to 74.47)	48.07(28.63 to 75.84)		0.04(0.02 to 0.05)
High-income Asia Pacific	0.23(0.13 to 0.36)	0.48(0.29 to 0.76)		113.56		25.85(15.29 to 41.22)	23.39(14.17 to 37.35)		-0.13(-0.21 to -0.06)
High-income North America	0.54(0.34 to 0.82)	1.08(0.68 to 1.58)		100.53		36.25(23.09 to 54.54)	35.53(22.46 to 51.92)		0.20(0.13 to 0.27)
North Africa and Middle East	0.33(0.2 to 0.52)	0.94(0.56 to 1.46)		182.62		41.11(24.29 to 65.09)	42.84(25.5 to 67.53)		0.12(0.11 to 0.13)
Oceania	0.01(0.01 to 0.01)	0.02(0.01 to 0.04)		167.21		68.71(40.66 to 105.01)	71.31(43.08 to 107.33)		0.14(0.13 to 0.16)
South Asia	1.89(1.14 to 2.86)	5.17(3.11 to 7.94)		173.57		66.96(39.99 to 102.87)	73.7(44.3 to 113.02)		0.24(0.21 to 0.27)
Southeast Asia	1.03(0.62 to 1.58)	2.46(1.48 to 3.77)		138.21		93.43(56.28 to 141.89)	83.47(50.21 to 127.59)		-0.12(-0.21 to -0.02)
Southern Latin America	0.05(0.03 to 0.08)	0.11(0.07 to 0.17)		105.58		25.77(15.39 to 39.81)	27.98(16.88 to 43.59)		0.33(0.28 to 0.37)
Southern Sub-Saharan Africa	0.07(0.04 to 0.11)	0.15(0.09 to 0.22)		115.82		60.68(35.98 to 94.82)	62.47(37.46 to 96.99)		0.10(0.08 to 0.12)
Tropical Latin America	0.18(0.12 to 0.28)	0.43(0.27 to 0.65)		132.03		45.58(28.72 to 68.67)	37.17(23.56 to 56.27)		-0.31(-0.43 to -0.20)
Western Europe	1.08(0.67 to 1.65)	1.89(1.2 to 2.86)		75.2		43.51(27.09 to 66.8)	45.51(28.9 to 68.63)		0.21(0.18 to 0.24)
Western Sub-Saharan Africa	0.18(0.11 to 0.28)	0.38(0.22 to 0.59)		106.94		43.35(25.77 to 68.32)	44.09(25.96 to 69.48)		0.04(0.03 to 0.05)

DALYs: disability-adjusted life-years; SDI: socio-demographic index; EAPC: estimated annual percentage change; UI: uncertainty interval; CI: confidence interval

**Table 4:** Projections global incident cases and prevalent cases of benign prostatic hyperplasia from 2022 to 2035

Measure	Number of cases (95% UI), ×1000			
	1990	2021	2022	2035
**Incidence**				
Overall	6406.04(3834.37,9659.44)	13787.56(8500.62,20385.47)	13918.71(13205.53,14631.89)	18109.76(12621.20,23598.33)
40–59	1925.21(1059.41,3123.44)	3551.29(2029.81,5625.35)	3546.70(3362.21,3731.18)	4165.56(2846.42,5484.69)
60–79	4291.23(2697.40,6214.14)	9652.23(6220.89,13784.98)	9769.99(9272.07,10267.91)	12977.31(9096.93,16857.69)
80+	189.60(77.56,321.86)	584.04(249.92,975.14)	602.02(571.25,632.80)	966.90(677.84,1255.95)
**Prevalence**				
Overall	50673.34(34859.41,71557.18)	112311.74(79128.16,155262.40)	113942.78(108437.85,119447.71)	156343.38(111350.39,201336.37)
40–59	9371.60(6064.59,14263.30)	17231.06(11266.88,25839.80)	17258.92(16415.01,18102.84)	19947.18(13990.39,25903.97)
60–79	36741.03(25388.59,51343.58)	81268.58(57440.04,111633.19)	82408.51(78435.73,86381.29)	113499.71(81015.24,145984.18)
80+	4560.70(3406.23,5950.30)	13812.10(10421.25,17789.41)	14275.35(13587.11,14963.59)	22896.50(16344.76,29448.23)

UI: uncertainty interval

**Table 5:** Projections global crude rate for incidence and prevalence of benign prostatic hyperplasia from 2022 to 2035

Measure	Crude rate (95% UI), ×100,000			
	1990	2021	2022	2035
**Incidence**				
Overall	952.56(570.16,1436.33)	972.28(599.45,1437.56)	962.42(913.11,1011.74)	998.55(695.92,1301.19)
40–59	422.58(232.54,685.58)	386.95(221.17,612.94)	381.50(361.66,401.34)	387.05(264.48,509.61)
60–79	2162.05(1359.03,3130.87)	2180.78(1405.52,3114.52)	2137.38(2028.45,2246.31)	2043.10(1432.19,2654.02)
80+	1028.23(420.60,1745.45)	1012.30(433.18,1690.19)	1012.75(960.98,1064.53)	946.26(663.37,1229.14)
**Prevalence**				
Overall	7534.99(5183.50,10640.36)	7920.08(5580.01,10948.90)	7878.68(7498.04,8259.32)	8620.60(6139.74,11101.46)
40–59	2057.04(1331.16,3130.75)	1877.50(1227.64,2815.51)	1856.45(1765.68,1947.23)	1853.41(1299.93,2406.88)
60–79	18511.25(12791.55,25868.46)	18361.45(12977.74,25221.90)	18028.54(17159.41,18897.66)	17869.01(12754.76,22983.25)
80+	24732.69(18471.98,32268.49)	23940.14(18062.87,30833.92)	24014.66(22856.88,25172.45)	22407.79(15995.90,28819.69)

UI: uncertainty interval

### Regional incidence, prevalence, and DALYs

Between 1990 and 2021, the largest increases of incident cases (201.74%), prevalent cases (209.58%), and DALYs (210.2%) were Central Latin America (Tables 1, 2 and 3). In 2021, the regions with highest ASIR, ASPR, and ASDR were recorded in Eastern Europe. From 1990 to 2021, the largest increases of ASIR (EAPC = 0.47, 95% CI = 0.38 to 0.56) of BPH showed in High-income North America, and the largest increases of ASPR (EAPC = 0.33, 95% CI = 0.29 to 0.38) and ASDR (EAPC = 0.33, 95% CI = 0.28 to 0.37) of BPH showed in Southern Latin America. The most significant downward trend of ASIR (EAPC = -0.25, 95% CI =-0.36 to -0.14), ASPR (EAPC = -0.32, 95% CI =-0.43 to -0.20), and ASDR (EAPC = -0.31, 95% CI =-0.43 to -0.20) of BPH showed in Tropical Latin America.

### National incidence, prevalence, and DALYs

In 2021, the highest number of incident cases (3244.46*10^3^), prevalent cases (23111.20*10^3^), and DALYs (460.19*10^3^) were in China (Table S1-S3). Compared to 1990, United Arab Emirates had the largest increase in incident cases (1505.30%), prevalent cases (1381.24%), and DALYs (1384.54%) in 2021. The increase in ASIR was largest in Austria (EAPC = 0.72, 95% CI = 0.61 to 0.83), followed by Sri Lanka (EAPC = 0.63, 95% CI = 0.49 to 0.77) and Libya (EAPC = 0.60, 95% CI = 0.45 to 0.75). The increase in ASPR was largest in Austria (EAPC = 0.57, 95% CI = 0.45 to 0.69), followed by Sri Lanka (EAPC = 0.52, 95% CI = 0.38 to 0.67) and Libya (EAPC = 0.47, 95% CI = 0.42 to 0.52). The increase in ASDR was largest in Austria (EAPC = 0.58, 95% CI = 0.47 to 0.70), followed by Sri Lanka (EAPC = 0.53, 95% CI = 0.38 to 0.68) and Libya (EAPC = 0.49, 95% CI = 0.44 to 0.54).

**Fig. 1:**
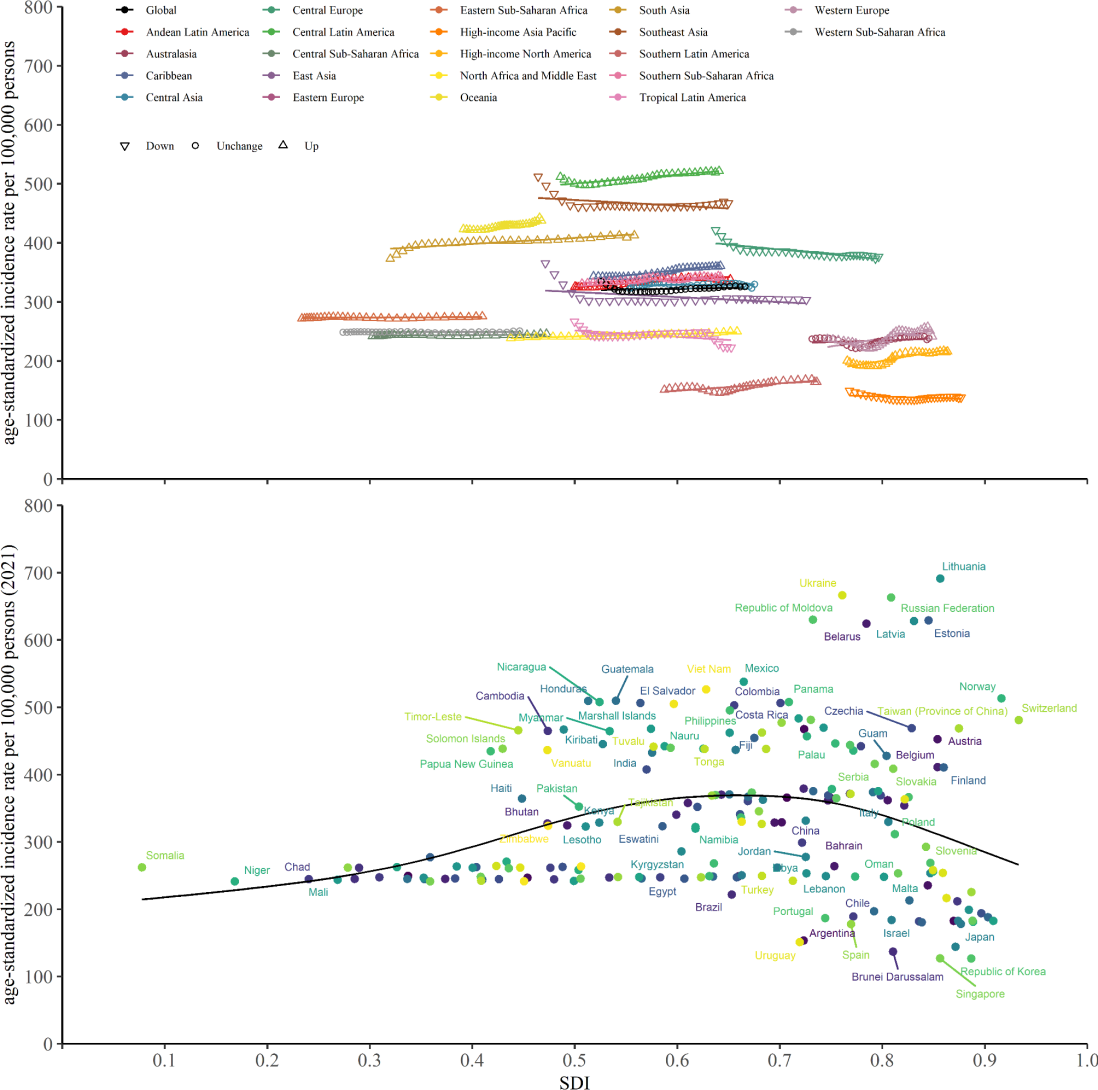
The ASIR of BPH for 21 regions (**a**) and 204 countries and territories (**b**) by SDI. **a**, 21 regions by SDI from 1990 to 2021. **b**, 204 countries and territories by SDI in 2021. ASIR, age-standardized incidence rate; BPH, benign prostatic hyperplasia

### Burden of BPH by SDI

The number of incident cases, prevalent cases, and DALYs were increased in all five SDI regions between 1990 and 2021 (Tables 1, 2 and 3). The middle SDI quintile level had the highest incident cases, prevalence cases, and DALYs of BPH in 2021. The ASIR of BPH decreased in the high-middle SDI region, and increased in the high, low-middle, and low SDI regions from 1990 to 2021 (Fig. 1a). We observed similar associations between ASPR and ASDR and the SDI level (Figure S1a and S2a). With the increase of SDI, the trends of ASIR, ASPR, and ASDR all exhibit an initial rise followed by a gradual decline. Among the 204 countries and territories, the ASIR, ASPR, and ASDR of BPH in Lithuania were much higher than the expected levels in 2021 (Fig. 1b, Figure S1b and S2b).

**Fig. 2:**
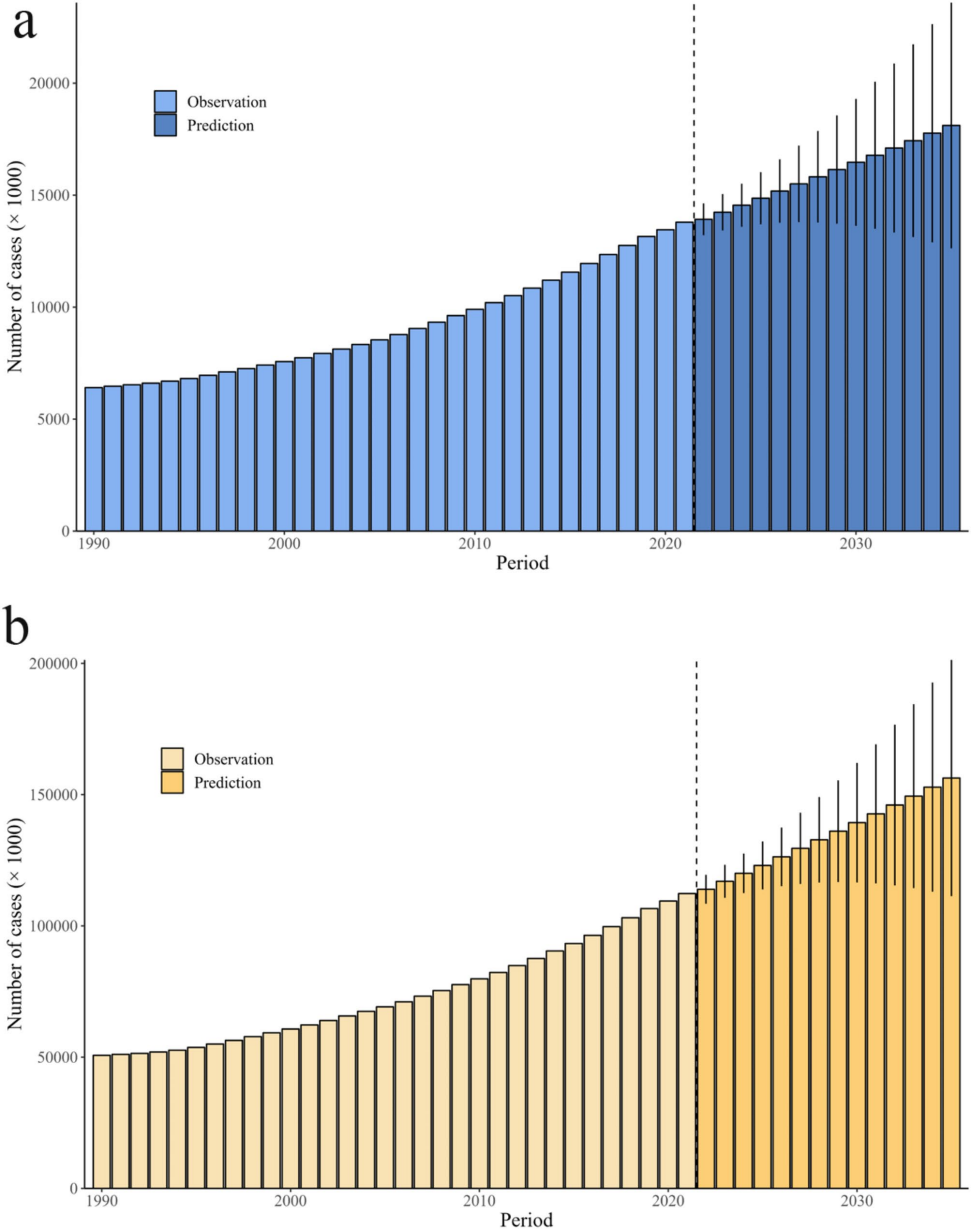
Projected trends for incident cases(**a**) and prevalent cases(**b**) of BPH globally from 2022 to 2035. BPH, benign prostatic hyperplasia

**Fig. 3:**
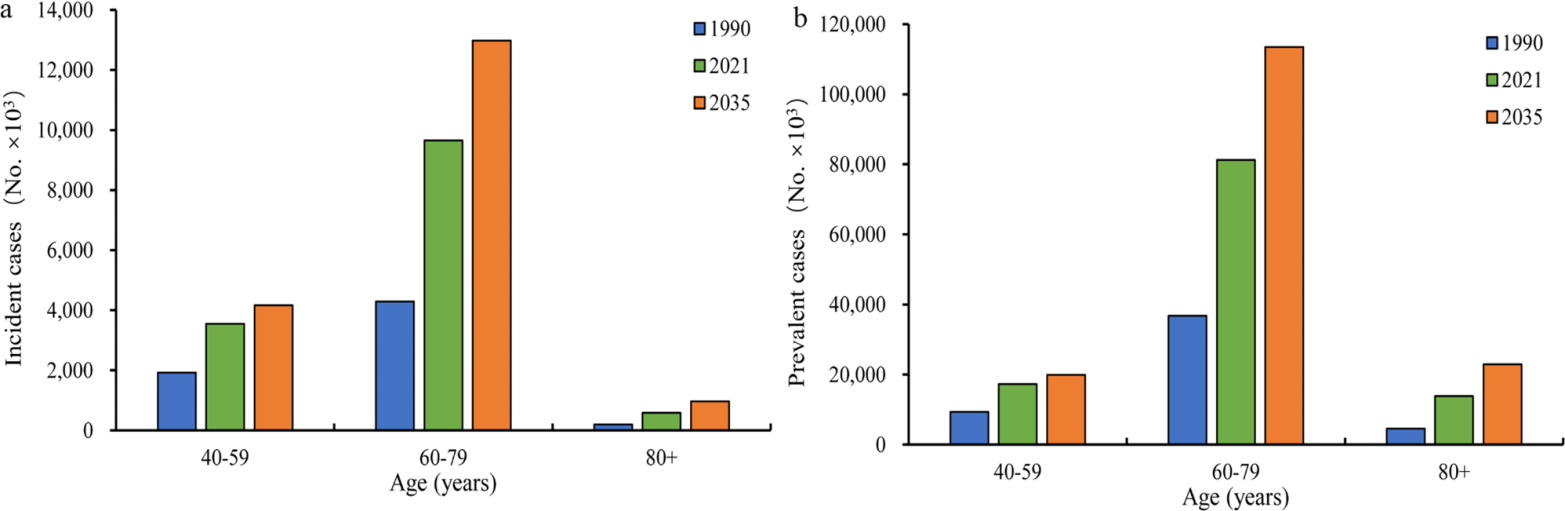
Global incident cases(**a**) and prevalent cases(**b**) of BPH by age between 1990 and 2035. BPH, benign prostatic hyperplasia

### Projections of BPH incidence and prevalence by 2035

Figure 2 displays the projected trends for incident cases and prevalent cases of BPH globally from 2022 to 2035. The global incident cases and prevalent cases are expected to increase from 139.19 to 1139.43 per 100,000 populations in 2022 to 181.10 and 1563.43 per 100,000 populations in 2035, respectively (Table 4). Similarly, the global crude rate of incidence and prevalence are expected to increase from 962.42 to 7878.68 per 100,000 populations in 2022 to 998.55 and 8620.60 per 100,000 populations in 2035, respectively (Table 5). In 2035, the incident cases and prevalent cases of BPH mainly concentrated in groups aged 60–79 years (Fig. 3).

## Discussion

We found that the number of incident cases, prevalent cases, and DALYs of BPH have been increasing from 1990 to 2021 worldwide. However, the ASIR, ASPR, and ASDR of BPH remained stable during the study period. Based on the results of the BAPC model projections, the increasing trend in incidence and prevalence of BPH is projected to continue until 2035. In GBD 21 regions, the largest increases of ASIR of BPH occurred in High-income North America, and the largest increases of ASPR and ASDR of BPH occurred in Southern Latin America between 1990 and 2021. In 2021, the regions with highest ASIR, ASPR, and ASDR were Eastern Europe. Nationally, Austria experienced the largest increases in ASIR, ASPR, and ASDR of BPH from 1990 to 2021. The middle SDI quintile level had the highest incident cases, prevalence cases, and DALYs of BPH in 2021. With the increase of SDI, the trends of ASIR, ASPR, and ASDR all exhibit an initial rise followed by a gradual decline. In addition, the estimated incidence and prevalence of BPH are projected to continue rising in 60–79 age group until 2035, emphasizing that BPH is becoming a worldwide and regional health threat. Our study utilizes the latest data to conduct a comprehensive analysis of the global burden of BPH, not only revealing its geographical distribution characteristics but also exploring its temporal variation patterns. More importantly, using the BAPC model, we predict the potential future trends of BPH, offering information support for disease prevention and control policy formulation, as well as healthcare resource allocation.

Our analysis revealed intriguing trends in the geographical distribution of BPH. While the overall prevalence and burden remained stable globally, there were notable regional variations. The middle SDI quintile with aging populations and improved healthcare access reported a slightly higher incidence of BPH, likely due to increased life expectancy [13, 20]. Moreover, in the low and low-middle SDI quintile, where access to healthcare services is limited, the true burden of BPH might be underestimated, as many cases go undiagnosed or underreported. In the high-middle and high SDI quintile, advancements in medical technology have led to the development of more effective treatments for BPH, including minimally invasive procedures and targeted therapies [21, 22]. These advancements have not only improved patient outcomes but also contributed to the stability of the ASIR, ASPR, and ASDR by reducing the severity and duration of symptoms, thereby decreasing the number of DALYs lost to the disease. It is crucial for policymakers and healthcare providers to continue monitoring the global burden of BPH, particularly considering population aging. Collaborative efforts across nations, focusing on improving access to healthcare and investing in research for novel treatments, will be essential to manage and potentially reduce the global impact of BPH in the coming decades.

Our study found that the highest incidence of BPH occurs at the age range of 60–79, while the age over 80 is the stage with the highest prevalence of BPH in 2035. This finding underscores a crucial period, specifically the age range of 60 to 79, when BPH is most likely to develop due to various biological and hormonal changes that men experience as they age. By 2035, the demographic shift towards an older population could lead to a significant increase in the number of BPH cases among individuals aged 80 and older, creating a heavy disease burden. It is important to note that the progression of BPH is often gradual and may not present with significant symptoms initially [2]. However, as men age, the enlargement of the prostate gland can compress the urethra, leading to a variety of urinary symptoms such as frequent urination, urgency, difficulty in starting urination, and a weak urine stream. These symptoms can significantly impact a man’s quality of life and may necessitate medical intervention [4, 6]. Therefore, targeted screening and early diagnosis programs could be implemented for men in their 50s and 60s, when the incidence of BPH is rapidly rising, to identify and manage the condition before it progresses to more severe stages. Moreover, research into the underlying mechanisms of BPH progression and the identification of modifiable risk factors could help in developing novel therapeutic approaches and reducing the burden of the disease [23–27]. Lifestyle modifications, such as maintaining a healthy diet, engaging in regular physical activity, and managing other comorbidities like diabetes and obesity, may also play a role in slowing down the progression of BPH [28–30]. Overall, the recognition of the age-specific patterns in BPH epidemiology highlights the need for age-appropriate prevention, screening, and management strategies to improve the health outcomes and quality of life of men affected by this common condition.

With the acceleration of global aging, the disease burden caused by BPH in middle-aged and elderly populations may become increasingly severe. This encompasses not only the direct burden caused by BPH but also the indirect burden associated with it. The direct burden of BPH primarily relates to the symptoms and complications arising from the enlarged prostate gland, such as urinary retention, recurrent urinary tract infections, bladder stones, and, in severe cases, renal dysfunction. These can significantly impair an individual’s quality of life, leading to decreased physical activity, social isolation, and emotional distress [4, 5]. Additionally, the treatment of BPH, including medications and surgical interventions, can impose financial costs on individuals and healthcare systems [31, 32]. However, the indirect burden of BPH is equally significant and often overlooked. It encompasses the impact on caregivers, families, and society [33]. For instance, the need for ongoing care and support for BPH patients can strain familial relationships and emotional resources, placing a heavy burden on caregivers. Furthermore, the inability to work or engage in daily activities due to BPH symptoms can lead to lost productivity and economic contributions from affected individuals. As the global population ageing, the prevalence of BPH is projected to rise, exacerbating the direct and indirect burdens associated with the condition [34]. Therefore, it is imperative to develop comprehensive strategies to address BPH, including prevention, early diagnosis, and effective management. This involves raising awareness about BPH and its symptoms, promoting healthy lifestyles to reduce modifiable risk factors, and improving access to timely and appropriate healthcare services.

The limitation in this study was the sparsity of data, particularly pronounced in low- and middle-income countries, potentially leading to an inadequate representation of genuine variations across geographical regions, gender, and temporal dimensions. Additionally, GBD 2021 study did not assign any cause-specific deaths to BPH, consequently, no YLLs were estimated for this condition. Furthermore, the lack of data on indirect burdens associated with BPH may prevent a complete reflection of the overall disease burden. Finally, the accuracy and robustness of the predictions depended on the accuracy of available morbidity and mortality data in the GBD database, the predicted figures should be interpreted with cautions.

## Conclusions

In conclusion, the persistent burden of BPH continues to pose a critical public health challenge. The escalating prevalence among middle-aged and elderly populations underscores the imperative to tackle this widespread condition. Formulating strategies that emphasize prevention, facilitate timely diagnosis, and ensure effective management can contribute to mitigating both the direct and indirect impacts of BPH, ultimately reducing the overall burden of the disease.

## Electronic supplementary material

Below is the link to the electronic supplementary material.

Supplementary Material 1

Supplementary Material 2

Supplementary Material 3

Supplementary Material 4

## Data Availability

The datasets generated during the current study are available in the Global Health Data Exchange query tool (https://vizhub.healthdata.org/gbd-results).
