# Supplementary material for: Global, regional, and national burden of benign prostatic hyperplasia from 1990 to 2021 and projection to 2035
Source: BMC Urol. 2025 Feb 19;25:34. doi: 10.1186/s12894-025-01715-9 (PMC11837592; doi:10.1186/s12894-025-01715-9)
Supplement: Supplementary file 1 — Supplementary Material 1 [file 12894_2025_1715_MOESM1_ESM.docx]

**Table S1** Incidence and age-standardized incidence rate of benign prostatic hyperplasia in 204 countries and territories between 1990 and 2021

| Location | All ages, No. ×10^3^ (95% UI) | |  | Change (%) |  | Age-standardized rate per 100,000, No. (95% UI) | |  | EAPC (95% CI) |
| --- | --- | --- | --- | --- | --- | --- | --- | --- | --- |
|  | **1990** | **2021** |  | **1990–2021** |  | **1990** | **2021** |  | **1990–2021** |
| Afghanistan | 9.33(6.98 to 12.19) | 10.3(7.97 to 13.18) |  | 10.39 |  | 238.42(180.56 to 306.99) | 249.79(191.53 to 322.76) |  | 0.14(0.13 to 0.15) |
| Albania | 3.61(2.74 to 4.67) | 8.14(6.03 to 10.39) |  | 125.34 |  | 348(264.32 to 441.9) | 365.95(277.4 to 461.68) |  | 0.03(-0.04 to 0.09) |
| Algeria | 14.96(11.16 to 19.31) | 47.21(35.63 to 61.99) |  | 215.56 |  | 236.49(178.28 to 302.59) | 248.8(189.05 to 326.81) |  | 0.12(0.11 to 0.14) |
| American Samoa | 0.05(0.04 to 0.06) | 0.12(0.09 to 0.15) |  | 136.58 |  | 441.86(342.34 to 556.62) | 467.67(362.23 to 585.41) |  | 0.19(0.16 to 0.21) |
| Andorra | 0.06(0.04 to 0.07) | 0.15(0.11 to 0.19) |  | 164.45 |  | 177.86(133.03 to 231.13) | 182.8(136.69 to 237.55) |  | 0.43(0.3 to 0.56) |
| Angola | 4.9(3.7 to 6.52) | 13.89(10.39 to 18.48) |  | 183.6 |  | 240.59(181.48 to 312.84) | 246.8(187.49 to 323.19) |  | 0.04(0.03 to 0.06) |
| Antigua and Barbuda | 0.08(0.06 to 0.1) | 0.2(0.15 to 0.26) |  | 154.75 |  | 343.8(260.32 to 438.21) | 364.77(274.7 to 471.41) |  | 0.23(0.21 to 0.26) |
| Argentina | 21.67(16.38 to 28.29) | 38.44(28.82 to 50.32) |  | 77.37 |  | 144.33(110.7 to 187.04) | 153.54(115.76 to 200.55) |  | 0.3(0.2 to 0.41) |
| Armenia | 3.9(2.89 to 5.14) | 6.41(4.78 to 8.43) |  | 64.46 |  | 323.27(242.27 to 413.02) | 329.2(248.9 to 421.54) |  | 0.04(0 to 0.07) |
| Australia | 21.2(15.49 to 28.64) | 49.11(35.83 to 65.75) |  | 131.62 |  | 226.23(166.91 to 302.57) | 235.42(172.96 to 311.27) |  | 0.19(0.1 to 0.27) |
| Austria | 16.81(12.35 to 21.45) | 34.29(29.27 to 39.71) |  | 103.97 |  | 364.2(267.44 to 464.29) | 452.55(388.7 to 519.66) |  | 0.72(0.61 to 0.83) |
| Azerbaijan | 6.75(4.96 to 8.92) | 16.48(12.26 to 21.81) |  | 144.28 |  | 322.88(242.39 to 414.68) | 328.71(248.02 to 421.63) |  | 0.05(0.02 to 0.07) |
| Bahamas | 0.23(0.17 to 0.3) | 0.71(0.54 to 0.94) |  | 209.38 |  | 344.23(256.7 to 449.41) | 362.04(274.59 to 474.16) |  | 0.2(0.18 to 0.21) |
| Bahrain | 0.25(0.19 to 0.33) | 1.8(1.35 to 2.37) |  | 613.22 |  | 245.08(184.3 to 314.2) | 264.04(199.85 to 339.36) |  | 0.12(0.11 to 0.12) |
| Bangladesh | 81.03(62.18 to 103.07) | 238.49(181.83 to 303.15) |  | 194.33 |  | 313.77(238.19 to 401.03) | 324.68(248.7 to 411.25) |  | 0.2(0.18 to 0.23) |
| Barbados | 0.4(0.3 to 0.52) | 0.89(0.67 to 1.15) |  | 121.48 |  | 344.18(258.6 to 443.22) | 361.73(272.83 to 462.12) |  | -0.03(-0.05 to 0) |
| Belarus | 32.16(25 to 40.76) | 41.92(32.22 to 53.31) |  | 30.34 |  | 625.1(497.43 to 769.69) | 624.46(491.34 to 773.47) |  | 0.44(0.29 to 0.58) |
| Belgium | 27.19(17.37 to 34.71) | 40.79(27.38 to 51.13) |  | 50.04 |  | 398.01(257.83 to 505.26) | 411.25(276.04 to 515.86) |  | 0.24(0.21 to 0.27) |
| Belize | 0.16(0.12 to 0.2) | 0.55(0.42 to 0.72) |  | 251.77 |  | 337.8(253.52 to 432.72) | 357.83(270.79 to 463.87) |  | 0.05(0.03 to 0.06) |
| Benin | 2.3(1.76 to 3.01) | 6.13(4.63 to 7.95) |  | 166.37 |  | 239.94(183.63 to 312.85) | 245.25(185.66 to 316.7) |  | 0.19(0.17 to 0.21) |
| Bermuda | 0.1(0.07 to 0.12) | 0.22(0.16 to 0.28) |  | 128.27 |  | 337.99(252.69 to 437.81) | 354.1(266.51 to 462.3) |  | 0.13(0.12 to 0.14) |
| Bhutan | 0.4(0.3 to 0.53) | 1.02(0.79 to 1.3) |  | 153.56 |  | 313.54(237.45 to 402) | 327.43(253.15 to 418.49) |  | 0.18(0.17 to 0.2) |
| Bolivia (Plurinational State of) | 4.91(3.69 to 6.37) | 15.38(11.42 to 19.73) |  | 213.01 |  | 321.26(240 to 413.03) | 340.54(251.54 to 433.12) |  | 0.12(0.05 to 0.18) |
| Bosnia and Herzegovina | 6.45(4.9 to 8.35) | 10.97(8.17 to 14.16) |  | 70.04 |  | 352.59(268.12 to 453.22) | 379.12(286.32 to 483.7) |  | 0.09(0.08 to 0.11) |
| Botswana | 0.99(0.76 to 1.26) | 2.63(2.03 to 3.3) |  | 166.78 |  | 360.96(282.56 to 452.88) | 370.26(293.34 to 465.27) |  | -0.26(-0.37 to -0.14) |
| Brazil | 115.16(94.1 to 140.51) | 261.67(215.59 to 322.86) |  | 127.23 |  | 266.86(217.87 to 325.77) | 221.78(182.51 to 272.22) |  | 0.16(0.1 to 0.21) |
| Brunei Darussalam | 0.07(0.05 to 0.09) | 0.26(0.19 to 0.35) |  | 285.79 |  | 131.19(95.42 to 176.35) | 137.05(100.24 to 181.19) |  | 0.04(-0.03 to 0.11) |
| Bulgaria | 21.83(16.38 to 27.95) | 23.08(17.53 to 29.15) |  | 5.7 |  | 351.21(268.13 to 443.72) | 371.6(285.5 to 471.16) |  | 0.04(0.03 to 0.04) |
| Burkina Faso | 5.25(3.98 to 6.98) | 11.09(8.43 to 14.4) |  | 111.28 |  | 240.55(184.06 to 313.51) | 245.04(187.01 to 320.26) |  | -0.03(-0.04 to -0.01) |
| Burundi | 2.78(2.13 to 3.65) | 7.02(5.26 to 9.17) |  | 152.45 |  | 261.48(200.66 to 341.92) | 261.56(197.81 to 337.85) |  | 0.07(0.05 to 0.09) |
| Cabo Verde | 0.23(0.17 to 0.29) | 0.5(0.38 to 0.65) |  | 117.03 |  | 239.75(180.91 to 302.78) | 247.14(188.61 to 314.93) |  | 0.04(0.04 to 0.05) |
| Cambodia | 8.95(6.84 to 11.42) | 25.7(19.57 to 32.53) |  | 187.05 |  | 451.95(349.22 to 572.8) | 465.07(354.39 to 581.9) |  | 0.31(0.18 to 0.43) |
| Cameroon | 5.44(4.1 to 7.09) | 15.58(11.72 to 20.19) |  | 186.43 |  | 239.44(180.07 to 310.1) | 244.53(184.03 to 315.66) |  | 0.03(0.02 to 0.05) |
| Canada | 29.51(22.23 to 37.85) | 68.98(52.16 to 89.68) |  | 133.76 |  | 201.61(152.53 to 258.46) | 211.96(159.77 to 272.55) |  | 0.03(0.01 to 0.04) |
| Central African Republic | 1.36(1 to 1.8) | 2.67(2 to 3.57) |  | 95.93 |  | 242.06(182.32 to 316.01) | 247.59(189.59 to 320.97) |  | 0.33(0.23 to 0.43) |
| Chad | 3.36(2.49 to 4.41) | 7.96(6.08 to 10.49) |  | 137.12 |  | 240.41(180.17 to 313) | 244.8(186.46 to 318.67) |  | -0.21(-0.36 to -0.06) |
| Chile | 8.23(6.18 to 10.6) | 22.67(16.97 to 29.23) |  | 175.57 |  | 176.71(132.85 to 226.91) | 189.16(143.04 to 241.95) |  | 0.17(0.15 to 0.18) |
| China | 1460.43(1081.23 to 1887.29) | 3244.46(2482.94 to 4059.87) |  | 122.16 |  | 363.07(274.34 to 462.07) | 299.14(233.33 to 375.2) |  | 0.2(0.18 to 0.23) |
| Colombia | 41.88(32.16 to 53.58) | 128.48(97.43 to 164.68) |  | 206.74 |  | 484.87(371.69 to 613.74) | 503.02(381.71 to 640.16) |  | -0.02(-0.03 to 0) |
| Comoros | 0.27(0.2 to 0.35) | 0.61(0.46 to 0.78) |  | 130.04 |  | 261.25(196.71 to 335.64) | 261.5(197.72 to 336.34) |  | 0.02(0.01 to 0.04) |
| Congo | 1.2(0.88 to 1.58) | 3.48(2.61 to 4.59) |  | 190.86 |  | 243.1(184.18 to 314.16) | 247.67(185.88 to 318.62) |  | 0.13(0.11 to 0.15) |
| Cook Islands | 0.03(0.02 to 0.04) | 0.06(0.04 to 0.07) |  | 105.51 |  | 426.63(324.81 to 539.8) | 442.31(338.23 to 557.99) |  | 0.21(0.19 to 0.22) |
| Costa Rica | 4.02(3.08 to 5.12) | 13.2(10.07 to 17.11) |  | 227.92 |  | 482.08(368.12 to 612.86) | 506.47(388.99 to 652.14) |  | -0.04(-0.09 to 0) |
| Croatia | 10.55(8.88 to 12.35) | 14.45(11.6 to 17.41) |  | 36.96 |  | 389.16(324.89 to 453.43) | 369.3(302.12 to 441.8) |  | 0.19(0.17 to 0.22) |
| Cuba | 17.35(13.03 to 22.45) | 33.25(25.3 to 42.95) |  | 91.72 |  | 344.41(259.53 to 449.94) | 360.58(271.02 to 464.88) |  | 0.25(0.14 to 0.35) |
| Cyprus | 0.75(0.58 to 0.93) | 1.82(1.43 to 2.23) |  | 144.46 |  | 181.83(143.58 to 225.47) | 182.19(145.52 to 219.85) |  | 0.14(0.09 to 0.2) |
| Czechia | 25.63(19.94 to 31.45) | 44.23(34.92 to 53.92) |  | 72.54 |  | 431.52(339.44 to 524.78) | 469.18(373.59 to 570.87) |  | 0.08(0.07 to 0.09) |
| Cote d'Ivoire | 5.41(4.1 to 7.26) | 15.14(11.44 to 19.4) |  | 179.97 |  | 239.94(183.63 to 312.85) | 244.69(184.11 to 311.79) |  | 0.03(0.01 to 0.04) |
| Democratic People's Republic of Korea | 22.84(17.25 to 29.44) | 54.79(41.6 to 70.01) |  | 139.95 |  | 399.74(304.33 to 516.11) | 407.58(310.02 to 511.61) |  | 0.47(0.31 to 0.63) |
| Democratic Republic of the Congo | 18.48(13.82 to 24.36) | 44.13(32.72 to 58.32) |  | 138.74 |  | 242(182.72 to 315.46) | 245.93(185.86 to 321.47) |  | 0(-0.01 to 0.02) |
| Denmark | 6.55(4.58 to 9.17) | 10.11(7.07 to 13.86) |  | 54.31 |  | 189.73(134.19 to 265.94) | 193.95(136.21 to 267.37) |  | 0.28(0.25 to 0.31) |
| Djibouti | 0.19(0.14 to 0.25) | 0.99(0.74 to 1.28) |  | 421.48 |  | 260.25(196.22 to 333.33) | 263.14(198.22 to 337.96) |  | 0.22(0.19 to 0.25) |
| Dominica | 0.08(0.06 to 0.11) | 0.17(0.13 to 0.22) |  | 106.51 |  | 344.03(259.52 to 445.79) | 369.18(282.86 to 472.51) |  | 0.08(0 to 0.17) |
| Dominican Republic | 6.18(4.68 to 8.03) | 16.98(12.96 to 21.6) |  | 174.97 |  | 334.92(251.39 to 432.6) | 352.23(268.54 to 447.84) |  | 0.12(0.11 to 0.14) |
| Ecuador | 8.94(6.84 to 11.63) | 27.02(20.22 to 34.71) |  | 202.17 |  | 345.58(264.08 to 447.66) | 341.29(255.14 to 437.7) |  | 0.23(0.21 to 0.25) |
| Egypt | 33.6(25.38 to 43.81) | 90.03(67.18 to 119.65) |  | 167.99 |  | 233.92(178.6 to 303.05) | 245.64(182.79 to 322.39) |  | 0.04(0.03 to 0.06) |
| El Salvador | 6.56(5.02 to 8.38) | 12.6(9.71 to 15.94) |  | 92.01 |  | 478.65(366.12 to 610.42) | 506.41(388.62 to 642.49) |  | 0(-0.02 to 0.01) |
| Equatorial Guinea | 0.22(0.16 to 0.29) | 0.55(0.42 to 0.71) |  | 148.68 |  | 241.31(182.28 to 312.74) | 247.59(189.59 to 320.97) |  | 0(-0.03 to 0.03) |
| Eritrea | 1.32(0.99 to 1.74) | 3.16(2.36 to 4.12) |  | 139.7 |  | 259.78(198.11 to 336.06) | 262.59(196.99 to 338.01) |  | -0.03(-0.05 to -0.01) |
| Estonia | 4.97(3.88 to 6.18) | 6.34(4.96 to 7.81) |  | 27.56 |  | 626.47(496.26 to 771.68) | 629.13(496.31 to 775.61) |  | 0.17(0.15 to 0.2) |
| Eswatini | 0.42(0.32 to 0.56) | 0.79(0.59 to 1.03) |  | 85.21 |  | 313.76(237.57 to 402.77) | 323.47(246.11 to 421.23) |  | 0.17(0.16 to 0.18) |
| Ethiopia | 30.53(23.09 to 40.27) | 61.38(46.8 to 77.88) |  | 101.05 |  | 277.63(211.97 to 354.12) | 277.43(210.22 to 356.52) |  | 0.34(0.23 to 0.45) |
| Fiji | 0.73(0.56 to 0.92) | 1.71(1.3 to 2.2) |  | 134.83 |  | 431.97(330.53 to 545.12) | 454.7(347.89 to 568.27) |  | 0.4(0.27 to 0.53) |
| Finland | 12.02(10.1 to 14.05) | 22.04(18.53 to 25.72) |  | 83.39 |  | 404.83(341.23 to 472.27) | 410.87(349.58 to 473.31) |  | 0.06(0.04 to 0.07) |
| France | 61.89(45.61 to 82.04) | 102.03(74.93 to 136.39) |  | 64.84 |  | 176.92(131.04 to 234.14) | 180.74(134.76 to 238.24) |  | 0.12(0.06 to 0.18) |
| Gabon | 0.65(0.49 to 0.85) | 1.36(1 to 1.81) |  | 109.76 |  | 242.36(185.99 to 312.83) | 248.62(188.88 to 321.95) |  | 0.47(0.34 to 0.61) |
| Gambia | 0.47(0.35 to 0.61) | 1.18(0.89 to 1.53) |  | 153.01 |  | 239.67(181.26 to 309.44) | 243.8(183.8 to 318.29) |  | 0.05(0.04 to 0.06) |
| Georgia | 8.97(6.77 to 11.62) | 9.5(7.86 to 11.29) |  | 5.9 |  | 345.7(265.66 to 438.78) | 375.49(313.41 to 442.18) |  | 0.38(0.24 to 0.53) |
| Germany | 91.74(67.63 to 118.85) | 150.39(111.51 to 197.67) |  | 63.94 |  | 181.07(133.77 to 234.18) | 188.35(140.05 to 245.16) |  | 0.35(0.23 to 0.47) |
| Ghana | 7.56(5.67 to 9.84) | 18.97(14.44 to 24.65) |  | 150.78 |  | 241.26(181.66 to 311.08) | 245.77(185.7 to 314.63) |  | 0.24(0.22 to 0.27) |
| Greece | 12.84(9.49 to 16.89) | 18.13(14.08 to 22.64) |  | 41.18 |  | 178.14(131.56 to 233.82) | 197.11(153.86 to 245.16) |  | 0.08(0.06 to 0.1) |
| Greenland | 0.04(0.03 to 0.05) | 0.09(0.07 to 0.12) |  | 144.27 |  | 200(151.64 to 253.29) | 213.37(160.97 to 270.29) |  | 0.24(0.22 to 0.27) |
| Grenada | 0.1(0.08 to 0.13) | 0.22(0.17 to 0.29) |  | 122.52 |  | 344.69(262.13 to 440.29) | 367.32(276.82 to 471.84) |  | 0.03(0.02 to 0.05) |
| Guam | 0.16(0.12 to 0.21) | 0.45(0.34 to 0.58) |  | 172.21 |  | 418.35(323.19 to 524.33) | 427.8(326.72 to 539.64) |  | 0.05(0.04 to 0.06) |
| Guatemala | 8.74(6.56 to 11.29) | 26.46(20.49 to 33.93) |  | 202.81 |  | 482.17(363.87 to 613.34) | 509.92(393.47 to 649.52) |  | 0.26(0.23 to 0.29) |
| Guinea | 4.12(3.1 to 5.38) | 7.19(5.47 to 9.41) |  | 74.42 |  | 239.89(179.82 to 310.65) | 245.18(187.75 to 319.91) |  | 0.24(0.22 to 0.26) |
| Guinea-Bissau | 0.48(0.36 to 0.62) | 0.85(0.64 to 1.1) |  | 76.42 |  | 239.75(180.91 to 302.78) | 244.77(186.9 to 313.66) |  | 0.21(0.19 to 0.23) |
| Guyana | 0.64(0.48 to 0.81) | 1.2(0.91 to 1.58) |  | 88.99 |  | 348.08(263.19 to 441.42) | 370.57(281.06 to 473.26) |  | 0.06(-0.01 to 0.13) |
| Haiti | 5.74(4.34 to 7.44) | 12.84(9.67 to 16.66) |  | 123.71 |  | 344.61(265.36 to 443.42) | 364.36(274.15 to 465.92) |  | 0.48(0.33 to 0.63) |
| Honduras | 4.9(3.75 to 6.24) | 15.6(11.99 to 19.96) |  | 218.65 |  | 484.88(369.2 to 618.27) | 509.7(392.14 to 645.45) |  | 0.22(0.17 to 0.27) |
| Hungary | 22.63(17.18 to 29.03) | 30.49(23.06 to 38.72) |  | 34.75 |  | 354.29(272.61 to 448.45) | 374.11(286.38 to 469.09) |  | -0.36(-0.57 to -0.15) |
| Iceland | 0.23(0.17 to 0.3) | 0.49(0.36 to 0.67) |  | 115.78 |  | 173.29(128.09 to 225.8) | 178.35(130.11 to 237.45) |  | 0.09(0.07 to 0.11) |
| India | 973.19(741.95 to 1239.13) | 2620.43(2015.18 to 3300.15) |  | 169.26 |  | 385.65(298 to 484.41) | 432.83(335.69 to 540.28) |  | 0.46(0.33 to 0.6) |
| Indonesia | 289.82(228.59 to 358.64) | 561.94(442.35 to 702.87) |  | 93.89 |  | 579.86(464.03 to 701.93) | 437.02(346.04 to 538.53) |  | 0.11(0.1 to 0.13) |
| Iran (Islamic Republic of) | 37.82(28.3 to 50.48) | 103.52(78.94 to 132.52) |  | 173.71 |  | 251.18(191.03 to 322.44) | 261.83(198.95 to 333.81) |  | 0.44(0.31 to 0.57) |
| Iraq | 9.08(6.89 to 11.7) | 31.15(23.5 to 41.24) |  | 243.13 |  | 239.81(182.08 to 311.79) | 250.6(190.23 to 325.46) |  | 0.07(-0.05 to 0.19) |
| Ireland | 3.36(2.5 to 4.47) | 6.78(5.03 to 8.93) |  | 102 |  | 176.18(131.09 to 232) | 182.73(136.12 to 239.48) |  | 0.26(0.24 to 0.29) |
| Israel | 3.9(2.9 to 5.19) | 10.12(7.38 to 13.21) |  | 159.23 |  | 178.33(133.4 to 235.95) | 184.1(136.52 to 242.62) |  | -0.03(-0.14 to 0.08) |
| Italy | 135.57(109.64 to 166.95) | 178.78(148.58 to 211.8) |  | 31.88 |  | 346.64(283.36 to 423.7) | 329.75(277.78 to 387.41) |  | 0.21(0.13 to 0.3) |
| Jamaica | 2.79(2.1 to 3.57) | 5.48(4.12 to 7.12) |  | 96.8 |  | 340.42(256.34 to 434.07) | 362.85(271.79 to 469.57) |  | 0.04(0.01 to 0.07) |
| Japan | 124.25(94.49 to 162) | 186.05(144.89 to 239.69) |  | 49.74 |  | 155(117.63 to 202.38) | 144.23(112.72 to 184.91) |  | -0.01(-0.02 to 0) |
| Jordan | 1.7(1.26 to 2.22) | 11.78(8.99 to 14.99) |  | 594.62 |  | 239.81(181.17 to 305.11) | 277.6(213.79 to 349.32) |  | 0.1(0.08 to 0.11) |
| Kazakhstan | 15.8(11.89 to 20.98) | 26.3(19.88 to 34.89) |  | 66.48 |  | 323.75(248.15 to 413.26) | 331.55(255.66 to 424.62) |  | 0.14(0.12 to 0.15) |
| Kenya | 13.35(10.17 to 17.03) | 37.6(28.58 to 48.6) |  | 181.7 |  | 326.73(249.38 to 415.57) | 328.72(250.56 to 417.63) |  | 0.11(0.08 to 0.13) |
| Kiribati | 0.07(0.05 to 0.09) | 0.14(0.1 to 0.17) |  | 99.63 |  | 428.08(325.93 to 538.32) | 445.44(339.81 to 550.96) |  | -0.02(-0.06 to 0.02) |
| Kuwait | 0.94(0.71 to 1.24) | 4.64(3.51 to 6.11) |  | 394.12 |  | 241.59(182.96 to 312.76) | 253.59(191.31 to 331.35) |  | 0.08(0.06 to 0.09) |
| Kyrgyzstan | 3.38(2.59 to 4.37) | 6.25(4.74 to 8.17) |  | 84.8 |  | 284.9(215.69 to 363.55) | 286.14(219.07 to 359.68) |  | 0.01(-0.02 to 0.03) |
| Lao People's Democratic Republic | 4.73(3.61 to 6.07) | 10.91(8.26 to 13.81) |  | 130.34 |  | 455.5(350.64 to 569.27) | 466.92(357.45 to 582.91) |  | 0.11(0.1 to 0.12) |
| Latvia | 8.56(6.81 to 10.53) | 9.14(7.15 to 11.29) |  | 6.71 |  | 626.35(492.6 to 764.38) | 628.13(492.56 to 769.09) |  | 0.09(0.08 to 0.11) |
| Lebanon | 2.7(2 to 3.61) | 6.45(4.94 to 8.22) |  | 139.42 |  | 238.47(178.69 to 312.62) | 249.34(189.36 to 323.06) |  | 0.06(0.05 to 0.07) |
| Lesotho | 1.01(0.76 to 1.31) | 1.45(1.1 to 1.91) |  | 44.35 |  | 311.74(237.67 to 399.53) | 322.93(246.33 to 413.5) |  | 0.16(0.15 to 0.17) |
| Liberia | 1.55(1.16 to 2.04) | 2.78(2.08 to 3.58) |  | 78.93 |  | 241.87(182.36 to 315) | 246.76(184.88 to 321.75) |  | 0(-0.03 to 0.03) |
| Libya | 2.42(1.81 to 3.12) | 6.85(5.16 to 8.77) |  | 183.23 |  | 238.87(179.71 to 308.78) | 253.24(188.98 to 329.92) |  | 0.6(0.45 to 0.75) |
| Lithuania | 12.49(10.48 to 14.45) | 14.86(12.42 to 17.47) |  | 18.93 |  | 687.4(573.45 to 803.31) | 691.36(580.11 to 813.78) |  | -0.01(-0.03 to 0) |
| Luxembourg | 0.44(0.33 to 0.58) | 1.01(0.76 to 1.33) |  | 127.83 |  | 187.03(141.6 to 242.95) | 199.33(150.45 to 262.57) |  | 0.01(0 to 0.03) |
| Madagascar | 6.91(5.17 to 9.01) | 15.16(11.27 to 20.07) |  | 119.4 |  | 260.5(195.85 to 335.37) | 261.47(199.13 to 342.33) |  | 0.04(0.03 to 0.06) |
| Malawi | 4.91(3.72 to 6.43) | 9.06(6.84 to 11.69) |  | 84.62 |  | 261.62(199.26 to 337.65) | 263.67(198.21 to 341.07) |  | 0.03(0.02 to 0.04) |
| Malaysia | 20.36(15.84 to 25.59) | 69.74(53.46 to 89.34) |  | 242.54 |  | 459.71(356.83 to 579.62) | 469.78(362.84 to 596.2) |  | 0.02(0.01 to 0.03) |
| Maldives | 0.25(0.19 to 0.33) | 0.86(0.66 to 1.08) |  | 239.59 |  | 454.06(347.5 to 572.59) | 462.14(352.21 to 586.6) |  | 0.34(0.22 to 0.46) |
| Mali | 5.11(3.84 to 6.77) | 11.57(8.71 to 15.1) |  | 126.4 |  | 240.64(180.81 to 315.77) | 243.68(183.65 to 315.76) |  | 0.21(0.19 to 0.23) |
| Malta | 0.48(0.38 to 0.58) | 1.06(0.83 to 1.28) |  | 119.09 |  | 243.97(195.11 to 290.53) | 248.18(196.82 to 296.62) |  | 0(-0.01 to 0.02) |
| Marshall Islands | 0.03(0.03 to 0.04) | 0.08(0.06 to 0.11) |  | 147.19 |  | 438.49(337.36 to 555.29) | 468.25(364.1 to 584.86) |  | 0.15(0.13 to 0.17) |
| Mauritania | 1.18(0.88 to 1.52) | 2.69(2.04 to 3.51) |  | 128.69 |  | 239.21(180.98 to 307.52) | 242.24(182.6 to 314.74) |  | 0.11(0.07 to 0.16) |
| Mauritius | 1.62(1.2 to 2.06) | 4.52(3.45 to 5.72) |  | 179.82 |  | 462.8(352.61 to 576.47) | 483.59(375.08 to 601.36) |  | 0.01(-0.02 to 0.05) |
| Mexico | 110.82(90.69 to 133.13) | 328.14(269.5 to 393.15) |  | 196.11 |  | 539.11(438.62 to 648.34) | 538.1(441.38 to 644.13) |  | 0.06(-0.01 to 0.13) |
| Micronesia (Federated States of) | 0.1(0.08 to 0.13) | 0.16(0.12 to 0.21) |  | 58.49 |  | 422.7(326.59 to 531.22) | 442.38(338.26 to 559.16) |  | 0.13(0.12 to 0.14) |
| Monaco | 0.05(0.04 to 0.07) | 0.07(0.06 to 0.1) |  | 50.52 |  | 177.2(130.92 to 235.12) | 182.8(136.69 to 237.55) |  | 0.02(0 to 0.03) |
| Mongolia | 1.54(1.18 to 1.97) | 3.28(2.48 to 4.24) |  | 112.93 |  | 316.66(242.77 to 401.04) | 320.11(243.23 to 409.54) |  | 0.05(0.04 to 0.06) |
| Montenegro | 0.99(0.74 to 1.26) | 1.79(1.36 to 2.28) |  | 80.94 |  | 355.02(267.87 to 451.12) | 375.61(287.95 to 467.88) |  | 0.06(0.04 to 0.07) |
| Morocco | 16.95(12.6 to 22.06) | 44.9(33.15 to 58.97) |  | 164.88 |  | 235.55(174.89 to 306.09) | 247.85(185.36 to 322.79) |  | 0.04(-0.03 to 0.11) |
| Mozambique | 7.82(5.93 to 10.18) | 13.72(10.35 to 17.78) |  | 75.51 |  | 260.43(199.23 to 338.45) | 262.67(200.07 to 339.34) |  | 0.39(0.25 to 0.52) |
| Myanmar | 51.73(39.57 to 65.6) | 104.61(78.85 to 134.63) |  | 102.21 |  | 456.61(350.75 to 571.66) | 464.49(358.89 to 581.7) |  | -0.27(-0.42 to -0.11) |
| Namibia | 0.99(0.74 to 1.32) | 2.01(1.51 to 2.62) |  | 102.84 |  | 312.69(237.77 to 407.68) | 322.36(241.77 to 413.61) |  | 0.21(0.19 to 0.23) |
| Nauru | 0.01(0.01 to 0.01) | 0.01(0.01 to 0.01) |  | 1.69 |  | 421.71(323.31 to 534.26) | 438.7(336.64 to 553.73) |  | 0.02(0.01 to 0.03) |
| Nepal | 14.38(11.15 to 18.29) | 31.53(25.15 to 39.16) |  | 119.28 |  | 282.53(222.21 to 356.88) | 271.03(217.85 to 334.97) |  | -0.01(-0.03 to 0) |
| Netherlands | 15.6(11.52 to 20.44) | 29.29(21.44 to 38.69) |  | 87.74 |  | 178.34(132.22 to 234.41) | 181.43(134.05 to 235.56) |  | 0.11(0.1 to 0.12) |
| New Zealand | 5.47(4.3 to 6.79) | 10.34(8.47 to 12.64) |  | 89.17 |  | 294.29(234.02 to 363.03) | 256.11(211.05 to 310.69) |  | 0.21(0.12 to 0.3) |
| Nicaragua | 3.53(2.67 to 4.57) | 11.4(8.81 to 14.61) |  | 223.18 |  | 483.64(367.48 to 614.75) | 507.94(390.64 to 644.98) |  | 0.1(0.07 to 0.12) |
| Niger | 3.66(2.74 to 4.75) | 10.15(7.61 to 13.08) |  | 177.17 |  | 238.77(180.81 to 309.06) | 241.27(182.41 to 312.81) |  | 0.15(0.15 to 0.16) |
| Nigeria | 62.34(47.07 to 80.64) | 110.95(84.35 to 141.46) |  | 77.97 |  | 256.31(194.78 to 329.8) | 258.43(196.35 to 330.65) |  | 0.13(0.12 to 0.14) |
| Niue | 0(0 to 0.01) | 0(0 to 0.01) |  | 16.84 |  | 432.85(333.09 to 539.89) | 456.96(350.54 to 567.01) |  | 0.2(0.19 to 0.22) |
| North Macedonia | 3.26(2.47 to 4.22) | 6.6(4.97 to 8.47) |  | 102.77 |  | 354.23(268.11 to 454.03) | 378.5(288.51 to 476.09) |  | 0.11(0.09 to 0.12) |
| Northern Mariana Islands | 0.04(0.03 to 0.05) | 0.13(0.09 to 0.16) |  | 232.84 |  | 422.39(323.6 to 535.79) | 435.89(335.22 to 546.49) |  | 0.14(0.12 to 0.16) |
| Norway | 14.48(11.44 to 17.77) | 23.03(18.41 to 28.25) |  | 59.08 |  | 516.83(413.9 to 633.61) | 513.23(411.3 to 626.14) |  | 0.22(0.19 to 0.24) |
| Oman | 0.9(0.67 to 1.14) | 3.07(2.31 to 3.97) |  | 242.96 |  | 237.79(181.53 to 303.5) | 248.64(186.87 to 321.28) |  | 0.02(0 to 0.04) |
| Pakistan | 107.81(83.21 to 138.07) | 234.11(179.72 to 297.84) |  | 117.15 |  | 337.75(259.56 to 429.94) | 352.5(271.61 to 448.28) |  | 0.21(0.19 to 0.24) |
| Palau | 0.02(0.02 to 0.03) | 0.06(0.04 to 0.07) |  | 164.63 |  | 425.89(325.66 to 531.39) | 446.31(344.78 to 552.44) |  | -0.79(-1.16 to -0.42) |
| Palestine | 0.95(0.72 to 1.26) | 3.34(2.53 to 4.31) |  | 250.94 |  | 238.62(180.61 to 312.48) | 249.13(190.96 to 317.7) |  | 0.46(0.32 to 0.6) |
| Panama | 3.62(2.76 to 4.65) | 10.92(8.32 to 13.97) |  | 201.37 |  | 482.71(366.92 to 619.44) | 507.87(386.21 to 647.73) |  | 0.44(0.31 to 0.58) |
| Papua New Guinea | 3.91(2.97 to 5.06) | 11.42(8.74 to 14.51) |  | 192.03 |  | 421.7(319.77 to 527.47) | 434.93(331.41 to 543.17) |  | 0.23(0.2 to 0.27) |
| Paraguay | 2.73(2.03 to 3.55) | 7.71(5.74 to 10.11) |  | 182.09 |  | 258(190.08 to 335.32) | 268.2(199.61 to 350.63) |  | 0.25(0.22 to 0.28) |
| Peru | 18.56(13.95 to 24.35) | 54.07(40.92 to 68.94) |  | 191.33 |  | 318.75(239.65 to 418.61) | 336.49(254.74 to 429.79) |  | 0.07(0.06 to 0.09) |
| Philippines | 69.58(53.48 to 86.99) | 199.95(153.51 to 251.83) |  | 187.38 |  | 483.53(375.3 to 602.1) | 495.52(387.51 to 616.71) |  | 0.04(0.03 to 0.05) |
| Poland | 97.14(74.13 to 123.06) | 98.69(80.1 to 118.34) |  | 1.6 |  | 506.9(390.28 to 632.58) | 311.55(259.16 to 368.77) |  | 0.13(0.08 to 0.19) |
| Portugal | 11.73(8.69 to 15.73) | 17.97(13.46 to 23.53) |  | 53.2 |  | 181.54(135.36 to 241.03) | 186.99(139.91 to 244.93) |  | -0.02(-0.05 to 0.01) |
| Puerto Rico | 5.9(4.39 to 7.61) | 10.88(8.31 to 13.99) |  | 84.5 |  | 347.18(259.67 to 447.6) | 366.67(280.32 to 466.25) |  | 0.12(0.11 to 0.14) |
| Qatar | 0.21(0.16 to 0.28) | 2.24(1.68 to 3.03) |  | 968.87 |  | 247.1(185.99 to 319.97) | 268.8(205.41 to 346.11) |  | 0.19(0.17 to 0.2) |
| Republic of Korea | 16.48(11.83 to 22.16) | 60.54(43.74 to 84.04) |  | 267.26 |  | 122.51(88.87 to 162.54) | 126.9(92.51 to 175.57) |  | 0.16(0.15 to 0.17) |
| Republic of Moldova | 12.26(9.57 to 15.44) | 16.2(12.57 to 19.94) |  | 32.22 |  | 629.99(496.71 to 773.44) | 630.22(494.78 to 767.84) |  | 0.43(0.3 to 0.56) |
| Romania | 55.94(44.89 to 66.93) | 71.97(57.16 to 86.84) |  | 28.66 |  | 422.31(340.4 to 503.01) | 444.22(359.05 to 529.6) |  | 0.04(0.02 to 0.05) |
| Russian Federation | 462.15(358.71 to 581.99) | 661.68(515.89 to 812.02) |  | 43.18 |  | 665.37(529.43 to 800.15) | 663.09(527.16 to 795.37) |  | 0.05(-0.01 to 0.1) |
| Rwanda | 3.42(2.57 to 4.57) | 7.42(5.57 to 9.84) |  | 116.74 |  | 260.67(197.56 to 339.38) | 260.86(197.22 to 340.57) |  | -0.02(-0.05 to 0) |
| Saint Kitts and Nevis | 0.06(0.04 to 0.08) | 0.14(0.1 to 0.18) |  | 127.99 |  | 347.05(259.25 to 449.38) | 364.77(274.7 to 471.41) |  | -0.01(-0.02 to 0) |
| Saint Lucia | 0.14(0.11 to 0.18) | 0.45(0.34 to 0.57) |  | 216.72 |  | 352.67(266.56 to 449.22) | 373.44(283.14 to 476.93) |  | 0.19(0.17 to 0.21) |
| Saint Vincent and the Grenadines | 0.11(0.09 to 0.15) | 0.29(0.22 to 0.38) |  | 156.01 |  | 348.04(264.75 to 452.69) | 369.53(280.18 to 470.82) |  | 0.23(0.21 to 0.25) |
| Samoa | 0.18(0.14 to 0.24) | 0.31(0.24 to 0.4) |  | 71.96 |  | 426.4(327.15 to 539.69) | 439.91(337.05 to 551.38) |  | 0.23(0.21 to 0.25) |
| San Marino | 0.03(0.02 to 0.04) | 0.06(0.04 to 0.07) |  | 95.66 |  | 178.38(133.06 to 235.14) | 183.11(136.51 to 238.23) |  | 0.1(0.08 to 0.13) |
| Sao Tome and Principe | 0.08(0.06 to 0.1) | 0.14(0.1 to 0.18) |  | 78.55 |  | 241.08(181.91 to 309.44) | 245.55(187.37 to 316.17) |  | 0.04(0.03 to 0.05) |
| Saudi Arabia | 8.28(6.32 to 10.69) | 32.03(24.51 to 41.06) |  | 287.07 |  | 240.65(182.79 to 310.04) | 253.45(192.65 to 326.88) |  | 0.15(0.15 to 0.16) |
| Senegal | 4.09(3.09 to 5.31) | 9.59(7.24 to 12.34) |  | 134.1 |  | 242.4(183.85 to 310.27) | 248.24(190.06 to 317.29) |  | 0.07(0.06 to 0.08) |
| Serbia | 22.69(18.37 to 27.56) | 31.82(25.69 to 38.27) |  | 40.21 |  | 391.71(321.87 to 465.59) | 415.84(340.56 to 491.33) |  | 0.1(0.05 to 0.15) |
| Seychelles | 0.12(0.09 to 0.15) | 0.3(0.23 to 0.38) |  | 156.09 |  | 457.39(348.15 to 576.44) | 481.6(366.18 to 605.95) |  | 0.14(0.12 to 0.17) |
| Sierra Leone | 2.53(1.92 to 3.24) | 4.74(3.6 to 6.07) |  | 86.82 |  | 236.52(179.91 to 303.05) | 241.26(183.57 to 312.8) |  | 0.06(0.05 to 0.07) |
| Singapore | 1.33(0.96 to 1.8) | 5.95(4.25 to 8.2) |  | 346.71 |  | 124.82(90.62 to 168.45) | 127.13(92.39 to 173.37) |  | 0.09(0.02 to 0.15) |
| Slovakia | 10.39(8.79 to 12.21) | 17.96(14.95 to 21.27) |  | 72.97 |  | 390.95(333.21 to 457.7) | 408.81(344.48 to 475.83) |  | 0.04(0 to 0.09) |
| Slovenia | 2.79(2.34 to 3.29) | 5.81(4.82 to 6.99) |  | 108.38 |  | 279.07(235.8 to 329.43) | 292.77(245.68 to 346.96) |  | 0.04(-0.01 to 0.1) |
| Solomon Islands | 0.32(0.24 to 0.42) | 0.73(0.55 to 0.92) |  | 127.03 |  | 419.53(320.44 to 530.74) | 438.43(336.1 to 555.09) |  | 0.14(0.09 to 0.19) |
| Somalia | 2.93(2.19 to 3.83) | 6.98(5.25 to 9.1) |  | 138.53 |  | 260.73(197.45 to 339.79) | 262.31(197.1 to 339.89) |  | 0.15(0.13 to 0.17) |
| South Africa | 30.24(23.11 to 38.39) | 72.55(55.33 to 94.67) |  | 139.9 |  | 335.84(256.54 to 423.13) | 345.51(267.05 to 438.3) |  | 0.01(0 to 0.02) |
| South Sudan | 4.16(3.1 to 5.57) | 5.54(4.15 to 7.13) |  | 33.12 |  | 261.17(196.11 to 339.68) | 261.86(198.84 to 335.12) |  | 0.09(0.07 to 0.11) |
| Spain | 35.76(26.14 to 47.44) | 70.44(51.44 to 91.86) |  | 96.98 |  | 143.63(106.87 to 189.9) | 177.86(129.04 to 232.5) |  | -0.01(-0.03 to 0.01) |
| Sri Lanka | 25.53(19.39 to 32.31) | 62.71(48.22 to 78.27) |  | 145.66 |  | 458.99(352.41 to 582.51) | 477.44(366.48 to 588.96) |  | 0.63(0.49 to 0.77) |
| Sudan | 11.83(8.94 to 15.59) | 26.67(20.22 to 34.73) |  | 125.43 |  | 236.41(179.71 to 309.67) | 248.03(188.23 to 323.86) |  | 0.12(0.1 to 0.14) |
| Suriname | 0.44(0.33 to 0.57) | 1.14(0.87 to 1.48) |  | 159.71 |  | 344.69(262.13 to 440.29) | 368.81(279.13 to 473.54) |  | 0.11(0.09 to 0.13) |
| Sweden | 16.05(11.86 to 21.09) | 20.15(14.85 to 26.35) |  | 25.53 |  | 256.18(191.38 to 334.8) | 225.75(166.91 to 292.73) |  | 0.29(0.26 to 0.32) |
| Switzerland | 20.51(17.82 to 23.36) | 36.87(32.03 to 42.18) |  | 79.75 |  | 479.4(418.15 to 542.46) | 481.11(420.16 to 548.56) |  | 0.01(-0.14 to 0.17) |
| Syrian Arab Republic | 6.65(5.03 to 8.84) | 18.33(13.82 to 24.12) |  | 175.8 |  | 236.69(180.73 to 310.16) | 247.71(188.29 to 322.11) |  | 0.26(0.16 to 0.36) |
| Taiwan (Province of China) | 42.19(31.51 to 55.09) | 96.58(76.95 to 119.88) |  | 128.92 |  | 466.97(363.55 to 584.12) | 468.94(377.89 to 570.44) |  | 0.09(0.08 to 0.11) |
| Tajikistan | 3.87(2.88 to 5.01) | 9.84(7.45 to 12.88) |  | 154.43 |  | 322.05(240.42 to 410.75) | 330.04(251.85 to 422.9) |  | 0(-0.02 to 0.02) |
| Thailand | 77.4(59.61 to 97.58) | 243.43(185.45 to 309.3) |  | 214.52 |  | 453.77(347.96 to 566.65) | 462.56(355.09 to 585.11) |  | 0.06(0.03 to 0.09) |
| Timor-Leste | 0.65(0.49 to 0.82) | 2.07(1.58 to 2.64) |  | 221.32 |  | 452.27(348.92 to 568.59) | 465.95(358.61 to 588.79) |  | 0.01(-0.02 to 0.03) |
| Togo | 1.43(1.09 to 1.87) | 4.34(3.2 to 5.8) |  | 202.83 |  | 238.37(181.47 to 310.78) | 242.58(182.49 to 312.92) |  | 0.09(0.02 to 0.16) |
| Tokelau | 0(0 to 0) | 0(0 to 0) |  | 6.12 |  | 424.27(321.39 to 533.01) | 438.33(338.5 to 552.59) |  | 0.05(0.03 to 0.07) |
| Tonga | 0.12(0.09 to 0.15) | 0.16(0.12 to 0.21) |  | 39.38 |  | 424.32(327.06 to 531.67) | 438.28(332.94 to 554.29) |  | 0.02(0.01 to 0.03) |
| Trinidad and Tobago | 1.45(1.11 to 1.89) | 3.73(2.81 to 4.81) |  | 156.37 |  | 355.18(270.8 to 459.94) | 371.46(279.58 to 473) |  | 0.13(0.11 to 0.15) |
| Tunisia | 6.53(4.88 to 8.71) | 17.15(12.78 to 22.61) |  | 162.56 |  | 237.96(180.27 to 312.23) | 249.58(186.77 to 323.87) |  | 0.1(0.09 to 0.12) |
| Turkey | 40.49(30.78 to 53.18) | 113.57(86.41 to 146.87) |  | 180.49 |  | 236.41(179.71 to 309.67) | 242.55(184.88 to 311.71) |  | 0.19(0.16 to 0.21) |
| Turkmenistan | 2.59(1.92 to 3.37) | 6.09(4.62 to 7.81) |  | 135.67 |  | 322.05(240.42 to 410.75) | 326.75(250.87 to 414.78) |  | 0.13(0.11 to 0.14) |
| Tuvalu | 0.01(0.01 to 0.02) | 0.02(0.02 to 0.03) |  | 67.26 |  | 422.38(319.72 to 538.44) | 441.6(337.11 to 558.23) |  | 0.11(0.09 to 0.13) |
| Uganda | 8.52(6.42 to 11.27) | 17.75(13.44 to 23.26) |  | 108.34 |  | 262.24(198.08 to 346.23) | 264.4(199.72 to 346.78) |  | 0.04(0.01 to 0.07) |
| Ukraine | 188.04(146.44 to 234.5) | 209.17(164.18 to 255.27) |  | 11.24 |  | 665.99(527.4 to 804.69) | 666.49(524.93 to 800.98) |  | 0.15(0.13 to 0.17) |
| United Arab Emirates | 0.72(0.54 to 0.93) | 11.55(8.5 to 15.69) |  | 1505.3 |  | 244.38(184.72 to 318.67) | 258.18(194.58 to 336.54) |  | -0.01(-0.03 to 0) |
| United Kingdom | 99.43(80.08 to 122.28) | 141.23(115.24 to 170.14) |  | 42.04 |  | 257.53(210.72 to 314.31) | 254.24(210.03 to 304.46) |  | -0.03(-0.05 to 0) |
| United Republic of Tanzania | 14.48(10.84 to 18.94) | 33.27(24.99 to 43.24) |  | 129.71 |  | 259.58(195.4 to 334.9) | 261.83(196.69 to 339.84) |  | 0.14(0.12 to 0.16) |
| United States of America | 268.65(220.72 to 323.88) | 577.9(485.82 to 671.88) |  | 115.11 |  | 200.44(165.59 to 239.48) | 216.78(185.21 to 251.43) |  | 0.53(0.39 to 0.67) |
| United States Virgin Islands | 0.14(0.11 to 0.18) | 0.32(0.24 to 0.41) |  | 132.07 |  | 341.26(259.79 to 441.33) | 363.61(271.47 to 465.58) |  | 0.01(0 to 0.02) |
| Uruguay | 2.53(1.89 to 3.29) | 3.43(2.57 to 4.46) |  | 35.66 |  | 142.62(107.35 to 184.94) | 151.27(114.07 to 195.96) |  | 0.49(0.4 to 0.58) |
| Uzbekistan | 15.11(11.36 to 19.62) | 41.99(31.27 to 55.04) |  | 177.84 |  | 320.08(242.48 to 415.23) | 330.15(248.01 to 424.63) |  | 0.26(0.23 to 0.29) |
| Vanuatu | 0.14(0.11 to 0.18) | 0.37(0.28 to 0.48) |  | 161.99 |  | 421.9(321.3 to 530.92) | 436.64(333.31 to 553.16) |  | 0.32(0.21 to 0.44) |
| Venezuela (Bolivarian Republic of) | 22.11(17 to 28.06) | 75.37(57.85 to 96.74) |  | 240.82 |  | 483.82(370.91 to 605.04) | 505.26(388.3 to 642.94) |  | 0.06(0.03 to 0.09) |
| Viet Nam | 85.64(65.6 to 108.13) | 251.14(198.15 to 307.95) |  | 193.24 |  | 481.06(373.05 to 599.36) | 526.76(418.32 to 642.74) |  | 0.13(0.11 to 0.16) |
| Yemen | 5.87(4.41 to 7.75) | 17.02(12.93 to 21.93) |  | 190.2 |  | 234.47(178.31 to 305.66) | 241.8(181.37 to 308.39) |  | 0.07(0.06 to 0.08) |
| Zambia | 3.98(2.99 to 5.18) | 8.98(6.76 to 11.5) |  | 125.67 |  | 260.34(196.88 to 335.4) | 263.55(196.97 to 344.47) |  | 0.01(0 to 0.02) |
| Zimbabwe | 6.7(5 to 8.85) | 10(7.62 to 12.93) |  | 49.07 |  | 312.84(238.92 to 400.71) | 323.94(246.07 to 413.17) |  | 0.11(0.09 to 0.12) |

EAPC: estimated annual percentage change; UI: uncertainty interval; CI: confidence interval.
