# Supplementary material for: Global, regional, and national burden of benign prostatic hyperplasia from 1990 to 2021 and projection to 2035
Source: BMC Urol. 2025 Feb 19;25:34. doi: 10.1186/s12894-025-01715-9 (PMC11837592; doi:10.1186/s12894-025-01715-9)
Supplement: Supplementary file 2 — Supplementary Material 2 [file 12894_2025_1715_MOESM2_ESM.docx]

**Table S2** Prevalence and age-standardized prevalence rate of benign prostatic hyperplasia in 204 countries and territories between 1990 and 2021

| Location | All ages, No. ×10^3^ (95% UI) | |  | Change (%) |  | Age-standardized rate per 100,000, No. (95% UI) | |  | EAPC (95% CI) |
| --- | --- | --- | --- | --- | --- | --- | --- | --- | --- |
|  | **1990** | **2021** |  | **1990–2021** |  | **1990** | **2021** |  | **1990–2021** |
| Afghanistan | 74.67(54.65 to 100.02) | 76.95(57.46 to 100.13) |  | 3.06 |  | 2055.58(1511.58 to 2690.75) | 2165.99(1609.42 to 2823.39) |  | 0.15(0.14 to 0.16) |
| Albania | 27.35(20.34 to 36.4) | 68.81(50.64 to 90.66) |  | 151.59 |  | 2977.68(2217.01 to 3854.88) | 3110.24(2304.32 to 4061.69) |  | 0.1(0.08 to 0.13) |
| Algeria | 121.39(89.43 to 164.03) | 380.9(277.75 to 507.22) |  | 213.79 |  | 2041.82(1514.77 to 2719.33) | 2151.71(1583.89 to 2861.07) |  | 0.14(0.13 to 0.15) |
| American Samoa | 0.35(0.26 to 0.45) | 0.88(0.66 to 1.16) |  | 152.91 |  | 3674.92(2773.13 to 4777.55) | 3946.34(3027.65 to 5144.34) |  | 0.24(0.22 to 0.26) |
| Andorra | 0.46(0.33 to 0.61) | 1.27(0.92 to 1.66) |  | 176.81 |  | 1513.93(1090.53 to 1987.41) | 1609.04(1163.11 to 2122.61) |  | 0.29(0.25 to 0.32) |
| Angola | 35.95(26.58 to 49.76) | 100.86(74.46 to 137.6) |  | 180.54 |  | 2098.85(1559.2 to 2814.94) | 2145.6(1612.66 to 2831.81) |  | 0.04(0.03 to 0.05) |
| Antigua and Barbuda | 0.69(0.51 to 0.91) | 1.57(1.14 to 2.13) |  | 127.30 |  | 2913.75(2159.62 to 3809.03) | 3091.15(2268.75 to 4145.19) |  | 0.22(0.2 to 0.24) |
| Argentina | 176.37(128.95 to 236.49) | 324.03(237.42 to 433.54) |  | 83.72 |  | 1227.34(904 to 1631.21) | 1303.5(963.09 to 1733.52) |  | 0.25(0.2 to 0.3) |
| Armenia | 29.47(21.37 to 39.53) | 52.65(38.81 to 69.5) |  | 78.68 |  | 2751.97(2029.92 to 3576.22) | 2811.28(2084.34 to 3688.01) |  | 0.07(0.04 to 0.09) |
| Australia | 168.45(120.44 to 231.07) | 429.89(307.37 to 579.31) |  | 155.20 |  | 1867.32(1352.57 to 2539.92) | 1943.65(1398.16 to 2610.17) |  | 0.12(0.1 to 0.14) |
| Austria | 160.56(115.72 to 208.42) | 373.48(337.97 to 409.63) |  | 132.61 |  | 3500.25(2518.5 to 4536.48) | 4602.85(4151.19 to 5051.69) |  | 0.57(0.45 to 0.69) |
| Azerbaijan | 50.02(36.79 to 65.82) | 121.21(89.72 to 163.59) |  | 142.32 |  | 2749.35(2035.58 to 3612.67) | 2815.11(2104.39 to 3686.56) |  | 0.07(0.05 to 0.09) |
| Bahamas | 1.8(1.3 to 2.41) | 5.41(3.97 to 7.34) |  | 199.97 |  | 2921.72(2126.61 to 3947.06) | 3073.85(2292.73 to 4132.86) |  | 0.23(0.21 to 0.24) |
| Bahrain | 1.76(1.3 to 2.38) | 11.85(8.69 to 16.16) |  | 573.73 |  | 2121.26(1575.86 to 2783.67) | 2304.66(1691.41 to 3046.66) |  | 0.12(0.12 to 0.13) |
| Bangladesh | 679.77(501.62 to 909.74) | 2049.42(1499.99 to 2705.81) |  | 201.49 |  | 2779.19(2051.76 to 3707.91) | 2878.65(2127.36 to 3762.64) |  | 0.18(0.16 to 0.2) |
| Barbados | 3.73(2.76 to 4.91) | 7.53(5.53 to 9.84) |  | 101.96 |  | 2921.2(2157.59 to 3814.47) | 3065.54(2262.72 to 3984.38) |  | 0(-0.02 to 0.01) |
| Belarus | 265.4(202.03 to 342.09) | 356.36(271.12 to 460.15) |  | 34.28 |  | 5872.03(4478.93 to 7451.85) | 5836.73(4523.09 to 7414.75) |  | 0.31(0.26 to 0.35) |
| Belgium | 254.37(154.26 to 322.55) | 436.96(282.73 to 544.07) |  | 71.78 |  | 3826.34(2334.99 to 4843.62) | 4095.96(2675.57 to 5106.04) |  | 0.22(0.2 to 0.24) |
| Belize | 1.28(0.94 to 1.69) | 4.28(3.18 to 5.81) |  | 234.31 |  | 2852.16(2099.56 to 3780.99) | 3024.73(2248.04 to 4062.66) |  | 0.05(0.04 to 0.07) |
| Benin | 19.35(14.53 to 26.39) | 47.23(35.09 to 64.44) |  | 144.08 |  | 2091.76(1579.47 to 2852.88) | 2137.62(1595.02 to 2847.42) |  | 0.17(0.15 to 0.19) |
| Bermuda | 0.75(0.55 to 1) | 1.88(1.39 to 2.53) |  | 149.55 |  | 2852.95(2079.94 to 3761.84) | 2987.22(2216.81 to 4023.11) |  | 0.15(0.14 to 0.16) |
| Bhutan | 3.02(2.24 to 4.04) | 8.63(6.5 to 11.24) |  | 186.04 |  | 2771.19(2083.76 to 3629.27) | 2905.09(2173.46 to 3788.12) |  | 0.17(0.16 to 0.19) |
| Bolivia (Plurinational State of) | 38.16(27.78 to 50.31) | 120.91(86.87 to 162.79) |  | 216.87 |  | 2737.41(2023.87 to 3616.76) | 2895.43(2096.61 to 3848.11) |  | 0.21(0.18 to 0.24) |
| Bosnia and Herzegovina | 49.16(36.8 to 64.42) | 92.01(68.28 to 120.25) |  | 87.16 |  | 3023.46(2254.94 to 3939.88) | 3237.47(2417.64 to 4172.15) |  | 0.1(0.09 to 0.12) |
| Botswana | 8.08(6.01 to 10.45) | 20.75(15.39 to 26.67) |  | 156.80 |  | 3360.31(2550.15 to 4255.92) | 3458.46(2604.53 to 4392.06) |  | -0.33(-0.44 to -0.21) |
| Brazil | 912.98(764.15 to 1111.66) | 2111.13(1763.87 to 2572.96) |  | 131.24 |  | 2328.08(1935.18 to 2822.22) | 1884.24(1566.57 to 2287.75) |  | 0.12(0.11 to 0.13) |
| Brunei Darussalam | 0.5(0.35 to 0.67) | 1.78(1.26 to 2.43) |  | 259.45 |  | 1075.79(754.33 to 1442.53) | 1118.07(793.57 to 1489.02) |  | 0.12(0.09 to 0.15) |
| Bulgaria | 175.9(131.9 to 232.28) | 200(147.86 to 260.84) |  | 13.70 |  | 3008.53(2254.15 to 3893.75) | 3171.24(2387.87 to 4115.56) |  | 0.04(0.04 to 0.05) |
| Burkina Faso | 41.92(31.12 to 56.87) | 87.31(64.93 to 115.3) |  | 108.29 |  | 2093.65(1580.95 to 2803.74) | 2133.88(1594.34 to 2811.67) |  | -0.03(-0.04 to -0.01) |
| Burundi | 22.6(16.66 to 30.35) | 52.43(38.18 to 71.34) |  | 131.95 |  | 2272.72(1670.85 to 3023.14) | 2265.24(1654.42 to 3008.6) |  | 0.1(0.09 to 0.12) |
| Cabo Verde | 2.11(1.54 to 2.79) | 3.86(2.89 to 5.13) |  | 83.13 |  | 2086.4(1536.99 to 2770.15) | 2158.96(1597.06 to 2827) |  | 0.06(0.05 to 0.07) |
| Cambodia | 68.52(50.52 to 90.35) | 196.74(146.19 to 256.74) |  | 187.10 |  | 3954.14(2962.66 to 5128.89) | 4103.64(3045.75 to 5280.62) |  | 0.24(0.19 to 0.28) |
| Cameroon | 42.29(31.45 to 55.95) | 118.3(87.32 to 160.38) |  | 179.71 |  | 2082.87(1531.81 to 2756.08) | 2133(1590.54 to 2820) |  | 0.04(0.03 to 0.05) |
| Canada | 249.24(183.34 to 330.22) | 621.44(467.63 to 810.64) |  | 149.34 |  | 1742.69(1292.04 to 2283.04) | 1828.29(1373.41 to 2342.13) |  | 0.04(0.03 to 0.05) |
| Central African Republic | 9.95(7.21 to 13.68) | 18.37(13.45 to 25.45) |  | 84.63 |  | 2106.36(1569.43 to 2781.37) | 2156.65(1604.1 to 2873.1) |  | 0.28(0.24 to 0.32) |
| Chad | 28.21(20.45 to 37.75) | 62.35(45.91 to 84.83) |  | 121.05 |  | 2092.71(1524.17 to 2776.5) | 2134.79(1566.6 to 2861.75) |  | -0.24(-0.41 to -0.07) |
| Chile | 67.61(49.33 to 92.28) | 193.02(141.39 to 262.45) |  | 185.47 |  | 1539.5(1133.25 to 2076.04) | 1640.93(1206.6 to 2214.4) |  | 0.14(0.13 to 0.15) |
| China | 9940.46(7241.44 to 13182.44) | 23111.2(18003.85 to 30074.67) |  | 132.50 |  | 2880.29(2141.07 to 3730.58) | 2315.23(1822.16 to 2946.62) |  | 0.18(0.16 to 0.2) |
| Colombia | 338.84(252.54 to 441.64) | 1110.78(830.37 to 1436.27) |  | 227.82 |  | 4332.05(3257.9 to 5609.85) | 4515.1(3394.98 to 5779.55) |  | -0.01(-0.02 to 0) |
| Comoros | 2.05(1.51 to 2.78) | 4.77(3.55 to 6.33) |  | 132.78 |  | 2265.41(1676.1 to 3014.13) | 2267.65(1688.32 to 2989.19) |  | 0.03(0.01 to 0.04) |
| Congo | 9.2(6.74 to 12.59) | 25.25(18.78 to 33.93) |  | 174.61 |  | 2116.6(1564.11 to 2833.98) | 2158.85(1594.54 to 2876.08) |  | 0.18(0.17 to 0.19) |
| Cook Islands | 0.21(0.16 to 0.28) | 0.48(0.36 to 0.63) |  | 126.14 |  | 3511.62(2662.55 to 4507.43) | 3690.26(2775.65 to 4784.46) |  | 0.18(0.17 to 0.19) |
| Costa Rica | 34.71(25.46 to 45.23) | 114.14(83.69 to 148.02) |  | 228.84 |  | 4313.58(3177.98 to 5595.53) | 4545.5(3354.53 to 5881.47) |  | 0.01(-0.07 to 0.09) |
| Croatia | 77.09(66.55 to 88.13) | 122.15(105.46 to 141.61) |  | 58.44 |  | 3266.79(2855.17 to 3734.83) | 3056.94(2640.28 to 3525.82) |  | 0.17(0.15 to 0.19) |
| Cuba | 148.38(109.5 to 195.84) | 281.24(207.09 to 370.28) |  | 89.54 |  | 2919.48(2158.27 to 3845.83) | 3052.73(2243.74 to 4031.38) |  | 0.11(0.1 to 0.12) |
| Cyprus | 6.14(5.05 to 7.47) | 16.19(13.03 to 19.31) |  | 163.81 |  | 1500.9(1237.43 to 1817.56) | 1554.45(1252.49 to 1845.57) |  | 0.23(0.21 to 0.25) |
| Czechia | 218.31(167.67 to 272.3) | 409.23(312.61 to 500.69) |  | 87.45 |  | 3842.43(2995.91 to 4721.15) | 4170.98(3227.98 to 5085.5) |  | 0.1(0.09 to 0.1) |
| Cote d'Ivoire | 39.89(30 to 54.31) | 112.53(82.45 to 151.07) |  | 182.07 |  | 2091.76(1579.47 to 2852.88) | 2135.26(1566.63 to 2813.38) |  | 0.03(0.01 to 0.04) |
| Democratic People's Republic of Korea | 150.4(111.62 to 195.91) | 384.95(288.21 to 503.78) |  | 155.95 |  | 3214.56(2417.86 to 4131.37) | 3298.13(2516.51 to 4261.13) |  | 0.32(0.27 to 0.37) |
| Democratic Republic of the Congo | 141.65(103.52 to 193.16) | 316.77(232.95 to 433.62) |  | 123.63 |  | 2108.35(1545.43 to 2832.47) | 2139.37(1597.35 to 2842.44) |  | 0.01(-0.01 to 0.02) |
| Denmark | 57.64(39.87 to 81.46) | 97.66(67.01 to 136.62) |  | 69.42 |  | 1613.49(1126.98 to 2273.57) | 1710.66(1180.44 to 2380.9) |  | 0.26(0.24 to 0.28) |
| Djibouti | 1.34(0.98 to 1.82) | 7.13(5.28 to 9.53) |  | 431.30 |  | 2255.8(1671.88 to 2980.12) | 2286.27(1681.61 to 2981.71) |  | 0.2(0.17 to 0.22) |
| Dominica | 0.7(0.51 to 0.93) | 1.28(0.92 to 1.75) |  | 82.24 |  | 2926.45(2160.84 to 3849.86) | 3140.94(2298.2 to 4150.62) |  | 0.01(-0.09 to 0.12) |
| Dominican Republic | 49.02(35.54 to 65.66) | 137.47(101.59 to 178.64) |  | 180.46 |  | 2825.01(2059.56 to 3750.79) | 2966.68(2195.01 to 3860.89) |  | 0.13(0.11 to 0.14) |
| Ecuador | 73.39(54.66 to 98.68) | 223.59(162.86 to 295.46) |  | 204.66 |  | 3010.31(2240.66 to 4021.28) | 2906.53(2127.96 to 3835.89) |  | 0.23(0.22 to 0.24) |
| Egypt | 254.2(189.91 to 348.65) | 675.52(488.36 to 909.11) |  | 165.74 |  | 2016.8(1508.71 to 2696.66) | 2114.72(1534.02 to 2801.14) |  | 0.05(0.04 to 0.06) |
| El Salvador | 55.9(41.81 to 74.06) | 116.85(88.47 to 151.66) |  | 109.04 |  | 4268.82(3197.69 to 5639.05) | 4550.09(3429.47 to 5907.12) |  | 0(-0.02 to 0.01) |
| Equatorial Guinea | 1.67(1.23 to 2.29) | 4.1(3.05 to 5.54) |  | 144.97 |  | 2101.67(1572.91 to 2813.52) | 2156.65(1604.1 to 2873.1) |  | 0.03(0.01 to 0.05) |
| Eritrea | 8.73(6.44 to 11.85) | 22(16.09 to 29.74) |  | 152.10 |  | 2255.14(1686.12 to 2996.71) | 2280.97(1653.2 to 3042.98) |  | -0.04(-0.06 to -0.01) |
| Estonia | 42(31.47 to 54.93) | 60.76(46.65 to 77.47) |  | 44.67 |  | 5881.81(4436.78 to 7555.92) | 5905.07(4545.29 to 7500.26) |  | 0.21(0.19 to 0.24) |
| Eswatini | 3.24(2.4 to 4.49) | 5.93(4.31 to 8.08) |  | 83.08 |  | 2845.47(2149.82 to 3872.36) | 2941.42(2151.53 to 3941.84) |  | 0.22(0.21 to 0.22) |
| Ethiopia | 239.01(172.71 to 325.48) | 500.21(368.47 to 670.31) |  | 109.29 |  | 2472.11(1839.18 to 3288.96) | 2462.88(1813 to 3268.14) |  | 0.21(0.19 to 0.22) |
| Fiji | 5.17(3.86 to 6.77) | 12.08(8.87 to 15.92) |  | 133.70 |  | 3576.49(2696.72 to 4597.01) | 3806.92(2893.65 to 4871.79) |  | 0.25(0.22 to 0.29) |
| Finland | 107.48(95.77 to 120.06) | 244.8(218.57 to 270.33) |  | 127.76 |  | 3854.24(3439.21 to 4283.95) | 4053.31(3628.82 to 4476.1) |  | 0.05(0.04 to 0.07) |
| France | 528.08(384.75 to 716.77) | 970.89(698.38 to 1291.68) |  | 83.86 |  | 1504.68(1104.78 to 2022.48) | 1584.68(1154.84 to 2105.29) |  | 0.12(0.05 to 0.19) |
| Gabon | 5.28(3.9 to 7.14) | 10.22(7.53 to 14.21) |  | 93.74 |  | 2110(1576.6 to 2794.74) | 2165.91(1619.19 to 2858.78) |  | 0.34(0.3 to 0.37) |
| Gambia | 3.57(2.62 to 4.8) | 9.22(6.76 to 12.45) |  | 158.46 |  | 2084.55(1545.89 to 2788.56) | 2124.88(1566.61 to 2872.51) |  | 0.06(0.06 to 0.07) |
| Georgia | 69.8(51.26 to 91.7) | 79.03(67.89 to 92.22) |  | 13.22 |  | 2973.73(2222.44 to 3840.96) | 3236.14(2790.53 to 3759.43) |  | 0.19(0.1 to 0.29) |
| Germany | 751.29(544.03 to 1016.7) | 1438.55(1053.39 to 1925.88) |  | 91.48 |  | 1537.59(1112.62 to 2072.85) | 1653.7(1204.86 to 2212.1) |  | 0.28(0.24 to 0.33) |
| Ghana | 58.12(42.92 to 78.13) | 144.52(107.04 to 192.8) |  | 148.64 |  | 2101(1543.3 to 2784.71) | 2143.78(1593.75 to 2793.9) |  | 0.23(0.22 to 0.25) |
| Greece | 109.67(79.38 to 147.63) | 184.12(144.1 to 230.69) |  | 67.89 |  | 1519.83(1107.18 to 2031.03) | 1751.27(1382.08 to 2192.57) |  | 0.1(0.08 to 0.11) |
| Greenland | 0.26(0.18 to 0.34) | 0.7(0.51 to 0.95) |  | 170.34 |  | 1730.27(1298.97 to 2273.3) | 1837.05(1376.38 to 2390.61) |  | 0.25(0.24 to 0.26) |
| Grenada | 0.89(0.66 to 1.18) | 1.68(1.22 to 2.28) |  | 87.90 |  | 2923.07(2161.95 to 3841.52) | 3128.94(2301.88 to 4154.88) |  | 0.04(0.03 to 0.06) |
| Guam | 1.11(0.81 to 1.47) | 3.47(2.61 to 4.5) |  | 212.11 |  | 3435.83(2575.52 to 4392.53) | 3525.52(2667.18 to 4505.3) |  | 0.05(0.04 to 0.06) |
| Guatemala | 67.74(49.71 to 89.1) | 229.02(170.83 to 295.37) |  | 238.07 |  | 4290.01(3217.17 to 5530.68) | 4588.69(3439.66 to 5912.91) |  | 0.25(0.23 to 0.27) |
| Guinea | 34.07(24.88 to 46.18) | 58.04(42.85 to 77.6) |  | 70.35 |  | 2082.16(1535.79 to 2807.05) | 2135.46(1595.11 to 2820.48) |  | 0.22(0.21 to 0.23) |
| Guinea-Bissau | 3.79(2.79 to 5.1) | 6.24(4.63 to 8.43) |  | 64.61 |  | 2086.4(1536.99 to 2770.15) | 2132.73(1593.59 to 2845.51) |  | 0.19(0.18 to 0.2) |
| Guyana | 4.95(3.63 to 6.66) | 9.04(6.57 to 12.26) |  | 82.69 |  | 2962.94(2187.89 to 3934.1) | 3163.25(2326.72 to 4182.21) |  | 0.14(0.11 to 0.17) |
| Haiti | 41.96(30.7 to 55.35) | 95.17(69.43 to 127.83) |  | 126.78 |  | 2918.32(2145.04 to 3797.04) | 3096.05(2265.48 to 4123.73) |  | 0.33(0.28 to 0.38) |
| Honduras | 40.24(30.08 to 52.52) | 129.37(95.94 to 169.19) |  | 221.47 |  | 4342.73(3260.52 to 5632.82) | 4592.38(3433.87 to 5945.4) |  | 0.22(0.18 to 0.26) |
| Hungary | 185.01(136.48 to 243.78) | 260.76(192.77 to 337.75) |  | 40.94 |  | 3031.09(2246.49 to 3927.57) | 3198.21(2398.27 to 4113.49) |  | -0.44(-0.7 to -0.17) |
| Iceland | 1.93(1.41 to 2.59) | 4.41(3.14 to 6.01) |  | 128.85 |  | 1452.54(1066.55 to 1942.01) | 1547.01(1108.99 to 2097.33) |  | 0.1(0.08 to 0.12) |
| India | 7810.48(5781.62 to 10403.23) | 21904.37(16228.65 to 28462.31) |  | 180.45 |  | 3562.35(2637.76 to 4639.87) | 3948.21(2974.65 to 5071.17) |  | 0.33(0.29 to 0.36) |
| Indonesia | 2384.24(1777.53 to 3089.88) | 4346.74(3370.8 to 5635.24) |  | 82.31 |  | 5586.8(4289.05 to 7049.4) | 3945.31(3077.25 to 5009.75) |  | 0.12(0.11 to 0.14) |
| Iran (Islamic Republic of) | 290.18(210.26 to 400.93) | 848.24(632.11 to 1140.28) |  | 192.31 |  | 2218.83(1653.9 to 2965.09) | 2315.17(1726.72 to 3094.39) |  | 0.29(0.26 to 0.32) |
| Iraq | 73.93(55.1 to 98.71) | 233.1(170.19 to 316.37) |  | 215.28 |  | 2074.45(1549.68 to 2759.66) | 2168.62(1596.99 to 2910.23) |  | 0.03(-0.01 to 0.07) |
| Ireland | 28.8(20.82 to 38.42) | 61.96(44.79 to 83.45) |  | 115.16 |  | 1501.45(1097.97 to 2004.9) | 1610.88(1176.71 to 2145.93) |  | 0.25(0.24 to 0.27) |
| Israel | 34.45(24.79 to 46.56) | 92.53(66.68 to 124.16) |  | 168.58 |  | 1520.5(1101.32 to 2049.51) | 1620.24(1175.61 to 2194.73) |  | -0.11(-0.19 to -0.02) |
| Italy | 1330.58(1090.78 to 1648.74) | 1988.98(1729.06 to 2311.31) |  | 49.48 |  | 3356.36(2763.35 to 4146.48) | 3300.2(2857.29 to 3851.98) |  | 0.24(0.14 to 0.34) |
| Jamaica | 24.23(17.87 to 31.93) | 45.04(32.72 to 61.17) |  | 85.92 |  | 2881.49(2130.06 to 3826.3) | 3081.94(2243.23 to 4144.12) |  | 0.07(0.05 to 0.09) |
| Japan | 1007.46(760.27 to 1340.94) | 1911.48(1497.36 to 2450.94) |  | 89.73 |  | 1344.29(1021.77 to 1780.92) | 1221.55(948.04 to 1575.24) |  | -0.01(-0.02 to 0) |
| Jordan | 12.5(9.23 to 16.52) | 88.85(66.51 to 115.45) |  | 610.61 |  | 2068.84(1540.4 to 2720.52) | 2436.37(1827.85 to 3135.82) |  | 0.1(0.08 to 0.12) |
| Kazakhstan | 117.45(86.94 to 155.94) | 195.48(142.79 to 261.14) |  | 66.44 |  | 2761.81(2036.71 to 3633.49) | 2840.03(2127.92 to 3732.29) |  | 0.18(0.17 to 0.18) |
| Kenya | 109.94(81.37 to 145.38) | 287.94(211.61 to 388.65) |  | 161.91 |  | 2935.25(2185.46 to 3842.44) | 2949.96(2184.74 to 3870.69) |  | 0.12(0.1 to 0.14) |
| Kiribati | 0.47(0.35 to 0.62) | 0.91(0.68 to 1.2) |  | 94.21 |  | 3534.54(2661.94 to 4528.35) | 3709.77(2781.96 to 4711.22) |  | -0.01(-0.03 to 0.02) |
| Kuwait | 6.56(4.85 to 8.9) | 33.21(24.89 to 44.61) |  | 406.44 |  | 2093.96(1553.54 to 2811.43) | 2201.88(1633.89 to 2939.07) |  | 0.09(0.08 to 0.11) |
| Kyrgyzstan | 25.69(19.05 to 34.26) | 45.67(33.75 to 59.97) |  | 77.77 |  | 2396.17(1805.78 to 3121.54) | 2407.6(1790.26 to 3132.58) |  | 0.04(0.02 to 0.06) |
| Lao People's Democratic Republic | 36.78(27.63 to 48.29) | 84.06(62.59 to 109.35) |  | 128.53 |  | 3993.23(3028.18 to 5157.05) | 4123.62(3078.53 to 5339.78) |  | 0.12(0.11 to 0.13) |
| Latvia | 73.2(56.92 to 93.71) | 86.68(66.01 to 110.75) |  | 18.42 |  | 5873.41(4538.22 to 7434.92) | 5894.3(4525.32 to 7497.12) |  | 0.09(0.08 to 0.1) |
| Lebanon | 20.98(15.33 to 28.41) | 58.24(43.78 to 76.32) |  | 177.66 |  | 2055.47(1510.35 to 2749.23) | 2151.03(1594.74 to 2844) |  | 0.07(0.06 to 0.08) |
| Lesotho | 8.3(6.04 to 11.32) | 11.34(8.24 to 15.58) |  | 36.68 |  | 2826.31(2087.16 to 3758.07) | 2926.05(2157.43 to 3930.64) |  | 0.18(0.17 to 0.19) |
| Liberia | 12.96(9.57 to 17.46) | 20.76(15.15 to 27.88) |  | 60.14 |  | 2106.34(1565.78 to 2814.89) | 2156.05(1589.42 to 2875.99) |  | 0.05(0.03 to 0.06) |
| Libya | 18.97(13.99 to 25.25) | 51.97(38.08 to 68.98) |  | 173.93 |  | 2061.79(1518.62 to 2718.92) | 2190.07(1604.2 to 2935.9) |  | 0.47(0.42 to 0.52) |
| Lithuania | 111.96(96.39 to 129.33) | 145.38(124.1 to 170.46) |  | 29.85 |  | 6661.29(5705.56 to 7713.35) | 6719.37(5741.66 to 7854.98) |  | -0.01(-0.02 to 0) |
| Luxembourg | 3.64(2.65 to 4.97) | 8.88(6.4 to 12.05) |  | 143.54 |  | 1591.75(1166.63 to 2147.75) | 1771.26(1278.93 to 2399.55) |  | 0.02(0.01 to 0.03) |
| Madagascar | 54.64(40.52 to 73.66) | 109.31(80.07 to 149.69) |  | 100.06 |  | 2260.58(1700.47 to 2987.9) | 2268.74(1697.4 to 3071.72) |  | 0.06(0.05 to 0.08) |
| Malawi | 37.63(27.6 to 51.27) | 67.65(49.39 to 89.86) |  | 79.79 |  | 2269.63(1685.46 to 3011.57) | 2288.25(1693.62 to 3018.02) |  | 0.05(0.04 to 0.06) |
| Malaysia | 162.97(122.28 to 210.46) | 573.58(421.12 to 752.93) |  | 251.96 |  | 4028.04(3033.11 to 5185.33) | 4155.83(3099.05 to 5397.61) |  | 0.03(0.02 to 0.04) |
| Maldives | 1.91(1.4 to 2.51) | 6.51(4.9 to 8.49) |  | 241.09 |  | 3981.63(2954.96 to 5155.24) | 4079.98(3052.1 to 5347.89) |  | 0.18(0.17 to 0.2) |
| Mali | 39.71(28.97 to 53.49) | 90.32(66.12 to 121) |  | 127.48 |  | 2089.71(1531.92 to 2761.59) | 2124.15(1567.91 to 2795.03) |  | 0.26(0.24 to 0.27) |
| Malta | 4.15(3.39 to 4.8) | 10.71(8.6 to 12.57) |  | 158.03 |  | 2147.82(1753.36 to 2471.9) | 2256.62(1816.84 to 2635.56) |  | 0.01(0 to 0.03) |
| Marshall Islands | 0.25(0.18 to 0.33) | 0.58(0.42 to 0.75) |  | 134.99 |  | 3646.95(2764.76 to 4720.74) | 3940.52(2975.77 to 5031.13) |  | 0.2(0.18 to 0.21) |
| Mauritania | 9.51(6.85 to 12.62) | 21.9(16.35 to 29.84) |  | 130.36 |  | 2078.18(1522.78 to 2742.85) | 2106.26(1584.2 to 2825.65) |  | 0.08(0.02 to 0.13) |
| Mauritius | 12.5(9.12 to 16.52) | 37.21(27.84 to 49.11) |  | 197.76 |  | 4065.11(3052.81 to 5233.35) | 4309.12(3231.7 to 5590.31) |  | 0.03(0.01 to 0.05) |
| Mexico | 959.26(780.07 to 1179.92) | 2863.67(2376.89 to 3463.96) |  | 198.53 |  | 5064.86(4095.14 to 6224.96) | 5001.63(4149.55 to 6018.85) |  | 0.14(0.12 to 0.17) |
| Micronesia (Federated States of) | 0.74(0.54 to 0.98) | 1.08(0.8 to 1.43) |  | 45.28 |  | 3479.17(2627.39 to 4487.42) | 3683.7(2770.84 to 4761) |  | 0.13(0.13 to 0.14) |
| Monaco | 0.46(0.33 to 0.6) | 0.73(0.52 to 0.97) |  | 60.01 |  | 1508.5(1094.11 to 1998.09) | 1609.04(1163.11 to 2122.61) |  | 0.02(0.01 to 0.03) |
| Mongolia | 11.84(8.75 to 15.52) | 23.65(17.75 to 30.95) |  | 99.67 |  | 2693.48(2007.59 to 3488.81) | 2725.48(2057.9 to 3547.51) |  | 0.07(0.07 to 0.08) |
| Montenegro | 7.94(5.89 to 10.31) | 14.52(10.7 to 18.95) |  | 82.77 |  | 3043.24(2249.68 to 3936.97) | 3212.47(2400.81 to 4139.15) |  | 0.05(0.03 to 0.07) |
| Morocco | 139.88(100.64 to 186.67) | 358.91(258.73 to 482.06) |  | 156.58 |  | 2031.75(1464.68 to 2696.12) | 2137.41(1560.3 to 2824.05) |  | 0.06(-0.01 to 0.13) |
| Mozambique | 59.56(42.71 to 80.25) | 101.09(74.81 to 136.03) |  | 69.72 |  | 2258.49(1657.49 to 3011.45) | 2284.67(1723.65 to 3027.58) |  | 0.24(0.21 to 0.27) |
| Myanmar | 400.13(295.06 to 525.87) | 821.19(608.41 to 1101.06) |  | 105.23 |  | 4005.36(2970.12 to 5123.33) | 4103.58(3072.93 to 5406.54) |  | -0.35(-0.47 to -0.23) |
| Namibia | 7.9(5.67 to 10.9) | 15.66(11.55 to 21.3) |  | 98.18 |  | 2831.15(2050.2 to 3798.2) | 2919.6(2150.74 to 3937.86) |  | 0.2(0.19 to 0.21) |
| Nauru | 0.07(0.05 to 0.1) | 0.08(0.06 to 0.1) |  | 1.73 |  | 3471.05(2614.19 to 4463.09) | 3634.58(2766.45 to 4627.35) |  | 0.03(0.02 to 0.05) |
| Nepal | 112.66(86.57 to 144.51) | 265.74(210.72 to 327.74) |  | 135.89 |  | 2491.91(1899 to 3154.02) | 2407.13(1922.21 to 2951.05) |  | 0(-0.01 to 0.02) |
| Netherlands | 131.01(94.32 to 178) | 272.96(195.23 to 360.03) |  | 108.35 |  | 1521.73(1102.52 to 2060.73) | 1595.3(1147.28 to 2091.22) |  | 0.13(0.12 to 0.14) |
| New Zealand | 45.51(36.76 to 56.01) | 90.65(76.84 to 107.29) |  | 99.17 |  | 2533.03(2063.55 to 3103.88) | 2171.74(1851.19 to 2565.83) |  | 0.05(0.04 to 0.06) |
| Nicaragua | 28.33(21 to 37.14) | 94.72(69.56 to 123.93) |  | 234.33 |  | 4325.41(3229.86 to 5618.88) | 4567.17(3387.77 to 5934.49) |  | 0.1(0.09 to 0.12) |
| Niger | 27.67(20.28 to 37.41) | 78.77(57.86 to 107.48) |  | 184.71 |  | 2072.04(1549 to 2751.88) | 2097.03(1547.91 to 2780.93) |  | 0.17(0.16 to 0.18) |
| Nigeria | 488.73(360.35 to 667.75) | 881.56(653.69 to 1187.5) |  | 80.38 |  | 2284.68(1692.76 to 3060.64) | 2305.47(1706.42 to 3073.13) |  | 0.13(0.12 to 0.14) |
| Niue | 0.03(0.02 to 0.04) | 0.04(0.03 to 0.05) |  | 10.67 |  | 3578.86(2693.38 to 4572) | 3828.24(2897.97 to 4913.86) |  | 0.2(0.18 to 0.21) |
| North Macedonia | 25.79(18.95 to 34.09) | 52.75(38.69 to 69.99) |  | 104.55 |  | 3035.92(2250.15 to 3961.11) | 3238.41(2398.7 to 4184.49) |  | 0.12(0.11 to 0.14) |
| Northern Mariana Islands | 0.24(0.18 to 0.31) | 0.88(0.65 to 1.17) |  | 262.73 |  | 3482.86(2612.77 to 4505.61) | 3610.44(2691.27 to 4666.98) |  | 0.12(0.11 to 0.14) |
| Norway | 157.69(123.32 to 197.36) | 255.58(201.26 to 318.52) |  | 62.08 |  | 5193.97(4069.26 to 6475) | 5283.32(4158.96 to 6604.19) |  | 0.19(0.17 to 0.21) |
| Oman | 6.48(4.78 to 8.62) | 20.6(15.28 to 27.45) |  | 217.67 |  | 2052(1531.18 to 2721.1) | 2144.06(1586.7 to 2853.04) |  | 0.04(0.01 to 0.06) |
| Pakistan | 931.6(692.93 to 1234.08) | 1887.56(1393.11 to 2521.88) |  | 102.61 |  | 3053.69(2291.07 to 4004.32) | 3190.65(2371.6 to 4138.27) |  | 0.19(0.17 to 0.21) |
| Palau | 0.16(0.11 to 0.21) | 0.39(0.29 to 0.5) |  | 151.54 |  | 3518.22(2646.76 to 4557.93) | 3719.87(2770.57 to 4711.91) |  | -0.82(-1.25 to -0.39) |
| Palestine | 7.76(5.73 to 10.37) | 24.81(18.31 to 33.7) |  | 219.74 |  | 2058.79(1537.17 to 2734.61) | 2156(1590.74 to 2860.18) |  | 0.32(0.28 to 0.36) |
| Panama | 30.95(23.15 to 40.35) | 96.22(71.72 to 125.51) |  | 210.91 |  | 4311.39(3243.82 to 5603.94) | 4564.16(3401.49 to 5934.45) |  | 0.3(0.26 to 0.34) |
| Papua New Guinea | 26.66(19.69 to 35.3) | 79.61(60.34 to 102.56) |  | 198.58 |  | 3480.05(2605.38 to 4488.87) | 3605.95(2736.43 to 4545.31) |  | 0.22(0.19 to 0.24) |
| Paraguay | 21.37(15.65 to 28.59) | 59.44(43.33 to 78.78) |  | 178.11 |  | 2146.8(1578.69 to 2855.26) | 2227.81(1623.42 to 2970.48) |  | 0.29(0.25 to 0.32) |
| Peru | 147.3(107.47 to 197.83) | 446.36(330.78 to 576.4) |  | 203.03 |  | 2707.64(1981.41 to 3632.94) | 2858.14(2121.84 to 3675.09) |  | 0.09(0.08 to 0.1) |
| Philippines | 558.79(421.24 to 730.57) | 1567.52(1189.93 to 2051.76) |  | 180.52 |  | 4334.73(3260.25 to 5611.21) | 4477.42(3402.99 to 5743.8) |  | 0.04(0.03 to 0.05) |
| Poland | 790.37(590.41 to 1035.61) | 801.97(672.32 to 964.23) |  | 1.47 |  | 4474.92(3378.41 to 5731.81) | 2591.93(2187.73 to 3090.7) |  | 0.08(0.07 to 0.09) |
| Portugal | 98.21(71.1 to 133.46) | 175.24(124.81 to 235.65) |  | 78.44 |  | 1549.96(1136.06 to 2087.62) | 1652.21(1189.36 to 2200.86) |  | 0.01(-0.01 to 0.02) |
| Puerto Rico | 50.67(37.45 to 67.28) | 102.02(75.07 to 133.52) |  | 101.33 |  | 2944.04(2181.96 to 3870.39) | 3119.11(2304.6 to 4057.52) |  | 0.16(0.14 to 0.17) |
| Qatar | 1.28(0.94 to 1.71) | 13.16(9.54 to 17.87) |  | 926.09 |  | 2144.4(1581.81 to 2841.41) | 2349.41(1719.24 to 3074.86) |  | 0.24(0.23 to 0.24) |
| Republic of Korea | 110.76(77.81 to 150.59) | 462.24(329.4 to 635.74) |  | 317.35 |  | 996.95(704.21 to 1339.1) | 1025(736.08 to 1392.65) |  | 0.2(0.19 to 0.21) |
| Republic of Moldova | 101.64(76.86 to 133.3) | 143.43(107.48 to 182.71) |  | 41.11 |  | 5927.23(4576.19 to 7622.94) | 5906.53(4494.51 to 7518.92) |  | 0.29(0.25 to 0.32) |
| Romania | 449.98(370.98 to 540.45) | 622.38(522.45 to 742.52) |  | 38.31 |  | 3666.15(3026.94 to 4359.98) | 3823.56(3219.52 to 4529.06) |  | 0.05(0.04 to 0.06) |
| Russian Federation | 3688.07(2798.12 to 4795.42) | 5806.01(4394.38 to 7367.91) |  | 57.43 |  | 6302.17(4849.8 to 7904.29) | 6279.89(4840.55 to 7837.42) |  | 0.12(0.11 to 0.13) |
| Rwanda | 26.34(19.13 to 36.13) | 55.15(40.27 to 75.15) |  | 109.41 |  | 2260.16(1671.71 to 3016.27) | 2257.85(1666.11 to 2997.23) |  | 0(-0.02 to 0.01) |
| Saint Kitts and Nevis | 0.54(0.38 to 0.73) | 0.98(0.71 to 1.36) |  | 82.35 |  | 2947.01(2149.37 to 3925.04) | 3091.15(2268.75 to 4145.19) |  | -0.02(-0.03 to 0) |
| Saint Lucia | 1.19(0.87 to 1.56) | 3.63(2.68 to 4.75) |  | 205.77 |  | 3005.75(2222.24 to 3938.95) | 3180.79(2360.07 to 4157.67) |  | 0.17(0.16 to 0.19) |
| Saint Vincent and the Grenadines | 0.95(0.69 to 1.27) | 2.36(1.72 to 3.17) |  | 147.15 |  | 2964.15(2148.09 to 3932.15) | 3148.61(2307.03 to 4157.44) |  | 0.21(0.2 to 0.23) |
| Samoa | 1.31(0.96 to 1.73) | 2.34(1.76 to 3.09) |  | 78.58 |  | 3507.65(2639.08 to 4460.99) | 3649.56(2730.13 to 4668.44) |  | 0.21(0.2 to 0.23) |
| San Marino | 0.25(0.18 to 0.33) | 0.55(0.39 to 0.74) |  | 120.07 |  | 1520.06(1105.59 to 2021.27) | 1613.71(1164.73 to 2171.23) |  | 0.13(0.12 to 0.15) |
| Sao Tome and Principe | 0.62(0.45 to 0.84) | 1.05(0.78 to 1.43) |  | 67.81 |  | 2095(1529.67 to 2800.15) | 2146.59(1588.11 to 2835.85) |  | 0.06(0.05 to 0.07) |
| Saudi Arabia | 62.24(45.84 to 83.57) | 217.11(162.43 to 289.33) |  | 248.84 |  | 2083.16(1548.71 to 2788.78) | 2194.29(1639.16 to 2943.69) |  | 0.17(0.16 to 0.17) |
| Senegal | 33.16(24.39 to 44.14) | 76.33(56.77 to 100.76) |  | 130.19 |  | 2110.91(1571.67 to 2766.51) | 2164.74(1623.21 to 2857.06) |  | 0.08(0.06 to 0.09) |
| Serbia | 170.62(142.81 to 203.57) | 276.92(232.11 to 330.95) |  | 62.30 |  | 3376.44(2862.06 to 3957.46) | 3584.61(3026.66 to 4259.29) |  | 0.18(0.17 to 0.19) |
| Seychelles | 0.98(0.72 to 1.27) | 2.32(1.71 to 3.04) |  | 137.64 |  | 4012.49(2966.95 to 5192.04) | 4277.47(3198.11 to 5547.98) |  | 0.18(0.16 to 0.2) |
| Sierra Leone | 21.13(15.27 to 27.75) | 37.15(27.38 to 50.19) |  | 75.82 |  | 2053.88(1486.71 to 2688.71) | 2100.65(1538.73 to 2787.95) |  | 0.06(0.05 to 0.07) |
| Singapore | 9.72(6.95 to 13.27) | 44.71(31.52 to 61.28) |  | 359.96 |  | 1021.58(732.72 to 1375.1) | 1027.45(728.46 to 1393.01) |  | 0.03(0.01 to 0.05) |
| Slovakia | 86.2(77.41 to 96.58) | 148.21(132.87 to 166.25) |  | 71.94 |  | 3383.69(3038.06 to 3775.26) | 3501.07(3143.72 to 3897.94) |  | 0.1(0.1 to 0.11) |
| Slovenia | 21.89(19.03 to 25.3) | 48.48(41.9 to 56.71) |  | 121.44 |  | 2309.57(2014.09 to 2664.56) | 2398.79(2083.14 to 2793.39) |  | 0.09(0.07 to 0.11) |
| Solomon Islands | 2.22(1.63 to 2.98) | 5.19(3.87 to 6.77) |  | 133.38 |  | 3452.88(2549.45 to 4464.43) | 3635.65(2737.7 to 4757.93) |  | 0.18(0.12 to 0.24) |
| Somalia | 20.72(15.19 to 28.28) | 49.15(35.64 to 66.09) |  | 137.22 |  | 2260.56(1664.74 to 3014.85) | 2279.61(1663.79 to 3057.82) |  | 0.19(0.17 to 0.2) |
| South Africa | 255(190.62 to 342.35) | 600.71(445.72 to 820.85) |  | 135.57 |  | 3105.1(2335.88 to 4129.09) | 3200.12(2390.46 to 4235.99) |  | 0(-0.01 to 0.02) |
| South Sudan | 33.53(24.61 to 45.87) | 41.24(30.26 to 54.8) |  | 23.00 |  | 2265.99(1687.57 to 3031.84) | 2271.98(1673.58 to 2985.56) |  | 0.09(0.07 to 0.12) |
| Spain | 294.01(212.32 to 392.47) | 657.38(474.66 to 885.52) |  | 123.59 |  | 1196.38(867.87 to 1582.32) | 1562.77(1133.62 to 2126.21) |  | -0.01(-0.03 to 0.01) |
| Sri Lanka | 205.19(151.5 to 270.53) | 524.08(389.33 to 684.31) |  | 155.41 |  | 4033.16(3013.77 to 5190.53) | 4239.44(3173.41 to 5473.65) |  | 0.52(0.38 to 0.67) |
| Sudan | 95.32(71.02 to 127.68) | 209.96(154.93 to 280.61) |  | 120.27 |  | 2036.93(1547.27 to 2702.73) | 2143.04(1581.28 to 2851.2) |  | 0.16(0.15 to 0.17) |
| Suriname | 3.4(2.51 to 4.53) | 8.89(6.54 to 11.85) |  | 161.14 |  | 2923.07(2161.95 to 3841.52) | 3142.45(2332.63 to 4138.16) |  | 0.13(0.11 to 0.14) |
| Sweden | 155.36(112.09 to 210.29) | 206.52(149.99 to 274.31) |  | 32.93 |  | 2288.83(1655.52 to 3090.18) | 2029.54(1458.74 to 2700.24) |  | 0.28(0.26 to 0.31) |
| Switzerland | 208.25(188.76 to 229.73) | 407.33(372.19 to 446.5) |  | 95.60 |  | 4726.04(4284.23 to 5211.51) | 4899.09(4458.32 to 5389.64) |  | -0.12(-0.22 to -0.03) |
| Syrian Arab Republic | 52.39(39.58 to 69.08) | 143.35(105.31 to 192.96) |  | 173.64 |  | 2043.39(1533.9 to 2695.19) | 2137.04(1576.63 to 2860.55) |  | 0.12(0.11 to 0.13) |
| Taiwan (Province of China) | 301.96(227.81 to 402.22) | 807.88(643.27 to 1012.26) |  | 167.55 |  | 3905.79(2994.36 to 4970.45) | 4038.07(3233.87 to 5014.75) |  | 0.1(0.09 to 0.12) |
| Tajikistan | 29.88(21.98 to 39.39) | 72.47(54.16 to 97.25) |  | 142.51 |  | 2738.47(2036.24 to 3545.05) | 2819.77(2117.74 to 3660.73) |  | 0.09(0.09 to 0.1) |
| Thailand | 595.31(443.36 to 772.28) | 2060.3(1539.62 to 2676.25) |  | 246.09 |  | 3970.53(2976.85 to 5076.97) | 4075.53(3070.47 to 5256.3) |  | 0.09(0.07 to 0.11) |
| Timor-Leste | 4.62(3.47 to 6.09) | 17.19(12.66 to 22.32) |  | 272.51 |  | 3953.25(2992.9 to 5105.28) | 4109.16(3066.24 to 5290.28) |  | 0.02(-0.01 to 0.04) |
| Togo | 11.12(8.27 to 14.96) | 31.41(23.08 to 43.27) |  | 182.57 |  | 2070.77(1543.73 to 2745.86) | 2114.63(1560.11 to 2814.75) |  | 0.18(0.15 to 0.21) |
| Tokelau | 0.02(0.02 to 0.03) | 0.03(0.02 to 0.03) |  | 12.39 |  | 3490.71(2599.16 to 4486) | 3640.55(2771.98 to 4642.61) |  | 0.08(0.07 to 0.1) |
| Tonga | 0.87(0.66 to 1.13) | 1.29(0.96 to 1.66) |  | 47.29 |  | 3489.33(2643.41 to 4457.38) | 3630.14(2699.78 to 4692.4) |  | 0.03(0.02 to 0.04) |
| Trinidad and Tobago | 11.91(8.63 to 15.97) | 30.3(21.62 to 40.61) |  | 154.51 |  | 3023.49(2221.68 to 4027.9) | 3166.1(2283.82 to 4180.11) |  | 0.16(0.15 to 0.18) |
| Tunisia | 52.9(38.33 to 72.95) | 139.9(102.19 to 183.81) |  | 164.44 |  | 2055.6(1515.19 to 2772.24) | 2154.95(1592.34 to 2806.22) |  | 0.14(0.12 to 0.15) |
| Turkey | 312.29(236.21 to 419.88) | 917.72(669.39 to 1220.31) |  | 193.87 |  | 2036.93(1547.27 to 2702.73) | 2091.05(1536.64 to 2758.11) |  | 0.16(0.14 to 0.18) |
| Turkmenistan | 19.32(14.2 to 25.55) | 44.92(33.04 to 58.84) |  | 132.54 |  | 2738.47(2036.24 to 3545.05) | 2793.29(2101.51 to 3615.48) |  | 0.14(0.13 to 0.15) |
| Tuvalu | 0.09(0.07 to 0.12) | 0.16(0.12 to 0.22) |  | 79.36 |  | 3485.31(2604.99 to 4498.62) | 3670.43(2770.11 to 4736.52) |  | 0.11(0.09 to 0.13) |
| Uganda | 66.94(49.02 to 90.92) | 132.36(97.4 to 179.67) |  | 97.73 |  | 2274.19(1688.92 to 3029.54) | 2299.3(1699.1 to 3071.67) |  | 0.07(0.05 to 0.09) |
| Ukraine | 1577.27(1203.46 to 2053.06) | 1898.4(1436.89 to 2419.13) |  | 20.36 |  | 6330.83(4896.36 to 7973.98) | 6314.19(4833.19 to 7938.88) |  | 0.18(0.16 to 0.19) |
| United Arab Emirates | 4.44(3.26 to 5.84) | 65.71(47.7 to 89.84) |  | 1381.24 |  | 2114.25(1568.95 to 2832.3) | 2232.79(1642.72 to 2962.42) |  | -0.01(-0.02 to 0.01) |
| United Kingdom | 950.83(782.88 to 1163.66) | 1520.04(1264.89 to 1824.2) |  | 59.86 |  | 2412.94(1992.56 to 2935.9) | 2496.13(2089.47 to 2993.24) |  | 0(-0.02 to 0.02) |
| United Republic of Tanzania | 114.01(83.34 to 153.8) | 256.87(189.35 to 344.46) |  | 125.29 |  | 2247.29(1677.31 to 2991.15) | 2271.72(1678.94 to 3036.72) |  | 0.15(0.13 to 0.17) |
| United States of America | 2476.24(2024.27 to 3030.86) | 4930.33(4358.83 to 5584.99) |  | 99.11 |  | 1840.17(1508.84 to 2218.58) | 1818.02(1616.17 to 2037.06) |  | 0.31(0.25 to 0.37) |
| United States Virgin Islands | 1.05(0.77 to 1.4) | 2.85(2.1 to 3.8) |  | 170.56 |  | 2886.03(2129.02 to 3760.65) | 3082.64(2291.67 to 4071.92) |  | 0.01(0 to 0.03) |
| Uruguay | 21.19(15.39 to 28.48) | 29.95(22.02 to 40.11) |  | 41.36 |  | 1209.81(893.58 to 1614.83) | 1283.84(943.86 to 1720.11) |  | 0.24(0.17 to 0.32) |
| Uzbekistan | 118.17(86.28 to 154.64) | 308.23(227.53 to 405.36) |  | 160.84 |  | 2726.19(2029.57 to 3549.99) | 2817.33(2109.29 to 3686.56) |  | 0.24(0.22 to 0.27) |
| Vanuatu | 0.99(0.74 to 1.32) | 2.64(1.94 to 3.45) |  | 165.35 |  | 3476.8(2608.16 to 4469.47) | 3620.78(2687.35 to 4651.39) |  | 0.27(0.21 to 0.33) |
| Venezuela (Bolivarian Republic of) | 179.78(133.25 to 232.72) | 618.82(455.39 to 809.86) |  | 244.20 |  | 4321.39(3214.26 to 5580.83) | 4546.8(3383.5 to 5881.63) |  | 0.09(0.07 to 0.11) |
| Viet Nam | 694.75(520.02 to 912.49) | 1969.44(1498.75 to 2482.84) |  | 183.47 |  | 4270.15(3220.69 to 5510.4) | 4779.66(3723.11 to 5937.78) |  | 0.16(0.14 to 0.18) |
| Yemen | 43.19(31.39 to 59.02) | 131.05(96.86 to 175) |  | 203.39 |  | 2021.03(1464.23 to 2663.22) | 2079(1543.83 to 2749.75) |  | 0.07(0.07 to 0.08) |
| Zambia | 31.28(23.03 to 41.76) | 65.81(48.4 to 88.91) |  | 110.36 |  | 2261.72(1685.63 to 3006.59) | 2283.22(1689.84 to 3034.82) |  | 0(-0.01 to 0.01) |
| Zimbabwe | 53.96(39.33 to 74.51) | 77.41(57.77 to 104.99) |  | 43.44 |  | 2834.54(2102.62 to 3821.33) | 2946.05(2193.94 to 3897.75) |  | 0.11(0.1 to 0.13) |

EAPC: estimated annual percentage change; UI: uncertainty interval; CI: confidence interval.
