# Supplementary material for: Global, regional, and national burden of benign prostatic hyperplasia from 1990 to 2021 and projection to 2035
Source: BMC Urol. 2025 Feb 19;25:34. doi: 10.1186/s12894-025-01715-9 (PMC11837592; doi:10.1186/s12894-025-01715-9)
Supplement: Supplementary file 3 — Supplementary Material 3 [file 12894_2025_1715_MOESM3_ESM.docx]

**Table S3** DALYs and age-standardized DALYs rate of benign prostatic hyperplasia in 204 countries and territories between 1990 and 2021

|  | All ages, No. ×10^3^ (95% UI) | |  | Change (%) |  | Age-standardized rate per 100,000, No. (95% UI) | |  | EAPC (95% CI) |
| --- | --- | --- | --- | --- | --- | --- | --- | --- | --- |
| Location | **1990** | **2021** |  | **1990–2021** |  | **1990** | **2021** |  | **1990–2021** |
| Afghanistan | 1.48(0.86 to 2.31) | 1.5(0.89 to 2.36) |  | 1.19 |  | 40.43(23.72 to 63.58) | 41.79(24.83 to 65.73) |  | 0.1(0.09 to 0.11) |
| Albania | 0.55(0.32 to 0.86) | 1.37(0.81 to 2.09) |  | 150.79 |  | 58.88(34.69 to 90.8) | 61.77(36.73 to 94.69) |  | 0.13(0.11 to 0.15) |
| Algeria | 2.43(1.39 to 3.94) | 7.58(4.52 to 11.67) |  | 211.94 |  | 40.67(23.51 to 64.82) | 42.61(25.41 to 65.02) |  | 0.12(0.11 to 0.14) |
| American Samoa | 0.01(0 to 0.01) | 0.02(0.01 to 0.03) |  | 149.37 |  | 72.42(42.83 to 109.83) | 77(45 to 117.91) |  | 0.22(0.2 to 0.24) |
| Andorra | 0.01(0.01 to 0.01) | 0.03(0.01 to 0.04) |  | 176.77 |  | 30.4(18.6 to 47.94) | 32.34(18.87 to 50.39) |  | 0.28(0.25 to 0.31) |
| Angola | 0.72(0.43 to 1.13) | 2.01(1.21 to 3.15) |  | 179.79 |  | 41.44(24.35 to 66.03) | 42.3(25.35 to 65.97) |  | 0.05(0.04 to 0.06) |
| Antigua and Barbuda | 0.01(0.01 to 0.02) | 0.03(0.02 to 0.05) |  | 127.03 |  | 57.93(34.86 to 91.46) | 61.02(36.42 to 96.87) |  | 0.19(0.18 to 0.21) |
| Argentina | 3.53(2.1 to 5.51) | 6.47(3.73 to 10.17) |  | 82.94 |  | 24.52(14.57 to 37.95) | 25.99(15.03 to 40.81) |  | 0.24(0.2 to 0.29) |
| Armenia | 0.59(0.35 to 0.9) | 1.05(0.62 to 1.64) |  | 78.18 |  | 54.74(32.41 to 84.54) | 56.14(33.21 to 86.24) |  | 0.09(0.06 to 0.11) |
| Australia | 3.33(1.99 to 5.33) | 8.47(4.96 to 13.31) |  | 154.27 |  | 36.82(21.96 to 58.39) | 38.38(22.55 to 59.83) |  | 0.12(0.1 to 0.14) |
| Austria | 3.2(1.92 to 5.03) | 7.47(4.69 to 10.85) |  | 132.95 |  | 69.81(41.78 to 109.87) | 92.22(57.66 to 133.9) |  | 0.58(0.46 to 0.7) |
| Azerbaijan | 1.01(0.6 to 1.55) | 2.46(1.43 to 3.83) |  | 142.42 |  | 55.23(32.57 to 86.99) | 56.52(33.86 to 86.91) |  | 0.07(0.05 to 0.09) |
| Bahamas | 0.04(0.02 to 0.06) | 0.11(0.06 to 0.17) |  | 197.65 |  | 58.48(34.58 to 94.27) | 60.94(36.76 to 97.73) |  | 0.2(0.18 to 0.21) |
| Bahrain | 0.04(0.02 to 0.06) | 0.24(0.14 to 0.37) |  | 569.93 |  | 41.99(25.23 to 65.21) | 45.1(26.91 to 69.86) |  | 0.14(0.13 to 0.14) |
| Bangladesh | 13.56(7.94 to 20.8) | 40.94(24.1 to 64.15) |  | 202.04 |  | 55.23(32.37 to 85.53) | 57.38(33.7 to 89.49) |  | 0.16(0.14 to 0.18) |
| Barbados | 0.07(0.04 to 0.12) | 0.15(0.09 to 0.24) |  | 100.98 |  | 58.55(35.01 to 91.44) | 60.92(36.98 to 95.58) |  | 0(-0.02 to 0.02) |
| Belarus | 5.29(3.21 to 8.07) | 7.08(4.27 to 10.78) |  | 33.79 |  | 116.4(70.37 to 177.16) | 115.36(69.92 to 175.84) |  | 0.3(0.26 to 0.35) |
| Belgium | 5.09(2.64 to 7.89) | 8.71(4.75 to 13.13) |  | 70.95 |  | 76.49(39.69 to 118.44) | 81.82(44.52 to 123.86) |  | 0.19(0.17 to 0.21) |
| Belize | 0.03(0.02 to 0.04) | 0.09(0.05 to 0.13) |  | 232.16 |  | 57.3(34.04 to 90.76) | 60.23(35.17 to 94.8) |  | 0.07(0.06 to 0.08) |
| Benin | 0.38(0.22 to 0.6) | 0.95(0.56 to 1.47) |  | 146.45 |  | 41.44(24.04 to 64.96) | 42.53(25.12 to 67.29) |  | 0.15(0.13 to 0.16) |
| Bermuda | 0.02(0.01 to 0.02) | 0.04(0.02 to 0.06) |  | 146.66 |  | 57.12(34.1 to 90.53) | 59.38(35.04 to 93.14) |  | 0.15(0.14 to 0.16) |
| Bhutan | 0.06(0.04 to 0.09) | 0.17(0.1 to 0.27) |  | 183.66 |  | 55.3(32.27 to 84.92) | 57.97(34.55 to 88.9) |  | 0.16(0.15 to 0.18) |
| Bolivia (Plurinational State of) | 0.76(0.45 to 1.23) | 2.4(1.41 to 3.82) |  | 215.62 |  | 54.35(32.14 to 86.95) | 57.31(33.5 to 90.36) |  | 0.22(0.19 to 0.25) |
| Bosnia and Herzegovina | 0.97(0.58 to 1.53) | 1.81(1.07 to 2.8) |  | 86.05 |  | 59.41(35.11 to 92.29) | 63.74(37.64 to 98.28) |  | 0.08(0.07 to 0.1) |
| Botswana | 0.16(0.1 to 0.25) | 0.41(0.25 to 0.63) |  | 153.80 |  | 67.01(40.49 to 104.61) | 68.21(41.14 to 104.4) |  | -0.32(-0.44 to -0.2) |
| Brazil | 18.01(11.41 to 27.01) | 41.59(26.44 to 62.97) |  | 130.97 |  | 45.66(28.79 to 68.57) | 37.01(23.48 to 55.93) |  | 0.12(0.1 to 0.14) |
| Brunei Darussalam | 0.01(0.01 to 0.02) | 0.04(0.02 to 0.06) |  | 260.55 |  | 21.26(12.31 to 33.81) | 22.07(12.76 to 34.82) |  | 0.14(0.11 to 0.17) |
| Bulgaria | 3.48(2.1 to 5.44) | 3.95(2.37 to 6.12) |  | 13.32 |  | 59.3(35.76 to 92.07) | 62.61(37.13 to 97.12) |  | 0.08(0.07 to 0.09) |
| Burkina Faso | 0.84(0.5 to 1.3) | 1.76(1.06 to 2.77) |  | 110.06 |  | 41.49(24.94 to 63.91) | 42.66(25.95 to 67.3) |  | -0.01(-0.03 to 0.01) |
| Burundi | 0.45(0.28 to 0.71) | 1.06(0.63 to 1.66) |  | 133.58 |  | 45.33(27.87 to 71.16) | 45.28(27.12 to 71.09) |  | 0.11(0.1 to 0.12) |
| Cabo Verde | 0.04(0.02 to 0.07) | 0.08(0.05 to 0.12) |  | 83.56 |  | 41.99(24.9 to 66.2) | 43.07(25.73 to 67.8) |  | 0.08(0.07 to 0.09) |
| Cambodia | 1.37(0.82 to 2.14) | 3.93(2.34 to 6.1) |  | 186.45 |  | 78.46(46.7 to 121.93) | 81.26(48.52 to 124.84) |  | 0.22(0.18 to 0.26) |
| Cameroon | 0.85(0.51 to 1.31) | 2.38(1.4 to 3.62) |  | 180.47 |  | 41.45(25.07 to 64.46) | 42.52(25.2 to 64.75) |  | 0.06(0.05 to 0.07) |
| Canada | 5(3.02 to 7.88) | 12.36(7.29 to 19.11) |  | 147.05 |  | 34.88(21.1 to 54.63) | 36.44(21.25 to 56.11) |  | 0.05(0.04 to 0.06) |
| Central African Republic | 0.2(0.12 to 0.31) | 0.37(0.22 to 0.57) |  | 85.81 |  | 41.11(24.25 to 63.45) | 42.24(25.28 to 67.56) |  | 0.27(0.24 to 0.31) |
| Chad | 0.56(0.33 to 0.88) | 1.25(0.73 to 1.91) |  | 122.25 |  | 41.62(24.5 to 64.62) | 42.49(25.17 to 64.24) |  | -0.26(-0.43 to -0.09) |
| Chile | 1.35(0.81 to 2.14) | 3.85(2.31 to 6.07) |  | 185.25 |  | 30.6(18.48 to 48.11) | 32.65(19.72 to 51.56) |  | 0.16(0.15 to 0.17) |
| China | 199.95(115.45 to 307.32) | 460.19(270.75 to 701.43) |  | 130.15 |  | 57.33(33.25 to 88) | 45.84(27.32 to 69.48) |  | 0.16(0.14 to 0.17) |
| Colombia | 6.73(4.1 to 10.54) | 22.13(13.59 to 34.23) |  | 228.75 |  | 85.67(52.6 to 132.81) | 89.81(55.28 to 137.96) |  | 0(-0.01 to 0.01) |
| Comoros | 0.04(0.02 to 0.07) | 0.1(0.06 to 0.15) |  | 132.72 |  | 45(26.99 to 71.19) | 45.13(26.51 to 71.35) |  | 0.03(0.02 to 0.05) |
| Congo | 0.18(0.11 to 0.29) | 0.51(0.3 to 0.79) |  | 175.23 |  | 41.94(24.68 to 66.06) | 42.77(25.39 to 67.48) |  | 0.16(0.15 to 0.18) |
| Cook Islands | 0(0 to 0.01) | 0.01(0.01 to 0.01) |  | 123.21 |  | 69.38(41.59 to 106.63) | 72.36(42.48 to 110.12) |  | 0.16(0.15 to 0.17) |
| Costa Rica | 0.69(0.42 to 1.08) | 2.26(1.37 to 3.49) |  | 226.69 |  | 85.9(51.92 to 133) | 89.92(54.46 to 139.23) |  | 0.02(-0.06 to 0.1) |
| Croatia | 1.53(0.96 to 2.25) | 2.42(1.53 to 3.52) |  | 57.63 |  | 64.39(40.39 to 94.87) | 60.51(38.04 to 87.94) |  | 0.15(0.13 to 0.17) |
| Cuba | 2.98(1.79 to 4.73) | 5.6(3.37 to 8.88) |  | 87.94 |  | 58.65(35.11 to 92.84) | 60.81(36.52 to 96.53) |  | 0.12(0.11 to 0.14) |
| Cyprus | 0.12(0.08 to 0.18) | 0.32(0.21 to 0.49) |  | 164.20 |  | 30(18.68 to 44.94) | 31.19(19.96 to 46.79) |  | 0.23(0.21 to 0.26) |
| Czechia | 4.31(2.68 to 6.45) | 8.05(4.9 to 12.3) |  | 86.98 |  | 75.58(47.04 to 112.97) | 82.27(50.54 to 124.31) |  | 0.1(0.09 to 0.11) |
| Cote d'Ivoire | 0.79(0.46 to 1.23) | 2.26(1.34 to 3.49) |  | 185.67 |  | 41.02(24.27 to 63.82) | 42.51(25.1 to 66.5) |  | 0.06(0.05 to 0.07) |
| Democratic People's Republic of Korea | 3.05(1.79 to 4.82) | 7.77(4.47 to 12.08) |  | 154.71 |  | 64.46(36.92 to 101.18) | 66.03(38.54 to 103.13) |  | 0.34(0.29 to 0.38) |
| Democratic Republic of the Congo | 2.81(1.64 to 4.34) | 6.34(3.72 to 9.73) |  | 126.00 |  | 41.34(24.37 to 64.35) | 42.21(25.15 to 65.7) |  | 0.01(-0.01 to 0.02) |
| Denmark | 1.15(0.64 to 1.91) | 1.96(1.07 to 3.26) |  | 70.04 |  | 32.32(17.92 to 53.28) | 34.47(19.04 to 56.9) |  | 0.23(0.21 to 0.25) |
| Djibouti | 0.03(0.02 to 0.04) | 0.14(0.08 to 0.23) |  | 429.85 |  | 45.16(26.78 to 69.52) | 45.7(27.07 to 70.36) |  | 0.17(0.14 to 0.19) |
| Dominica | 0.01(0.01 to 0.02) | 0.03(0.02 to 0.04) |  | 81.58 |  | 58.56(35.47 to 91.56) | 62.24(37.29 to 99.52) |  | -0.01(-0.12 to 0.09) |
| Dominican Republic | 0.99(0.58 to 1.6) | 2.73(1.64 to 4.32) |  | 177.06 |  | 56.64(33.56 to 91.92) | 58.85(35.16 to 93.07) |  | 0.11(0.1 to 0.12) |
| Ecuador | 1.47(0.85 to 2.37) | 4.44(2.59 to 7.11) |  | 201.46 |  | 60.18(34.76 to 96.92) | 57.57(33.56 to 92.31) |  | 0.22(0.22 to 0.23) |
| Egypt | 5.11(3.03 to 7.97) | 13.51(7.99 to 21.43) |  | 164.11 |  | 40.21(23.9 to 62.7) | 41.85(25.27 to 66.82) |  | 0.09(0.08 to 0.1) |
| El Salvador | 1.11(0.68 to 1.76) | 2.3(1.4 to 3.59) |  | 107.58 |  | 84.47(51.24 to 133.67) | 89.71(54.86 to 141.22) |  | -0.01(-0.02 to 0.01) |
| Equatorial Guinea | 0.03(0.02 to 0.05) | 0.08(0.05 to 0.13) |  | 146.97 |  | 41.05(24.45 to 65.47) | 42.53(25.41 to 67.55) |  | 0.06(0.03 to 0.08) |
| Eritrea | 0.18(0.1 to 0.27) | 0.44(0.26 to 0.67) |  | 149.12 |  | 44.58(26.66 to 70.82) | 44.95(26.75 to 69.61) |  | 0(-0.02 to 0.02) |
| Estonia | 0.83(0.51 to 1.29) | 1.2(0.73 to 1.82) |  | 44.51 |  | 116.21(71.07 to 178.62) | 117.18(71.41 to 177.66) |  | 0.21(0.18 to 0.23) |
| Eswatini | 0.07(0.04 to 0.1) | 0.12(0.07 to 0.18) |  | 80.09 |  | 56.75(34.47 to 87.7) | 57.73(34.66 to 89.02) |  | 0.2(0.19 to 0.21) |
| Ethiopia | 4.75(2.87 to 7.41) | 9.96(5.99 to 15.59) |  | 109.80 |  | 48.53(29.36 to 76.45) | 48.76(29.28 to 77.15) |  | 0.23(0.21 to 0.24) |
| Fiji | 0.1(0.06 to 0.16) | 0.24(0.14 to 0.36) |  | 131.33 |  | 70.34(42.04 to 108.53) | 74.02(43.97 to 113.68) |  | 0.27(0.23 to 0.3) |
| Finland | 2.13(1.33 to 3.1) | 4.84(3.07 to 6.96) |  | 127.52 |  | 75.99(47.4 to 110.35) | 80.39(50.79 to 114.77) |  | 0.05(0.03 to 0.06) |
| France | 10.52(6.23 to 16.89) | 19.31(11.8 to 30.73) |  | 83.60 |  | 29.95(17.77 to 47.89) | 31.65(19.24 to 50.7) |  | 0.1(0.03 to 0.17) |
| Gabon | 0.11(0.06 to 0.16) | 0.2(0.12 to 0.32) |  | 93.93 |  | 41.77(25.36 to 65.38) | 42.69(25.44 to 66.47) |  | 0.33(0.3 to 0.37) |
| Gambia | 0.07(0.04 to 0.11) | 0.19(0.11 to 0.29) |  | 156.93 |  | 41.79(24.63 to 64.21) | 42.44(25.13 to 66.74) |  | 0.08(0.07 to 0.08) |
| Georgia | 1.4(0.83 to 2.16) | 1.57(1 to 2.31) |  | 12.06 |  | 59.41(34.69 to 91.53) | 64.31(41.02 to 94.38) |  | 0.2(0.1 to 0.29) |
| Germany | 15.05(8.83 to 23.78) | 28.65(16.85 to 46.16) |  | 90.36 |  | 30.73(18.11 to 48.93) | 33.06(19.6 to 53.15) |  | 0.29(0.25 to 0.33) |
| Ghana | 1.17(0.72 to 1.83) | 2.91(1.75 to 4.51) |  | 149.38 |  | 41.88(25.27 to 65.41) | 42.86(25.51 to 66.29) |  | 0.2(0.18 to 0.22) |
| Greece | 2.2(1.31 to 3.5) | 3.67(2.23 to 5.56) |  | 66.79 |  | 30.49(18.07 to 48.23) | 35.13(21.52 to 53.51) |  | 0.09(0.08 to 0.11) |
| Greenland | 0.01(0 to 0.01) | 0.01(0.01 to 0.02) |  | 168.69 |  | 34.42(20.08 to 54.26) | 36.57(21.59 to 58.25) |  | 0.23(0.22 to 0.24) |
| Grenada | 0.02(0.01 to 0.03) | 0.03(0.02 to 0.05) |  | 87.35 |  | 58.33(34.77 to 93.21) | 61.68(36.93 to 95.9) |  | 0.06(0.04 to 0.07) |
| Guam | 0.02(0.01 to 0.03) | 0.07(0.04 to 0.11) |  | 208.53 |  | 68.73(40.88 to 107.7) | 70.36(40.9 to 107.96) |  | 0.07(0.06 to 0.08) |
| Guatemala | 1.35(0.82 to 2.1) | 4.5(2.73 to 6.93) |  | 234.09 |  | 84.66(51.6 to 132.88) | 89.95(54.6 to 138.34) |  | 0.23(0.21 to 0.25) |
| Guinea | 0.68(0.4 to 1.09) | 1.17(0.68 to 1.78) |  | 70.97 |  | 41.58(24.78 to 66.37) | 42.73(25.04 to 64.7) |  | 0.22(0.21 to 0.23) |
| Guinea-Bissau | 0.08(0.04 to 0.12) | 0.13(0.07 to 0.19) |  | 66.20 |  | 41.38(24.33 to 65.21) | 42.56(25.01 to 65.99) |  | 0.17(0.16 to 0.18) |
| Guyana | 0.1(0.06 to 0.16) | 0.18(0.11 to 0.28) |  | 81.65 |  | 58.32(34.47 to 92.14) | 61.79(37.11 to 97.07) |  | 0.18(0.16 to 0.21) |
| Haiti | 0.84(0.5 to 1.34) | 1.89(1.11 to 3.03) |  | 126.40 |  | 57.67(33.95 to 91.35) | 61.15(35.7 to 97.56) |  | 0.34(0.29 to 0.38) |
| Honduras | 0.8(0.49 to 1.25) | 2.56(1.56 to 4) |  | 218.74 |  | 86.29(52.29 to 133.4) | 90.48(54.95 to 139.55) |  | 0.24(0.21 to 0.28) |
| Hungary | 3.63(2.18 to 5.5) | 5.15(3.07 to 8.03) |  | 41.94 |  | 59.2(35.42 to 89.16) | 63.14(37.95 to 97.67) |  | -0.43(-0.7 to -0.17) |
| Iceland | 0.04(0.02 to 0.06) | 0.09(0.05 to 0.14) |  | 129.22 |  | 29.2(17.37 to 45.36) | 31.18(18.46 to 49.21) |  | 0.06(0.04 to 0.08) |
| India | 154.45(92.62 to 233.62) | 432.53(259.93 to 666.84) |  | 180.05 |  | 69.65(41.68 to 106.76) | 77.46(46.66 to 118.5) |  | 0.33(0.29 to 0.37) |
| Indonesia | 48.02(29.37 to 73.9) | 87.54(52.87 to 134.77) |  | 82.31 |  | 111.43(67.22 to 169.27) | 78.62(48.34 to 120.83) |  | 0.11(0.1 to 0.13) |
| Iran (Islamic Republic of) | 5.82(3.46 to 9.13) | 16.85(9.95 to 26.14) |  | 189.63 |  | 43.95(26.17 to 69.41) | 45.76(27.06 to 71.87) |  | 0.29(0.26 to 0.32) |
| Iraq | 1.46(0.86 to 2.31) | 4.57(2.74 to 7.08) |  | 212.47 |  | 40.92(24.26 to 64.97) | 42.16(24.5 to 64.66) |  | 0.06(0.02 to 0.1) |
| Ireland | 0.58(0.34 to 0.91) | 1.24(0.72 to 1.94) |  | 115.00 |  | 30.12(17.83 to 47.35) | 32.34(18.85 to 50.18) |  | 0.23(0.21 to 0.24) |
| Israel | 0.69(0.4 to 1.09) | 1.86(1.08 to 2.91) |  | 168.90 |  | 30.55(18.01 to 48.06) | 32.61(19.03 to 50.93) |  | -0.08(-0.17 to 0) |
| Italy | 26.61(16.64 to 40) | 39.95(25.18 to 58.94) |  | 50.10 |  | 67.1(41.99 to 100.62) | 66.56(41.56 to 97.77) |  | 0.21(0.11 to 0.31) |
| Jamaica | 0.49(0.29 to 0.79) | 0.9(0.54 to 1.43) |  | 84.75 |  | 57.88(34.79 to 93.41) | 61.38(36.81 to 98.38) |  | 0.07(0.05 to 0.09) |
| Japan | 20.17(11.94 to 31.96) | 38.08(23.59 to 59.37) |  | 88.76 |  | 26.82(15.88 to 42.67) | 24.51(15.03 to 38.4) |  | 0(0 to 0.01) |
| Jordan | 0.25(0.15 to 0.39) | 1.77(1.06 to 2.76) |  | 602.90 |  | 41.28(24.52 to 65.62) | 48.11(29.13 to 74.04) |  | 0.04(0.03 to 0.06) |
| Kazakhstan | 2.35(1.37 to 3.62) | 3.92(2.36 to 6.02) |  | 66.48 |  | 54.9(32.23 to 86.05) | 56.42(33.77 to 85.81) |  | 0.18(0.18 to 0.19) |
| Kenya | 2.19(1.29 to 3.42) | 5.78(3.44 to 8.99) |  | 163.15 |  | 58.25(34.26 to 90.06) | 58.55(34.35 to 91.24) |  | 0.08(0.05 to 0.1) |
| Kiribati | 0.01(0.01 to 0.01) | 0.02(0.01 to 0.03) |  | 94.96 |  | 69.22(40.26 to 105.49) | 72.76(43.1 to 110.21) |  | 0.01(-0.01 to 0.04) |
| Kuwait | 0.13(0.08 to 0.2) | 0.66(0.39 to 1.03) |  | 399.83 |  | 41.71(24.95 to 65.35) | 43.36(25.45 to 67.75) |  | 0.11(0.09 to 0.12) |
| Kyrgyzstan | 0.52(0.3 to 0.82) | 0.92(0.55 to 1.45) |  | 78.58 |  | 47.91(28.28 to 75.74) | 48.28(28.24 to 75.51) |  | 0.08(0.06 to 0.1) |
| Lao People's Democratic Republic | 0.74(0.45 to 1.16) | 1.7(1.01 to 2.65) |  | 129.43 |  | 79.77(48.38 to 123.36) | 82.59(49.2 to 127.23) |  | 0.09(0.08 to 0.1) |
| Latvia | 1.44(0.87 to 2.2) | 1.71(1.03 to 2.6) |  | 18.56 |  | 115.34(68.97 to 172.89) | 116.39(69.97 to 176.67) |  | 0.03(0.02 to 0.04) |
| Lebanon | 0.42(0.25 to 0.64) | 1.13(0.67 to 1.73) |  | 170.75 |  | 40.6(23.92 to 62.16) | 41.98(24.89 to 64.62) |  | 0.08(0.06 to 0.09) |
| Lesotho | 0.17(0.1 to 0.26) | 0.22(0.13 to 0.35) |  | 34.54 |  | 56.53(34.5 to 88.64) | 57.5(33.55 to 90.25) |  | 0.14(0.13 to 0.15) |
| Liberia | 0.26(0.16 to 0.41) | 0.41(0.24 to 0.65) |  | 60.51 |  | 41.65(25.06 to 65.6) | 42.52(25.09 to 66.02) |  | 0.05(0.04 to 0.06) |
| Libya | 0.38(0.22 to 0.59) | 1.03(0.61 to 1.59) |  | 171.22 |  | 41.04(24.16 to 64.87) | 43.05(25.09 to 66.97) |  | 0.49(0.44 to 0.54) |
| Lithuania | 2.21(1.39 to 3.28) | 2.87(1.82 to 4.29) |  | 29.79 |  | 131.36(82.54 to 194.27) | 132.83(83.91 to 198.87) |  | 0.01(0 to 0.02) |
| Luxembourg | 0.07(0.04 to 0.12) | 0.18(0.11 to 0.28) |  | 143.85 |  | 31.83(19.33 to 50.37) | 35.53(21.47 to 56.61) |  | 0.05(0.04 to 0.05) |
| Madagascar | 1.1(0.64 to 1.68) | 2.21(1.28 to 3.46) |  | 101.90 |  | 44.99(26.05 to 68.98) | 45.33(26.23 to 69.49) |  | 0.07(0.06 to 0.08) |
| Malawi | 0.76(0.44 to 1.19) | 1.36(0.8 to 2.1) |  | 80.24 |  | 45.15(26.48 to 70.19) | 45.63(27.1 to 70.52) |  | 0.06(0.05 to 0.07) |
| Malaysia | 3.26(1.96 to 5.1) | 11.46(6.72 to 17.78) |  | 251.47 |  | 80.12(47.44 to 125.11) | 82.57(48.28 to 127.78) |  | 0.05(0.04 to 0.06) |
| Maldives | 0.04(0.02 to 0.06) | 0.13(0.08 to 0.2) |  | 240.75 |  | 79.72(47.63 to 122.72) | 81.74(49.61 to 126.15) |  | 0.18(0.16 to 0.2) |
| Mali | 0.79(0.47 to 1.23) | 1.81(1.07 to 2.81) |  | 128.07 |  | 41.36(25.06 to 64.12) | 42.21(25.1 to 65.57) |  | 0.24(0.23 to 0.26) |
| Malta | 0.08(0.05 to 0.12) | 0.21(0.13 to 0.32) |  | 156.28 |  | 43.28(26.59 to 63.1) | 45.37(27.93 to 66.29) |  | 0.03(0.02 to 0.05) |
| Marshall Islands | 0(0 to 0.01) | 0.01(0.01 to 0.02) |  | 134.30 |  | 71.77(42.75 to 109.61) | 77.08(44.62 to 116.8) |  | 0.17(0.15 to 0.18) |
| Mauritania | 0.19(0.11 to 0.3) | 0.44(0.27 to 0.71) |  | 131.84 |  | 41.51(24.26 to 65.74) | 42.39(25.52 to 66.97) |  | 0.09(0.04 to 0.14) |
| Mauritius | 0.25(0.15 to 0.39) | 0.74(0.44 to 1.15) |  | 194.09 |  | 80.91(48.58 to 124.36) | 84.94(50.33 to 131.49) |  | 0.02(0 to 0.04) |
| Mexico | 18.9(12.03 to 28.56) | 56.72(36.17 to 85.36) |  | 200.05 |  | 99.44(63.35 to 150.84) | 98.8(62.95 to 148.96) |  | 0.14(0.12 to 0.16) |
| Micronesia (Federated States of) | 0.01(0.01 to 0.02) | 0.02(0.01 to 0.03) |  | 46.07 |  | 68.56(39.43 to 104.7) | 72.49(42.42 to 110.2) |  | 0.1(0.09 to 0.11) |
| Monaco | 0.01(0.01 to 0.01) | 0.01(0.01 to 0.02) |  | 59.90 |  | 30.42(18.14 to 47.58) | 32.45(19.22 to 50.6) |  | 0.04(0.03 to 0.05) |
| Mongolia | 0.24(0.14 to 0.36) | 0.47(0.28 to 0.74) |  | 100.09 |  | 53.62(31.62 to 82.52) | 54.15(31.59 to 83.94) |  | 0.09(0.09 to 0.1) |
| Montenegro | 0.16(0.09 to 0.25) | 0.29(0.17 to 0.44) |  | 82.11 |  | 60.3(35.23 to 94.2) | 63.53(37.73 to 96.17) |  | 0.04(0.02 to 0.05) |
| Morocco | 2.79(1.65 to 4.41) | 7.11(4.23 to 11.04) |  | 154.68 |  | 40.41(23.86 to 63.81) | 42.08(24.87 to 65.16) |  | 0.09(0.02 to 0.15) |
| Mozambique | 1.19(0.69 to 1.86) | 2.02(1.2 to 3.18) |  | 70.26 |  | 44.47(26.78 to 69.94) | 45.07(26.16 to 69.85) |  | 0.23(0.2 to 0.26) |
| Myanmar | 7.99(4.85 to 12.25) | 16.44(9.88 to 25.71) |  | 105.67 |  | 79.27(47.75 to 120.23) | 81.45(48.88 to 125.49) |  | -0.33(-0.44 to -0.21) |
| Namibia | 0.16(0.09 to 0.25) | 0.31(0.18 to 0.49) |  | 96.61 |  | 56.61(32.87 to 88.71) | 57.97(33.99 to 89.61) |  | 0.18(0.17 to 0.2) |
| Nauru | 0(0 to 0) | 0(0 to 0) |  | 1.03 |  | 68.5(41.13 to 104.61) | 71.23(42.82 to 107.97) |  | 0.06(0.04 to 0.07) |
| Nepal | 2.23(1.34 to 3.33) | 5.29(3.23 to 8) |  | 136.62 |  | 48.97(29.77 to 73.2) | 47.69(29.12 to 72.48) |  | 0.04(0.03 to 0.06) |
| Netherlands | 2.63(1.57 to 4.03) | 5.47(3.22 to 8.74) |  | 107.63 |  | 30.54(18.17 to 46.54) | 32.02(18.86 to 50.54) |  | 0.12(0.11 to 0.13) |
| New Zealand | 0.9(0.55 to 1.35) | 1.8(1.12 to 2.66) |  | 100.15 |  | 49.81(30.79 to 74.85) | 43.06(26.96 to 63.92) |  | 0.09(0.08 to 0.1) |
| Nicaragua | 0.56(0.34 to 0.88) | 1.87(1.13 to 2.88) |  | 231.74 |  | 85.69(51.9 to 131.93) | 89.97(54.6 to 137.87) |  | 0.11(0.09 to 0.13) |
| Niger | 0.56(0.33 to 0.88) | 1.59(0.94 to 2.56) |  | 186.16 |  | 41.2(24.82 to 65.53) | 41.98(25.23 to 66.25) |  | 0.16(0.15 to 0.17) |
| Nigeria | 9.76(5.7 to 15.03) | 17.71(10.56 to 27.37) |  | 81.42 |  | 45.17(26.59 to 71.2) | 45.98(27 to 72.98) |  | 0.1(0.1 to 0.11) |
| Niue | 0(0 to 0) | 0(0 to 0) |  | 10.27 |  | 70.79(42.38 to 106.06) | 75.14(44.2 to 114.25) |  | 0.18(0.17 to 0.19) |
| North Macedonia | 0.51(0.3 to 0.8) | 1.05(0.61 to 1.64) |  | 104.54 |  | 59.99(35.45 to 92.29) | 64.05(37.39 to 97.64) |  | 0.14(0.12 to 0.15) |
| Northern Mariana Islands | 0(0 to 0.01) | 0.02(0.01 to 0.03) |  | 259.13 |  | 69.25(40.49 to 108) | 71.41(42.22 to 108.69) |  | 0.11(0.09 to 0.12) |
| Norway | 3.13(1.94 to 4.69) | 5.12(3.18 to 7.69) |  | 63.78 |  | 103.18(64.14 to 153.46) | 106.03(65.62 to 158.74) |  | 0.19(0.17 to 0.21) |
| Oman | 0.13(0.08 to 0.2) | 0.41(0.24 to 0.64) |  | 218.28 |  | 40.35(23.85 to 62.8) | 42.04(24.94 to 64.99) |  | 0.06(0.03 to 0.09) |
| Pakistan | 18.51(10.88 to 28.96) | 37.59(22.27 to 57.69) |  | 103.10 |  | 60.45(35.64 to 94.04) | 63.01(37.15 to 98.33) |  | 0.18(0.16 to 0.2) |
| Palau | 0(0 to 0) | 0.01(0 to 0.01) |  | 150.09 |  | 69.48(39.81 to 105.46) | 72.78(42.65 to 110.45) |  | -0.8(-1.22 to -0.37) |
| Palestine | 0.15(0.09 to 0.24) | 0.49(0.29 to 0.77) |  | 218.58 |  | 40.92(23.61 to 63.97) | 42.41(24.87 to 65.16) |  | 0.34(0.3 to 0.38) |
| Panama | 0.62(0.38 to 0.97) | 1.91(1.16 to 2.96) |  | 209.24 |  | 85.82(52.72 to 132.95) | 90.51(55.12 to 140.18) |  | 0.29(0.26 to 0.33) |
| Papua New Guinea | 0.53(0.31 to 0.83) | 1.58(0.95 to 2.42) |  | 198.01 |  | 68.24(40.44 to 104.03) | 70.74(42.65 to 106.28) |  | 0.2(0.17 to 0.22) |
| Paraguay | 0.43(0.25 to 0.67) | 1.18(0.7 to 1.92) |  | 176.65 |  | 42.71(25.26 to 66.13) | 44.06(26.4 to 70.76) |  | 0.26(0.22 to 0.29) |
| Peru | 2.96(1.72 to 4.71) | 8.93(5.24 to 14.23) |  | 201.73 |  | 54.19(31.43 to 86.1) | 57.09(33.55 to 91.05) |  | 0.07(0.06 to 0.08) |
| Philippines | 11.14(6.63 to 17.19) | 31.47(19.19 to 48.29) |  | 182.60 |  | 85.76(50.88 to 131.32) | 89.07(53.93 to 136.38) |  | 0.09(0.08 to 0.1) |
| Poland | 15.48(9.4 to 24.04) | 15.76(10.11 to 23.43) |  | 1.76 |  | 87.26(53.06 to 133.86) | 50.85(32.56 to 74.73) |  | 0.11(0.1 to 0.12) |
| Portugal | 1.96(1.17 to 3.12) | 3.5(2.04 to 5.58) |  | 78.40 |  | 30.88(18.53 to 48.5) | 33.12(19.66 to 52.52) |  | 0.02(0.01 to 0.04) |
| Puerto Rico | 1.01(0.58 to 1.61) | 2.01(1.21 to 3.16) |  | 99.00 |  | 58.64(34 to 92.7) | 61.69(36.96 to 96.3) |  | 0.14(0.13 to 0.16) |
| Qatar | 0.03(0.02 to 0.04) | 0.26(0.16 to 0.42) |  | 917.50 |  | 42.44(25.17 to 66.96) | 45.93(27.65 to 71.67) |  | 0.22(0.21 to 0.23) |
| Republic of Korea | 2.21(1.25 to 3.57) | 9.22(5.38 to 15) |  | 317.49 |  | 19.66(11.37 to 31.28) | 20.36(11.83 to 32.59) |  | 0.17(0.16 to 0.18) |
| Republic of Moldova | 2.03(1.2 to 3.08) | 2.86(1.72 to 4.37) |  | 41.17 |  | 117.44(70.34 to 175.01) | 117.46(70.79 to 177.39) |  | 0.29(0.25 to 0.32) |
| Romania | 8.92(5.75 to 13.18) | 12.35(7.76 to 18.3) |  | 38.50 |  | 72.34(46.45 to 107.36) | 75.91(47.6 to 112.32) |  | 0.04(0.03 to 0.06) |
| Russian Federation | 73.04(44.22 to 111.4) | 114.9(69.31 to 172.7) |  | 57.31 |  | 123.83(75.55 to 187.05) | 123.78(75.52 to 186.2) |  | 0.15(0.14 to 0.16) |
| Rwanda | 0.53(0.31 to 0.85) | 1.11(0.65 to 1.72) |  | 109.74 |  | 44.98(26.55 to 70.7) | 44.95(26.1 to 70.06) |  | 0.02(0 to 0.04) |
| Saint Kitts and Nevis | 0.01(0.01 to 0.02) | 0.02(0.01 to 0.03) |  | 82.17 |  | 58.52(34.36 to 94.76) | 60.75(36.8 to 97.68) |  | 0(-0.01 to 0.01) |
| Saint Lucia | 0.02(0.01 to 0.04) | 0.07(0.04 to 0.11) |  | 203.73 |  | 59.7(35.47 to 93.36) | 62.72(37.35 to 100.12) |  | 0.14(0.13 to 0.16) |
| Saint Vincent and the Grenadines | 0.02(0.01 to 0.03) | 0.05(0.03 to 0.07) |  | 145.14 |  | 58.99(35.49 to 92.75) | 62.06(37.28 to 98.05) |  | 0.19(0.17 to 0.21) |
| Samoa | 0.03(0.02 to 0.04) | 0.05(0.03 to 0.07) |  | 77.73 |  | 69.18(40.42 to 104.58) | 71.83(42.93 to 111) |  | 0.18(0.16 to 0.19) |
| San Marino | 0(0 to 0.01) | 0.01(0.01 to 0.02) |  | 119.02 |  | 30.48(18.25 to 48.37) | 32.35(18.82 to 50.6) |  | 0.13(0.11 to 0.14) |
| Sao Tome and Principe | 0.01(0.01 to 0.02) | 0.02(0.01 to 0.03) |  | 68.06 |  | 41.98(24.53 to 65.89) | 42.96(25.44 to 67.85) |  | 0.06(0.05 to 0.06) |
| Saudi Arabia | 1.23(0.75 to 1.95) | 4.31(2.63 to 6.66) |  | 249.29 |  | 40.82(24.79 to 64.43) | 42.73(26 to 65.61) |  | 0.15(0.14 to 0.16) |
| Senegal | 0.66(0.4 to 1.03) | 1.53(0.88 to 2.39) |  | 130.20 |  | 42.04(25.7 to 64.67) | 43.07(25.01 to 66.98) |  | 0.09(0.07 to 0.1) |
| Serbia | 3.41(2.14 to 5.12) | 5.48(3.43 to 8.16) |  | 60.70 |  | 66.92(42.43 to 99.75) | 70.95(44.46 to 105.65) |  | 0.18(0.17 to 0.19) |
| Seychelles | 0.02(0.01 to 0.03) | 0.05(0.03 to 0.07) |  | 136.18 |  | 80.24(48.24 to 124.04) | 84.45(51.41 to 132.71) |  | 0.14(0.12 to 0.16) |
| Sierra Leone | 0.42(0.25 to 0.66) | 0.74(0.45 to 1.16) |  | 76.96 |  | 40.71(24.03 to 63.92) | 41.78(25.21 to 66.72) |  | 0.08(0.07 to 0.09) |
| Singapore | 0.2(0.11 to 0.31) | 0.9(0.52 to 1.45) |  | 361.70 |  | 20.43(11.85 to 32.7) | 20.66(11.85 to 32.94) |  | 0.06(0.04 to 0.08) |
| Slovakia | 1.7(1.05 to 2.42) | 2.94(1.81 to 4.25) |  | 72.87 |  | 66.56(41.33 to 94.24) | 69.28(42.71 to 100) |  | 0.12(0.12 to 0.13) |
| Slovenia | 0.43(0.27 to 0.62) | 0.96(0.61 to 1.42) |  | 122.54 |  | 45.17(28.44 to 66.01) | 47.37(30.1 to 70.11) |  | 0.11(0.09 to 0.13) |
| Solomon Islands | 0.04(0.03 to 0.07) | 0.1(0.06 to 0.16) |  | 132.28 |  | 68.55(40.15 to 105.65) | 71.97(42.42 to 109.7) |  | 0.19(0.12 to 0.25) |
| Somalia | 0.42(0.25 to 0.66) | 0.99(0.57 to 1.52) |  | 136.45 |  | 44.94(26.66 to 70.67) | 45.16(26.86 to 70.35) |  | 0.18(0.16 to 0.2) |
| South Africa | 5.1(3.05 to 7.98) | 11.92(7.14 to 18.55) |  | 133.90 |  | 61.71(36.62 to 96.43) | 63.07(37.78 to 98.24) |  | 0(-0.01 to 0.01) |
| South Sudan | 0.67(0.39 to 1.06) | 0.82(0.48 to 1.25) |  | 23.15 |  | 44.64(26.45 to 70.23) | 44.63(26.65 to 69.75) |  | 0.07(0.05 to 0.1) |
| Spain | 5.9(3.45 to 9.19) | 13.17(7.94 to 20.71) |  | 123.39 |  | 23.95(14.03 to 37.09) | 31.41(18.86 to 50.16) |  | -0.01(-0.03 to 0.01) |
| Sri Lanka | 4.1(2.46 to 6.28) | 10.37(6.22 to 15.81) |  | 153.02 |  | 80.09(48.44 to 122.28) | 83.49(50.45 to 127.07) |  | 0.53(0.38 to 0.68) |
| Sudan | 1.9(1.13 to 2.98) | 4.19(2.48 to 6.6) |  | 120.65 |  | 40.39(23.86 to 62.98) | 42.52(25.1 to 67) |  | 0.14(0.13 to 0.16) |
| Suriname | 0.07(0.04 to 0.11) | 0.18(0.11 to 0.28) |  | 158.18 |  | 58.3(34.23 to 92.28) | 61.98(37.41 to 100.05) |  | 0.13(0.11 to 0.14) |
| Sweden | 3.1(1.85 to 4.96) | 4.13(2.4 to 6.6) |  | 33.07 |  | 45.86(27.26 to 73.63) | 40.81(24 to 65.46) |  | 0.25(0.22 to 0.27) |
| Switzerland | 4.15(2.54 to 6.04) | 8.15(5.12 to 11.79) |  | 96.24 |  | 94.26(57.69 to 136.9) | 98.22(61.8 to 142.14) |  | -0.12(-0.21 to -0.02) |
| Syrian Arab Republic | 1.05(0.62 to 1.64) | 2.87(1.69 to 4.39) |  | 171.97 |  | 40.82(23.65 to 63.48) | 42.41(24.89 to 64.69) |  | 0.14(0.13 to 0.14) |
| Taiwan (Province of China) | 6.09(3.51 to 9.41) | 16.14(9.84 to 24.59) |  | 165.07 |  | 78.04(46.42 to 118.38) | 80.54(49.54 to 122.28) |  | 0.08(0.07 to 0.09) |
| Tajikistan | 0.6(0.36 to 0.93) | 1.47(0.87 to 2.33) |  | 143.52 |  | 55(32.76 to 85.77) | 56.68(33.52 to 88.32) |  | 0.09(0.08 to 0.1) |
| Thailand | 11.92(7.21 to 18.65) | 41.17(25.37 to 62.98) |  | 245.52 |  | 78.83(47.29 to 120.92) | 81.22(49.59 to 123.97) |  | 0.1(0.08 to 0.12) |
| Timor-Leste | 0.09(0.05 to 0.15) | 0.34(0.2 to 0.52) |  | 267.98 |  | 78.29(46.22 to 124.23) | 81.08(48.35 to 122.22) |  | 0.04(0.02 to 0.06) |
| Togo | 0.22(0.13 to 0.34) | 0.64(0.38 to 0.99) |  | 185.13 |  | 41.29(24.2 to 63.82) | 42.36(24.92 to 66.11) |  | 0.18(0.16 to 0.21) |
| Tokelau | 0(0 to 0) | 0(0 to 0) |  | 11.15 |  | 69.13(39.98 to 107.36) | 71.59(42.34 to 109.55) |  | 0.09(0.08 to 0.1) |
| Tonga | 0.02(0.01 to 0.03) | 0.03(0.02 to 0.04) |  | 45.94 |  | 69.47(40.58 to 108.37) | 71.88(42.34 to 110.47) |  | 0.05(0.03 to 0.06) |
| Trinidad and Tobago | 0.24(0.14 to 0.38) | 0.6(0.36 to 0.98) |  | 152.35 |  | 60.02(36.01 to 95.86) | 62.29(37.3 to 101.51) |  | 0.15(0.13 to 0.17) |
| Tunisia | 1.06(0.63 to 1.68) | 2.77(1.64 to 4.25) |  | 161.25 |  | 40.93(24.32 to 63.61) | 42.44(25.23 to 65.02) |  | 0.13(0.11 to 0.14) |
| Turkey | 6.3(3.63 to 9.8) | 18.37(10.92 to 29) |  | 191.73 |  | 40.7(23.82 to 63.77) | 41.63(24.83 to 65.71) |  | 0.13(0.11 to 0.15) |
| Turkmenistan | 0.39(0.23 to 0.61) | 0.91(0.53 to 1.41) |  | 132.91 |  | 54.96(32.85 to 86.17) | 56.1(32.22 to 85.56) |  | 0.11(0.1 to 0.12) |
| Tuvalu | 0(0 to 0) | 0(0 to 0.01) |  | 78.04 |  | 69.23(40.33 to 107.15) | 72.7(43.34 to 111.79) |  | 0.1(0.08 to 0.12) |
| Uganda | 1.33(0.79 to 2.11) | 2.65(1.59 to 4.2) |  | 98.90 |  | 44.97(26.52 to 70.26) | 45.63(27.52 to 71.61) |  | 0.07(0.05 to 0.1) |
| Ukraine | 31.29(19.21 to 47.42) | 37.6(22.41 to 56.95) |  | 20.16 |  | 124.85(76.77 to 188.99) | 124.78(74.91 to 187.72) |  | 0.18(0.16 to 0.19) |
| United Arab Emirates | 0.09(0.05 to 0.14) | 1.33(0.78 to 2.12) |  | 1384.54 |  | 41.9(25.03 to 64.8) | 44(26.52 to 68.97) |  | 0.02(0 to 0.03) |
| United Kingdom | 19.08(12.08 to 28.89) | 30.33(19.23 to 45.81) |  | 58.95 |  | 48.43(30.71 to 72.9) | 49.98(31.55 to 75.25) |  | 0.02(0 to 0.05) |
| United Republic of Tanzania | 2.28(1.35 to 3.65) | 5.17(3.05 to 8.11) |  | 126.99 |  | 44.58(26.43 to 70.05) | 45.4(26.92 to 72.06) |  | 0.15(0.12 to 0.17) |
| United States of America | 49(30.88 to 74.1) | 95.93(60.59 to 138.07) |  | 95.78 |  | 36.41(22.96 to 54.68) | 35.42(22.4 to 51.04) |  | 0.31(0.25 to 0.37) |
| United States Virgin Islands | 0.02(0.01 to 0.03) | 0.06(0.03 to 0.09) |  | 164.51 |  | 57.77(34.51 to 90.85) | 60.73(36.59 to 96.01) |  | 0.05(0.04 to 0.06) |
| Uruguay | 0.42(0.25 to 0.66) | 0.6(0.35 to 0.91) |  | 40.68 |  | 24.12(14.06 to 37.33) | 25.55(14.94 to 39.26) |  | 0.2(0.12 to 0.27) |
| Uzbekistan | 2.38(1.4 to 3.72) | 6.22(3.68 to 9.69) |  | 161.24 |  | 54.68(32.03 to 86.22) | 56.36(33.48 to 86.17) |  | 0.19(0.17 to 0.22) |
| Vanuatu | 0.02(0.01 to 0.03) | 0.05(0.03 to 0.08) |  | 165.51 |  | 68.86(39.7 to 104.87) | 71.8(41.94 to 110.77) |  | 0.26(0.2 to 0.32) |
| Venezuela (Bolivarian Republic of) | 3.59(2.15 to 5.54) | 12.32(7.45 to 19.58) |  | 243.31 |  | 85.87(52.06 to 130.61) | 90.11(55.44 to 141.89) |  | 0.09(0.07 to 0.1) |
| Viet Nam | 14.01(8.28 to 21.37) | 39.9(24.34 to 60.45) |  | 184.80 |  | 85.55(50.85 to 130.5) | 95.91(58.48 to 146.52) |  | 0.17(0.15 to 0.19) |
| Yemen | 0.87(0.5 to 1.37) | 2.62(1.55 to 4.09) |  | 202.44 |  | 40.09(23.3 to 63.54) | 41.32(24.55 to 64.61) |  | 0.08(0.07 to 0.09) |
| Zambia | 0.63(0.37 to 0.98) | 1.32(0.8 to 2.05) |  | 110.34 |  | 44.96(26.66 to 70.01) | 45.26(27.53 to 71.13) |  | 0(0 to 0.01) |
| Zimbabwe | 1.09(0.64 to 1.7) | 1.55(0.93 to 2.42) |  | 42.87 |  | 56.62(33.94 to 88.34) | 58.6(35.6 to 90.12) |  | 0.1(0.09 to 0.12) |

DALYs: disability-adjusted life-years; EAPC: estimated annual percentage change; UI: uncertainty interval; CI: confidence interval.
