# Supplementary material for: Global, regional, and national burden of benign prostatic hyperplasia from 1990 to 2021 and projection to 2035
Source: BMC Urol. 2025 Feb 19;25:34. doi: 10.1186/s12894-025-01715-9 (PMC11837592; doi:10.1186/s12894-025-01715-9)
Supplement: Supplementary file 4 — Supplementary Material 4 [file 12894_2025_1715_MOESM4_ESM.docx]

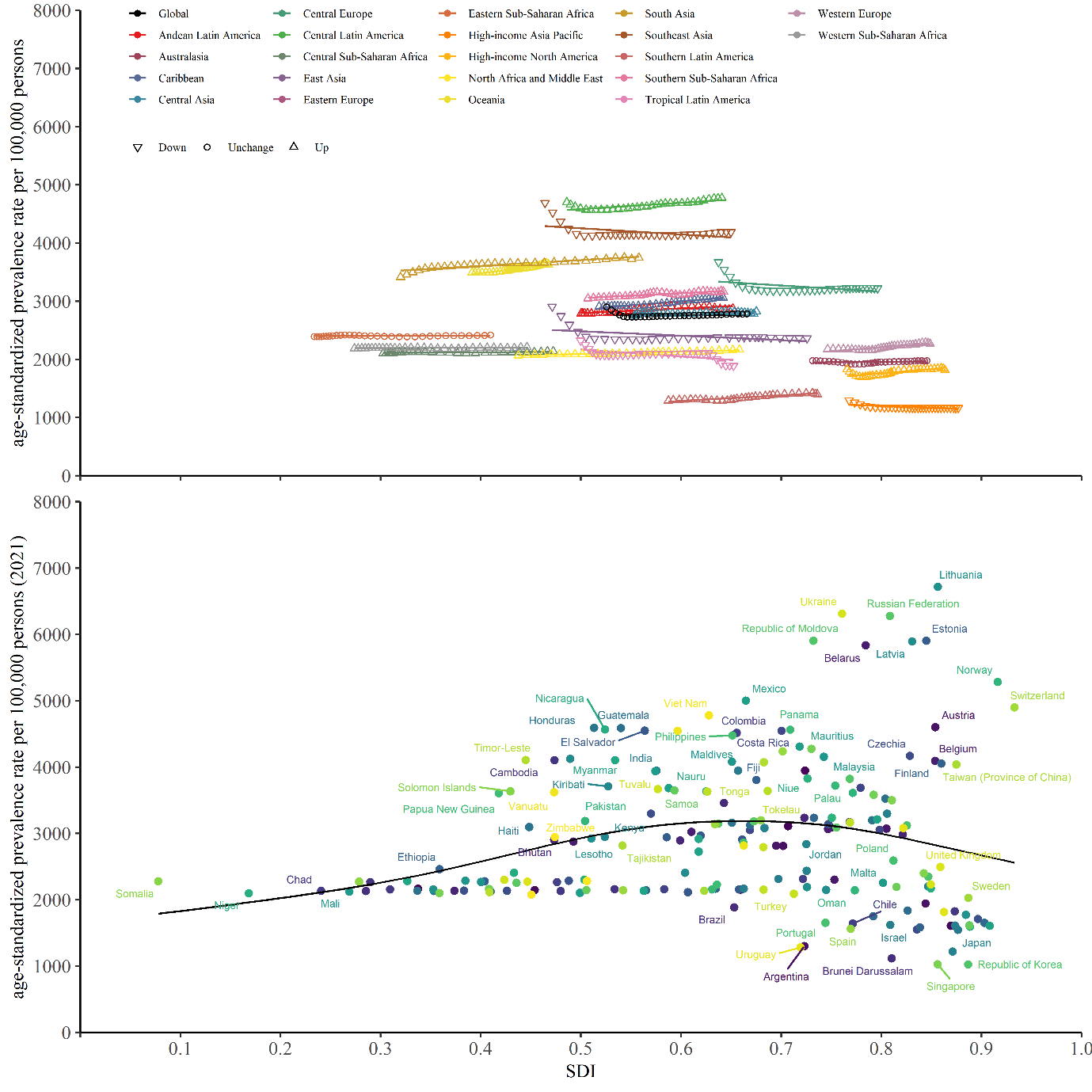


**Figure S1.** The ASPR of BPH for 21 regions (a) and 204 countries and territories (b) by SDI. a, 21 regions by SDI from 1990 to 2021. b, 204 countries and territories by SDI in 2021. ASPR, age-standardized prevalence rate; BPH, benign prostatic hyperplasia.


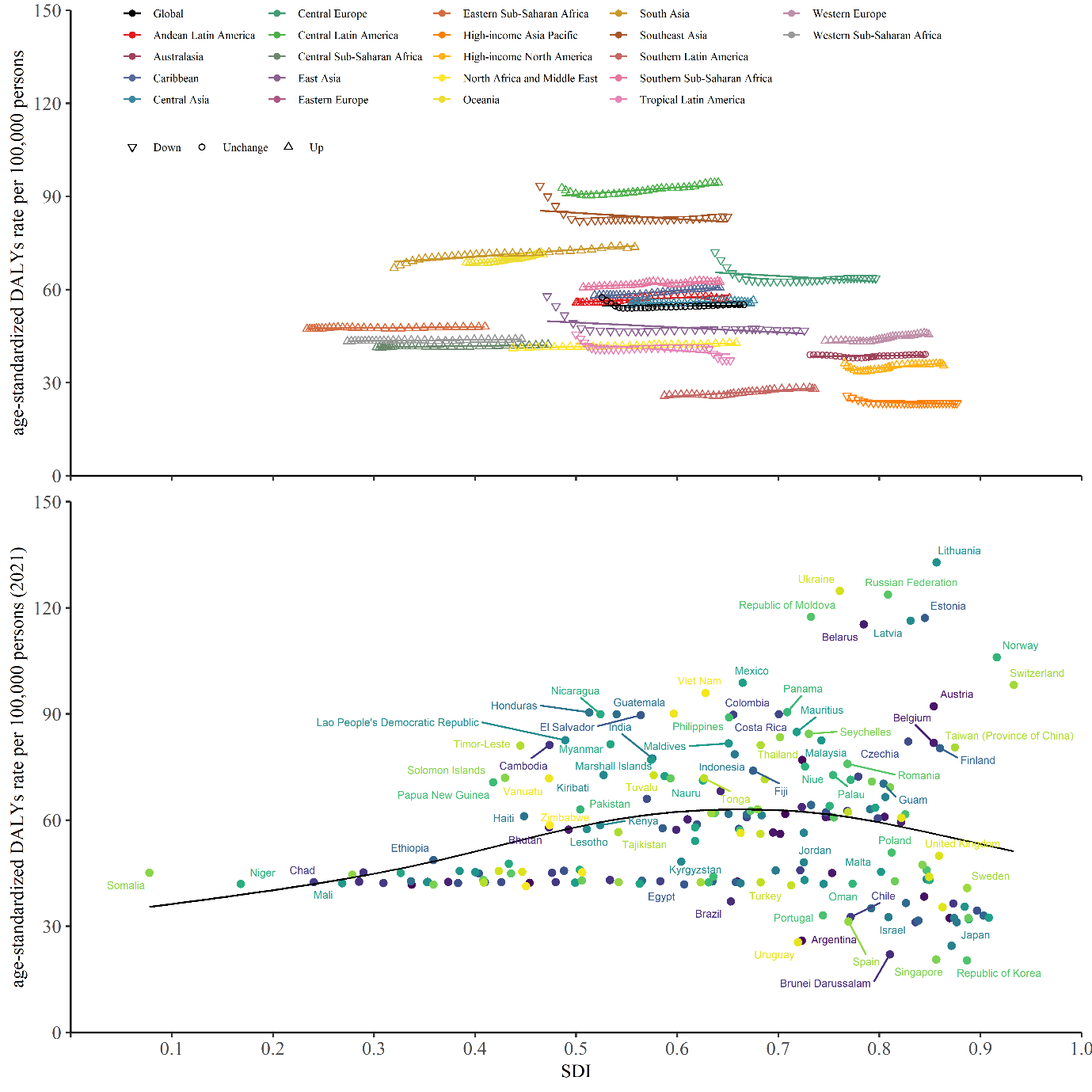


**Figure S2.** The ASDR of BPH for 21 regions (a) and 204 countries and territories (b) by SDI. a, 21 regions by SDI from 1990 to 2021. b, 204 countries and territories by SDI in 2021. ASDR, age-standardized DALYs rate; DALYs, disability-adjusted life-years; BPH, benign prostatic hyperplasia.
